# Stability and Electronic Structure of Nitrogen-Doped Graphene-Supported Cu_*n*_ (*n* = 1–5) Clusters in Vacuum and under Electrochemical Conditions: Toward Sensor and Catalyst Design

**DOI:** 10.1021/acs.jpcc.3c06475

**Published:** 2024-03-08

**Authors:** Márton Guba, Tibor Höltzl

**Affiliations:** † Department of Inorganic and Analytical Chemistry and HUN-REN-BME Computation Driven Chemistry Research Group, Budapest University of Technology and Economics, Szent Gellért tér 4, Budapest H-1111, Hungary; ‡ Nanomaterials Science Group, Furukawa Electric Institute of Technology, Késmárk utca 28/A, Budapest H-1158, Hungary

## Abstract

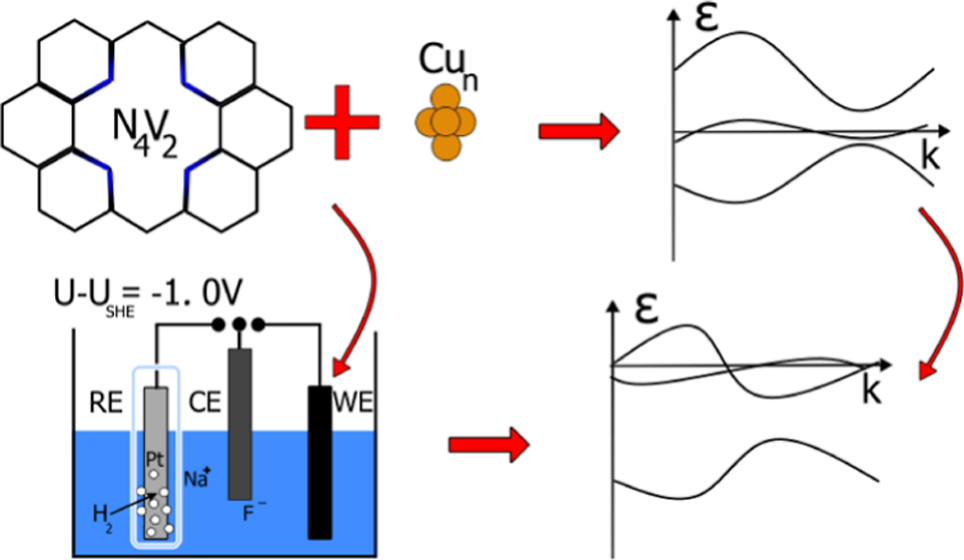

Here, we present a detailed computational study of the stability and the electronic structure of nitrogen-doped graphene (N_4_V_2_) supported Cu_*n*_ (*n* = 1–5) clusters, which are promising carbon-dioxide electroreduction catalysts. The binding of the clusters to the nitrogen-doped graphene and the electronic structure of these systems were investigated under vacuum and electrochemical conditions. The stability analysis showed that among the systems, the nitrogen-doped graphene bound Cu_4_ is the most stable in vacuum, while in an electrolyte, and at a negative potential, the N_4_V_2_–Cu_3_ is energetically more favorable. The ground state electronic structure of the nitrogen-doped graphene substrate undergoes topological phase transition, from a semimetallic state, and we observed a metallic and topologically trivial state after the clusters are deposited. The electrode potential adjusts the type and density of the charge carriers in the semimetallic models, while the structures containing copper exhibit bands which are deformed and relaxed by the modified number of electrons.

## 1 Introduction

Nowadays metal clusters deposited on nitrogen-doped graphene have attracted much attention both as catalysts and as sensor materials. The important target is the electrochemical reduction of the carbon-dioxide (CO_2_)^1,2^ toward C_1_^3,4^ (e.g., methanol) or even C_2_ products^5−7^ (e.g., ethanol). The catalytic conversion contains a series of chemical reaction steps which require catalysts to lower the energy barriers.^8,9^ Clusters containing a few metal atoms turned out to have outstanding catalytic activity^10−12^ originated not only from their large surface-volume ratio but also due to quantum confinement, i.e., their electronic structures are built up by discrete energy levels, whose occupations influence strongly their reactivity.^13^

While small transition metal clusters can be effective catalysts of CO_2_ dissociation in the gas phase, for practical applications, the clusters are beneficial to be deposited on a support, which stabilizes them and enhances their activity.^14−16^ Graphene is an attractive candidate, since it is chemically inert, mechanically elastic and have high electric conductivity.^17^ Vacancies^18,19^ and dopants, like boron^20^ and nitrogen^21−23^ play an important role in binding metal particles and clusters.^24,25^ Hamamoto et al.^26^ showed decreased carbon-monoxide (CO) poisoning (indicated by weakened CO binding energy compared to other platinum-based systems) of Pt_4_ decorated vacancies in graphene, which is crucial for the design of platinum-based catalysts. Du et al.^27^ studied the electrochemical carbon-dioxide conversion to methanol on Cu_3_ decorated single vacancy in graphene, while the enhancement of catalytic activity was shown by Shi et al.^28^ for nitrogen-doped graphene-supported Cu_4_ cluster. Barhács et al.^29^ demonstrated the formation of C_1_ and C_2_ products on boron-doped graphene nanoflake-supported copper clusters. Nitrogen-doped graphene bound cluster-based catalysts can be effective in other important reactions as well. Zang et al. has shown that the energy-intensive Haber–Bosch process for the reduction of nitrogen to ammonia can be replaced by the copper atom embedded pyridine-type defect in graphene.^30^

The electronic structure of metal clusters is also sensitive to any adsorbates, which makes them promising candidates for electro- and biochemical sensor applications as well.^31^ Transition metal cluster-decorated graphene substrate turned out to be promising for applying it as an electrochemical sensor.^32,33^ Gold–copper clusters deposited on nitrogen-doped graphene-based quantum dots were demonstrated by Saisree et al.^34^ to be selective materials for detecting glycine under electrochemical conditions, while they also showed that its copper decorated analogues are possible candidates for sensing dopamine, serotonin, or nicotine.^35^ Yang et al. investigated the tunability of palladium decorated graphene, which can be used as formaldehyde detector.^36^ Libeert et al. showed the high sensitivity of graphene-supported Au_3_ cluster for observing the ad- and desorption kinetics of oxygen molecules.^37^

The stability analysis of the support anchored clusters helps to select the most prominent models both for the catalyst and sensor material.^38,39^ Here, the accurate description of the interaction between the graphene-based support and the clusters is essential, which requires the inclusion of spatially long-range terms.^40^ The detailed study of the electronic structure properties (e.g., band structure, occupation etc.) and the derived quantities (e.g., conductivity) are relevant from both catalytic effects (e.g., by opening electron transfer channels) and sensor efficiency (e.g., sensitivity of the electronic structure on the adsorbate binding).^41,42^ The understanding of the effect of the electrode potential on the tuneability of the electronic states is crucial for the catalyst design.^43,44^

Here, the usual and widespread theoretical description is the computational hydrogen electrode model.^45^ However, this does not consider the evolution of the electronic states at different potentials. We applied the grand canonical theory (GCP-K), developed by Goddard et al.,^46^ where the total number of electrons depend on the applied electrode potential. This makes it possible to model the geometry and electronic structure modifications occurring due to the electrode potential and examine the voltage-dependent evolution of the cluster’s stability.

Here, we investigate the Cu_*n*_ (*n* = 1–5) clusters bound to pyridine-type, N_4_V_2_^47^ defect in graphene both in vacuum and under electrochemical conditions. N_4_V_2_ can be synthesized^48,49^ and, furthermore, have been carefully studied both experimentally^50^ and theoretically.^51^ We applied density functional theory (DFT) computations for studying the stability trends and the interaction between clusters and the defected graphene. We also study the electronic structures using advanced symmetry considerations. Murugesan et al.^52^ studied Cu_*n*_ (*n* = 1–5) clusters deposited on free and single vacancy-defected graphene surface using DFT computations. They focused on possible applications in the field of spintronics.

The paper is organized as follows. In Section 2, we list the technical details of the stability and electronic structure computations. Section 3 is divided into two main parts. First, the ground state geometries of N_4_V_2_–Cu_*n*_ (*n* = 1–5) will be discussed, and we analyze several stability descriptors and the electronic structure. These serve as references for the second part of our work, where we discuss the geometrical and stability modifications of the models under electrochemical conditions. Most importantly, the robustness and possible deformations of the electronic structure will be focused on.

## 2 Methods

The computations presented in this paper are based on DFT^53^ using the PBE functional^54^ and the D3 dispersion correction of Grimme.^55^ All nonelectrochemical computations were carried out using the GPAW^56^ program in conjunction with the Atomic Simulation Environment.^57^ The interaction between atomic nuclei and the core/valence electrons were treated with projector augmented waves (PAWs) (using data sets released in 2016^58^). The computations were spin-polarized, and the total magnetic moment was relaxed. Linear combination of atomic orbitals (LCAOs),^59^ including s- and p-type Gaussian-functions with d-type polarization function on C and N atoms, and furthermore, s-, p-, and d-type Gaussian-functions with d-type polarization function for Cu atoms with real space grid spacing *h* = 0.1 Å and Monkhorst–Pack momentum space sampling scheme^60^ (4, 4, 1) + Γ were applied for the geometry optimizations. Fermi–Dirac distribution with 0.1 eV broadening was applied. Structure relaxations were carried out using the limited-memory Broyden–Fletcher–Goldfarb–Shanno^61^ and the fast inertial relaxation engine^62^ algorithms with *f*_max_ = 0.01 eV/Å final constraint.

Electronic structure analysis was performed with similar parameters except that a finer *k*-point grid (10, 10, 1) + Γ was selected to obtain more accurate results. Gaussian-broadening of 0.4 eV was applied in the visualization of the density of states (DOS). Final energy computations were performed on the LCAO optimized geometries using plane wave (PW) basis set with 1000 eV cutoff energy.

Further simulations were performed to study the effect of electrochemical environment using jDFTx software developed by Sundararaman et al.^63^ We used ultrasoft pseudopotentials built-up by Garrity et al.^64^ to describe electron–core interactions. Here, for PW basis and charge density cutoff energies, the default values of 544 and 2721 eV (originally these values are given in atomic unit and have been converted into eV for the sake of consistency) were employed. The aqueous electrolyte of potassium-fluorite with an ionic concentration of 1 mol/L was applied. The electrolyte was modeled using the linear, implicit solvation methodology, specifically the charge-asymmetric nonlocally determined local-electric model,^65^ and the ions dissolved in the electrolyte are treated using the linearized Poisson–Boltzmann equation.^66^ We also applied the electrode potential *U*, measured from the potential of the standard hydrogen electrode (SHE). Its value was *U*_SHE_ = −4.66 ± 0.11 V after Sundararaman et al.^65^ The geometries and the simulation cell were the same as in the gas-phase, but here no spin-polarization was applied. (5, 5, 1) *k*-point grid in MP scheme was found to be suitable for optimization and stability analysis.

## 3 Results and Discussion

### 3.1 Equilibrium Geometries and Stability Analysis

**Figure 1:**
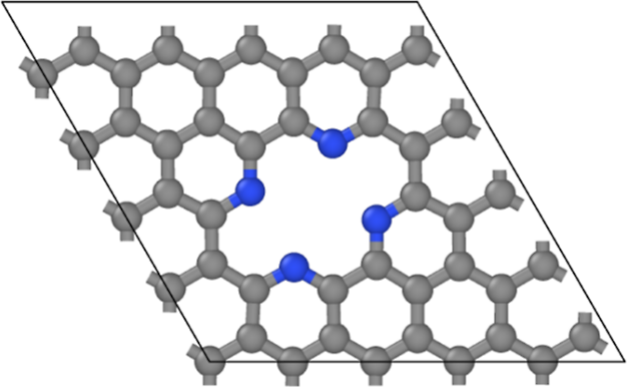
Pyridine-type defect in a 5 × 5 supercell of graphene (N_4_V_2_). The simulation cell in the periodic directions is also shown. Colors: gray—carbon and blue—nitrogen.

The model system was set up from a 5 × 5 graphene supercell including a double vacancy in the center, where four of the carbon atoms were substituted by nitrogens (denoted hereafter as N_4_V_2_), as shown in Figure 1. The model was selected based on its high thermodynamical stability, showed in ref (51). The geometry itself was originally imported from ref (67), where the cell had been already optimized, hence only the positions of the atoms were relaxed, while the cell vectors were kept fixed. The size of the vacuum (size of that cell vector) in the direction of the surface normal of the substrate was selected to be 12 Å.

Subsequently, small Cu_*n*_ clusters (*n* = 1–5) were deposited on N_4_V_2_.

**Figure 2:**
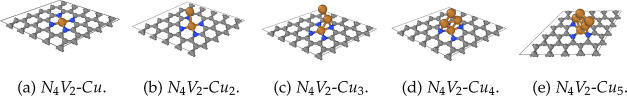
Ground state structure of N_4_V_2_–Cu_*n*_ (*n* = 1–5). The simulation cell in the periodic directions is also shown. Colors: gray—carbon, blue—nitrogen, and orange—copper.

We investigated different possible cluster-surface binding configurations, from which the lowest energy structures are presented in Figure 2. As expected, the computations show that copper atom is preferentially coordinated to the center of the pyridine-type defect.

Furthermore, the form and shape of the final geometries also suggest that the copper atoms preferentially bound to several nitrogen atoms. In addition to the copper atom, located at the center, one of the copper atoms of clusters Cu_2_ and Cu_3_ also binds to one of the nitrogens, while in case of Cu_4_ and Cu_5_ two copper atoms coordinate to nitrogens. The interaction between N_4_V_2_ and the clusters leads to the bending of the plane of the defected graphene, thereby changing its electronic structure, as will be shown later in our paper. It is worth comparing the Cu_*n*_ cluster binding modes on N_4_V_2_ to those on graphene. Murugesan et al.^52^ showed that Cu_*n*_ (*n* = 1–4) clusters prefer to bind to pristine graphene with a single copper atom with planar geometry, where the plane of the cluster is perpendicular to the one of the substrate. On the other hand, the plane of cluster Cu_5_ is parallel to the one of graphene. The interaction between the clusters and the single vacancy leads to the distortion of the graphene’s plane (similar to our case) close to the defect site, where the Cu_*n*_ clusters are adsorbed. The energetics of the cluster binding to N_4_V_2_ was quantified using four different descriptors.

- Interaction energy

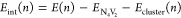
1describes how beneficial is the interaction between the cluster and structure N_4_V_2_ in energy. Here, *E*(*n*) gives the total electronic energy of N_4_V_2_–Cu_*n*_ (*n* = 1–5) and  and *E*_cluster_(*n*) are the total energies of the free N_4_V_2_ surface and Cu_*n*_ cluster, respectively.

- Second order interaction energy

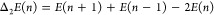
2gives the relative energy of N_4_V_2_–Cu_*n*_ (*n* = 1–5) with *n* copper atoms respect to those with *n* + 1 and *n* – 1 atoms. Positive Δ_2_*E*(*n*) indicates extended stability of N_4_V_2_–Cu_*n*_ compared to the systems involving the neighboring cluster sizes.

- Gained energy^68^

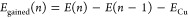
3signifies how much energy is gained by N_4_V_2_–Cu_*n*_ if an extra copper atom is added. Here, the meaning of the first two terms have been already defined in eq 2, while *E*_Cu_ represents the total energy of a single copper atom.

- Cohesive energy^68^

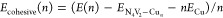
4characterizes the cohesion between cooper atoms of the cluster in the presence of N_4_V_2_.

**Figure 3:**
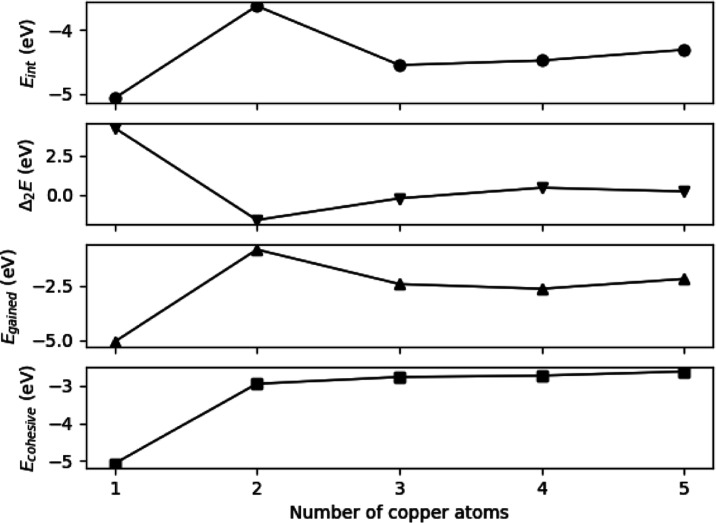
Stability descriptors for N_4_V_2_–Cu_*n*_ (*n* = 1–5) structures under vacuum conditions. See the text for the definitions.

The stability of the relaxed structures was explored using these descriptors, and they are depicted on Figure 3 as a function of the cluster’s size. The different descriptors show that the single copper atom coordinated to the pyridinic nitrogen vacancy is the most stable among the studied systems. The shapes of *E*_int_(*n*) and E_gained_(*n*) are similar, although the latter shows higher stability of Cu_4_, than that of Cu_3_. This is also confirmed by the second order energy, which differs considerably from the result of the calculations about the free clusters in gas phase.^69^ There, Δ_2_*E*(*n*) varies nonmonotonically with respect to the cluster size, showing higher stability in the case of certain clusters. Compared to that, here, the significant difference in stability is due to the interaction of the cluster with N_4_V_2_, where nitrogen atoms prefer to withdraw electrons from the copper atoms. The Bader charge analysis showed that among the clusters, N_4_V_2_–Cu_4_ exhibits the largest charge transfer, Δ*Q* = 1.39e^–^ and also the highest number of copper–nitrogen bonds, in line with its extended stability. It is worth noting that the interaction energies between the substrates and the metal clusters are roughly twice as high with nitrogen-doped graphene compared to pristine graphene or graphene with a vacancy.^52^ Furthermore, on pristine and on single vacancy defected graphene, the Cu_3_ and Cu_5_ were found to be the most stable, respectively.^52^

### 3.2 Ground State Electronic Structure

**Figure 4:**
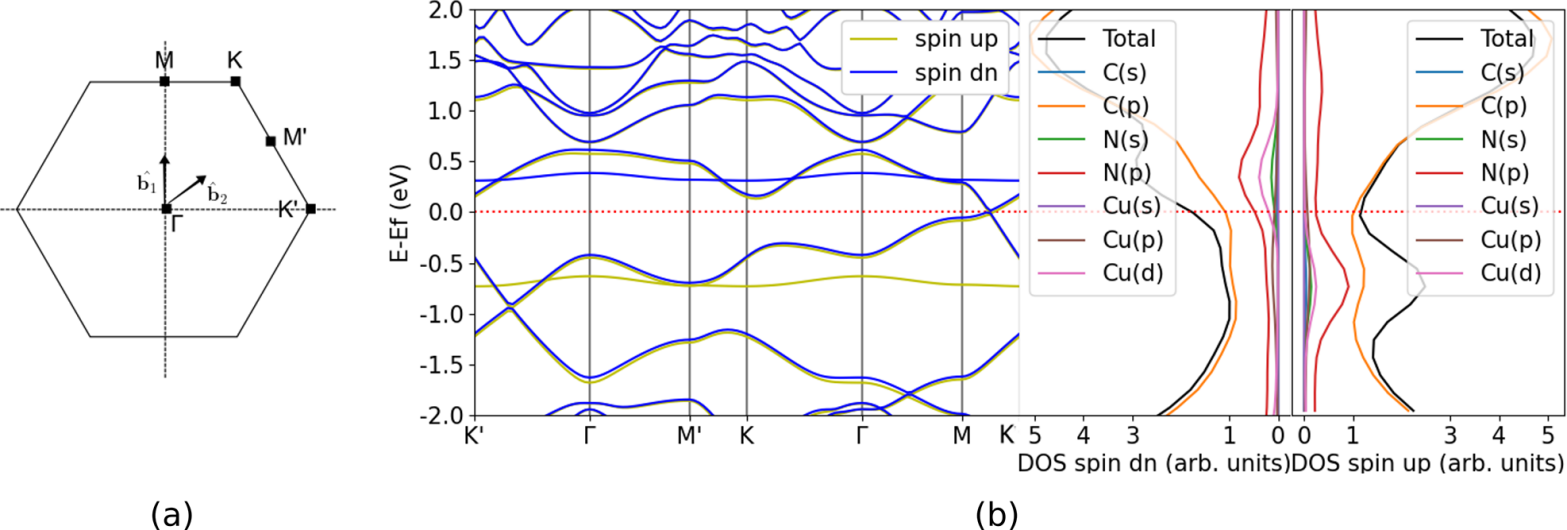
First Brillouin-zone, band structure, and total and orbital projected DOS of N_4_V_2_–Cu.

We studied the ground state electronic structure properties, specifically band structures and total and orbital projected DOS (PDOS) of N_4_V_2_–Cu_*n*_ (*n* = 1–5), to understand how the electronic states of N_4_V_2_ evolve when clusters are deposited. Graphene is a topological semimetal, i.e., it has a nontrivial topology related to the existence of its Dirac-points. As long as its chiral symmetry is not broken, the Dirac-points will not split, but can be relocated in the Brillouin-zone.^70^ In line, refs (67, 71, and 72) showed, that the Dirac-points do not split up when the pyridine-type defect is introduced; however, the point group of graphene (*D*_6*h*_) reduces to *D*_2*h*_. We discuss N_4_V_2_–Cu first, whose band structure and DOS are depicted in Figure 4.

**Figure 5:**
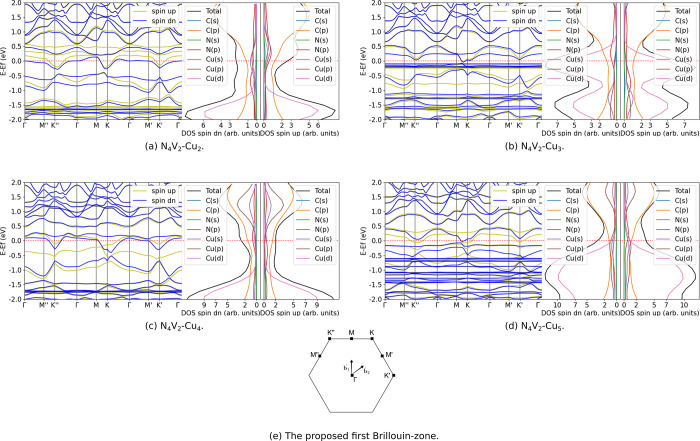
Band structure, total and orbital projected DOS, and the proposed first Brillouin-zone of N_4_V_2_–Cu_*n*_ (*n* = 2–5).

As the presence of the copper dopant does not change the symmetry properties of the nitrogen-doped, defected graphene, the Dirac-point stays between the points *K*′ and Γ and remains knotted, in line with the preserved chiral symmetry. The interaction with the copper atom shifts the Fermi-level toward the conduction band. This can be connected to the significant charge transfer occurring from the copper atom to N_4_V_2_, leading to more metallic than a semimetallic phase. We also observed large splitting (of 1 eV) of the defect band formed by the p-orbitals of nitrogen and d-orbitals of copper, due to spin-polarization. A significant difference appears if a Cu_*n*_ (*n* = 2–5) cluster interacts with the N_4_V_2_, as it was referred to in Section 3.1, the multiple copper–nitrogen bonds bend the plane of the substrate. Thereby, the Dirac-cone, formed by the hybridization of the p-orbitals of the carbon atoms, is also deformed. Hence, we can expect to have a chiral symmetry-breaking leading to a topological transition between nontrivial and trivial phases. Figure 5a–d confirms the previous implication.

The careful mapping of the Brillouin-zone of N_4_V_2_–Cu_*n*_ (*n* = 2–5) was performed, and considerable reduction of symmetry was observed. We found that the models have C_1_ symmetry, while the approximate inversion symmetry (Figure 5e) can be presumed only within a tolerance. The interaction of N_4_V_2_ with the clusters shifts the Fermi-level of the whole system toward the conduction band, compared to the case of N_4_V_2_. Due to the chiral-symmetry breaking, the Dirac-points disappear, and the N_4_V_2_–Cu_*n*_ (*n* = 2–5) turns into a metallic phase. Significant number of spin-polarized, flat bands can be observed, which are mainly formed by the copper d-orbitals and appear both in the valence and conduction bands. For instance, while they are presented in the valence band relatively far from the Fermi-level in the case of clusters Cu_2_ (Figure 5a) and Cu_4_ (Figure 5c), Cu_3_ (Figure 5b) and Cu_5_ (Figure 5d) have them in the valence bands, close to the Fermi-level. We could also expect the models containing even number of copper atoms (thus even number of electrons) to have singlet ground state. Still, the PDOS indicates that the bands having contributions from copper and nitrogen atoms are asymmetric in spin, i.e., these models have polarized ground state. Murugesan et al.^52^ investigated the band structure of pristine and single vacancy defected graphene supported Cu_*n*_ (*n* = 1–5); however, the path in the first Brillouin-zone differs from the one we studied. Still, the comparison of the results of N_4_V_2_ with those for pure graphene shows that due to the different type of structures, the shape of band structures deviates. This is not only valid for the cluster-decorated case but also for the pristine defected graphene.^73^ However, it is important to note that flat bands formed by the copper d-orbitals also appear in the case of graphene supported copper clusters.^52^ In summary, the partially occupied bands near the Fermi-level could indicate a metallic phase; however, these bands are flatter than the Dirac cone, showing an exciting example about the hybridization of spatially localized (clusters) and extended (N_4_V_2_) electronic states.

### 3.3 Geometries and Energetics under Electrochemical Conditions

We examined the stability of the N_4_V_2_–Cu_*n*_ (*n* = 1–5) structures under electrochemical conditions, where both the electrolyte and the effect of an electrode potential, *U* is considered. This can be modeled using the grand canonical ensemble, where the number of the charge carriers *N* can change, i.e., if the chemical potential of the electrons (μ) is increased or decreased, there will be charge flow into or out of the system, respectively. The special case is when *N* is fixed and μ is equilibrated. We used the grand canonical potential kinetics (GCP-K) model,^46,74,75^ one of the state-of-the-art methods for describing electrochemical reactivity.

We investigated the effects of the electrolyte and the electrode potential on the geometries. Subsequently, we performed computations with the total number of electrons corresponding to *U* – *U*_SHE_. Here, geometry optimizations were carried out for potentials *U* – *U*_SHE_ = ±1.0 V and the potential of zero charge (PZC). The latter one can be determined in two ways: the charge neutral jDFTx computation provides it as the converged value of the chemical potential, which is transformed into PZC (electrochemical potential) using the reference value of *U*_SHE_. Otherwise, one of the parameters used in the GCP-K formalism can be converted into PZC as well. Based on the latter one, we listed the PZC values of N_4_V_2_–Cu_*n*_ (*n* = 1–5) in Table 1.

**Table 1:** Potential of Zero Charge of N_4_V_2_–Cu_*n*_ (*n* = 1–5), Determined by Using the GCP-K Method

structure	Potential of zero charge—PZC (V)
N_4_V_2_–Cu	–0.72
N_4_V_2_–Cu_2_	–0.86
N_4_V_2_–Cu_3_	–0.69
N_4_V_2_–Cu_4_	–0.97
N_4_V_2_–Cu_5_	–0.83

**Figure 6:**
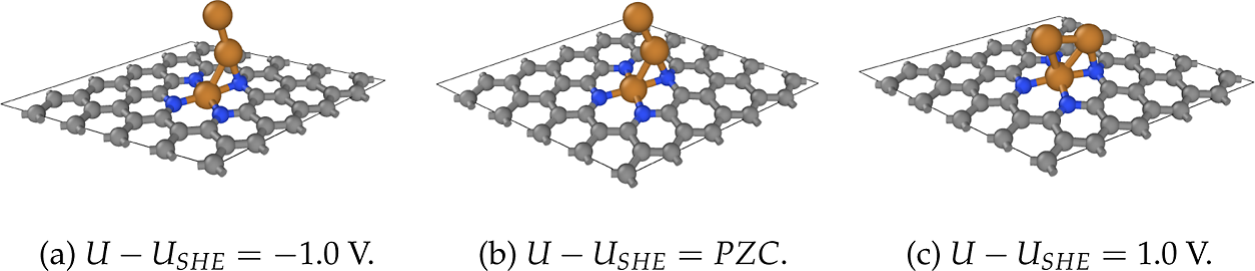
Geometry of N_4_V_2_–Cu_3_ after relaxation at *U* – *U*_SHE_ = ±1.0 and *U* – *U*_SHE_ = PZC = −0.69 V potentials. The simulation cell in the periodic directions is also shown.

The results of the optimizations are presented in Figure 6 for N_4_V_2_–Cu_3_ which turned out to be a good candidate for illustrating how the geometries evolve under finite electrode potential.

Figures 6b and 2 show that the effect of the solvent does not influence the structural properties significantly, i.e., at PZC, the structure was not modified considerably. On the other hand, the negative and positive potentials deform the geometries. In the oxidative direction (specifically, *U* – *U*_SHE_ = 1.0 V ⇔ *N* – *N*_0_ < 0, as the potential is higher than *U*_PZC_), the decrease of *N* leads to the noticeable modification of the cluster’s shape. This can be interpreted by the oxidation of the copper atoms, and weakening of the copper–copper binding may lead to cluster dissociation. The reductive case (*U* – *U*_SHE_ = −1.0 V) is more interesting due to its relevance in electrochemical catalytic processes.^76,77^ Here, the positions of the copper atoms were modified to a much lesser extent compared with the previous case. The maximum deviation of the copper nuclei position from the original values was *d* = 0.41 Å (for the copper, located furthest from the substrate). This indicates stronger binding between the copper atoms, in line with the experimentally observed spontaneous and reversible aggregation–dissociation of coppers at reductive or oxidative potentials, respectively.^5,78^

We also examined how the three descriptors, defined in eqs 2–4, change when N_4_V_2_–Cu_3_ is reoptimized under electrochemical conditions. The interaction energy was not investigated in the remaining part of the paper since the definition of the total energy of free clusters is elusive. Among the descriptors, gained energy was found to have the maximum energy difference, Δ*E* = 0.065 eV between fixed and reoptimized structures, in the case of *U* – *U*_SHE_ = −1.0 V electrode potential. Based on these, we selected to study the stability and electronic structure of models N_4_V_2_–Cu_*n*_ (*n* = 1–5) with no reoptimization and at reductive electrode potentials.

**Figure 7:**
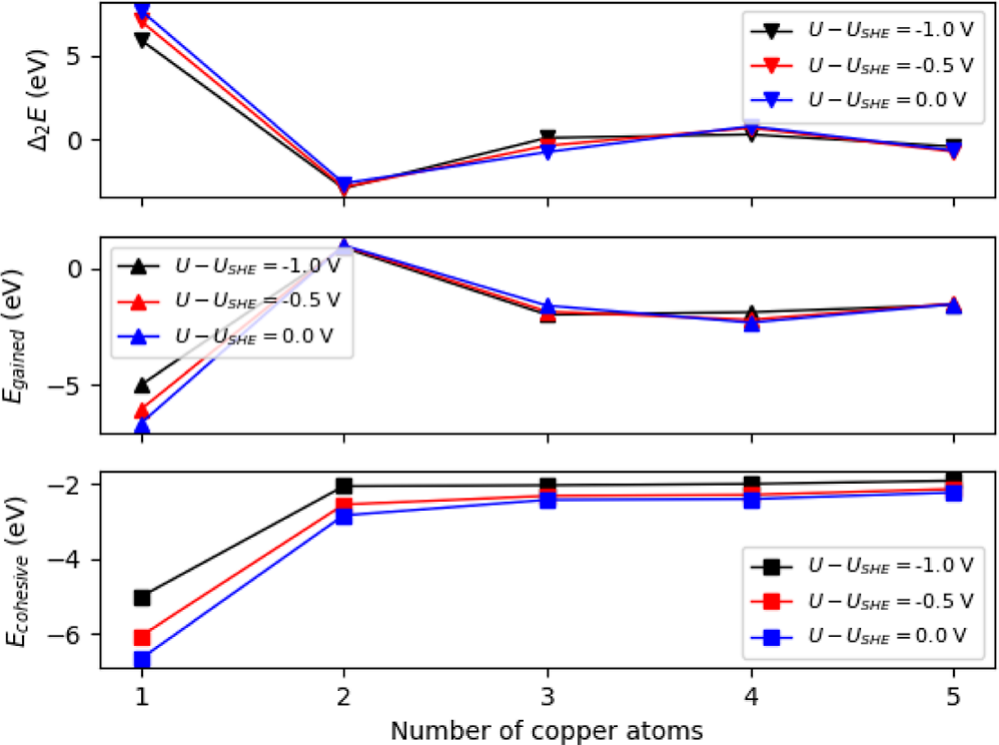
Stability descriptors of N_4_V_2_–Cu_*n*_ (*n* = 1–5) at electrode potentials *U* – *U*_SHE_ = 0.0, −0.5 and −1.0 V.

We also investigated the voltage dependence of the energetic stability descriptors. The free energies in the spin-polarized and unpolarized cases showed minor differences. Based on that, here, we discuss computations containing no spin-polarization. The dependence of the descriptors on the cluster’s size and the electrode potential is depicted on Figure 7.

The descriptors are shown at three different negative potentials. Compared to the stability of the models, studied under vacuum conditions (see Figure 3), there is no dramatic change in the energy terms. The values at *U* – *U*_SHE_ = 0.0 and −0.5 V are relatively close, while at *U* – *U*_SHE_ = −1.0 V, N_4_V_2_–Cu_3_ becomes more stable than N_4_V_2_–Cu_4_, as shown by the second order interaction and the gained energies. The remarkable difference between the two negative potential cases can be understood by comparing the PZC values of the structures, listed in Table 1, and the values of applied potentials. As Table 1 shows, all PZC is between −0.5 and −1.0 V, i.e., for −1.0 V extra electrons are injected into the system. In conclusion, the reductive electrode potential induces the decrease of the N_4_V_2_–Cu_4_ structure’s stability resulting in the dominance of the Cu_3_.

### 3.4 Electronic Structure under Electrochemical Conditions

**Figure 8:**
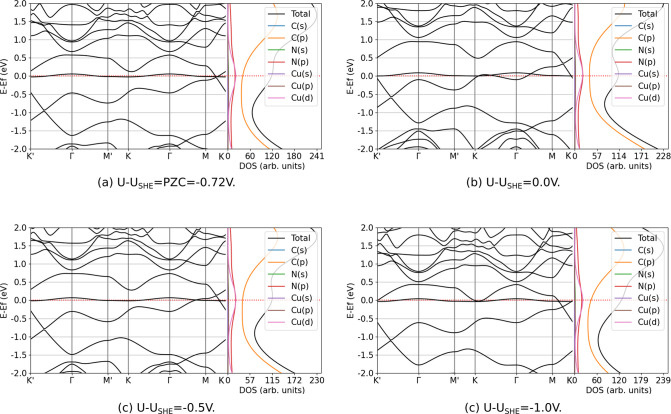
Band structure and total and orbital projected DOS of N_4_V_2_–Cu at potentials of *U* – *U*_SHE_ = PZC = −0.72, 0.0, −0.5, and −1.0 V.

The band structure and DOS of N_4_V_2_–Cu_*n*_ (*n* = 1–5) were studied to examine the effect of the electrochemical environment (presence of electrolyte and finite electrode potential). Here, the point group symmetries of the given model were supposed to be the same as discussed in Section 3.2 since the previously optimized geometries were investigated (justified in Section 3.3) and we performed computations without spin-polarization, as mentioned in Section 3.3. Our computations show that the effect of the electrolyte is very small compared to other factors, discussed below. In the followings, we focused on how the electronic structures evolve (compared to the PZC case) when electrode potential is applied. Similar trends characterize the models; hence, only one illustrative case is presented and discussed here: the N_4_V_2_–Cu. The band structure and DOS at electrode potentials *U* – *U*_SHE_ = PZC = −0.72, 0.0, −0.5, and −1.0 V are illustrated on Figure 8.

The change of the applied potential compared to PZC will lead to electron gain or loss, and the Fermi-level shifts toward the conduction or valence bands, respectively. This phenomenon is widely used in graphene-based devices, where the type and density of charge carriers can be tuned in this manner.^79−81^ This concept is rather a rigid band model, meaning that there is no relaxation of electronic states, when the number of charge carriers is adjusted. The case of N_4_V_2_–Cu shows a different scenario, as Figure 8 presents. The defect state formed by the hybridization of the valence p- and d-orbitals of nitrogen and copper atoms, respectively, is relocated when the Fermi-level is shifted. The N_4_V_2_–Cu_*n*_ (*n* = 2–5) models behave analogously, meaning that the bands near the Fermi-level are deformed and lifted by the potential. These bands are always formed in part by the hybridization of copper and nitrogen atomic orbitals, indicating that the change in the number of electrons (electrode potential) significantly influences them. This is consistent with the charge transfer studied under vacuum condition. In summary, the effect of the electrode potential shifts the Fermi-level, where its direction depends on the relation between the potential and the PZC of the model. Hereby, the number of charge carriers can be tuned, which is a promising feature for electrocatalysts or electrochemical sensor material design. On the other hand, N_4_V_2_–Cu demonstrated that due to the potential (i.e., charge carrier is removed from or added to the system), the bands having high contributions from the orbitals of copper atoms are deformed.

## 4 Conclusions

We have compared the stability of N_4_V_2_–Cu_*n*_ (*n* = 2–5) under vacuum condition and showed the highest stability of Cu_3_ and Cu_4_ among the clusters using interaction energy descriptors; thus, they are promising catalytic/sensor active material. The analysis of the electronic structures indicated that graphene remains semimetal when the pyridine-type defect is presented, while a semimetal–metal and a topologically nontrivial–trivial phase transition occur, when clusters are deposited. This supports to predict the qualitative behavior of the materials in electron transport, which is essential when they are applied as, e.g., electrodes in electrochemistry. We also performed computations using implicit solvent model and utilized the GCP-K method to investigate the cluster decorated, nitrogen-doped graphene structures under electrochemical conditions. Thereby, we studied the models close to such conditions which significantly affect the electrochemical processes. We found that Cu_3_ becomes slightly more stable than Cu_4_ at *U* – *U*_SHE_ = −1.0 V. We also showed that the band structures remain rigid, when finite electrode potential is applied, only in the case of the pristine N_4_V_2_, while the bands change significantly for N_4_V_2_–Cu_*n*_. The potential affects mostly the states formed by nitrogen and copper atoms, leading to the deformation and relaxation of these bands and to the lift in their energy. This affects the electronic and transport properties of the materials. According to the best of our knowledge, this careful computational study of nitrogen-doped, defected graphene-supported copper clusters under both vacuum and electrochemical conditions is unique. The results help to have a deeper understanding about the binding of copper clusters to this substrate and the conductivity of the N_4_V_2_–Cu_*n*_ (*n* = 1–5) models, which is crucial in the material development of efficient electrocatalysts and sensors.

## Data Availability

All structures calculated in this work have been uploaded to the ZENODO repository at http://10.5281/zenodo.8272188.

## Supporting Information Available

Different configurations with their total energy; modifications in the previously optimized structures at different electrode potentials, deviations, and differences in coordinates and energy descriptors; test computations on the size of the supercell, the cutoff energy of the PW basis, and the sufficient number of *k*-points; test computations on the size of the supercell and the sufficient number of *k*-points; calculated charge transfer of N_4_V_2_ and Cu_*n*_ (*n* = 1–5) clusters and the map of charge transfer on the atomic grid; study of the effect of the size of the LCAO and PW bases and the resolution of grid spacing on the stability descriptors; detailed mapping and discussion of band structures; summary of the formalism and the study of the applicability of GCP-K theory; details about the relation between electronic charge and electrode potential, computed and predicted chemical potential, free energy of the structures, and fitting parameters; detailed discussion of the computational process of *G*(*U*) and the stability descriptors; comparison of the energetics and the electronic structure properties of polarized and unpolarized ground states and computation of total and local magnetic moments; discussion of the free energy difference for N_4_V_2_–Cu derived from polarized and unpolarized computations at different electrode potentials; comparison of electronic structure properties at different conditions (vacuum and electrochemical environment); analysis of band structures and DOS; electronic structure properties at different electrode potentials; and analysis of band structures and DOS (PDF)
