# Supplementary material for: Stability and Electronic Structure of Nitrogen-Doped Graphene-Supported Cun (n = 1–5) Clusters in Vacuum and under Electrochemical Conditions: Toward Sensor and Catalyst Design
Source: J Phys Chem C Nanomater Interfaces. 2024 Mar 8;128(11):4677–86. doi: 10.1021/acs.jpcc.3c06475 (PMC10961840; doi:10.1021/acs.jpcc.3c06475)
Supplement: Supplementary file 1 — jp3c06475_si_001.pdf [file jp3c06475_si_001.pdf]

Supporting Information

Stability and Electronic Structure of nitrogen-doped graphene-supported Cu_n $(n = 1 - 5)$ clusters in Vacuum and under Electrochemical Conditions: towards sensor and catalyst design

Márton Guba[1] and Tibor Höltzl[2]

[1,2]Budapest University of Technology and Economics, Department of Inorganic and Analytical Chemistry , and HUN-REN-BME Computation Driven Chemistry Research group, , Szent Gellért tér 4, H-1111 Budapest, Hungary

[2]Furukawa Electric Institute of Technology, Nanomaterials Science Group, Késmárk utca 28/A, H-1158 Budapest, Hungary

Contents

1 Geometry optimizations **1**

1.1 Systems in vacuum 1

1.2 Electrochemical environment 5

2 Parameter tests **9**

2.1 Computations of surfaces under vacuum conditions 9

2.2 Computations under electrochemical conditions 14

3 Bader charge computation in vacuum **15**

4 Method testing and validation **17**

4.1 Stability tests of models simulated under vacuum conditions 17

4.2 Band structure analysis of systems studied under vacuum conditions 27

4.3 Applicability of GCP-K theory 40

4.4 Computation process of the grand canonical potential 46

5 Notes on spin-polarization **51**

5.1 Systems in vacuum 51

5.2 Electrochemical environment 59

6 Comparison of electronic structure properties in vacuum and in an electrochemical environment **61**

7 Electronic structure properties of the studied models at different electrode potentials **64**

1 Geometry optimizations

1.1 Systems in vacuum

We reoptimized the previously reported ground state structures of Cu_n clusters ($n = 2-5$) [1, 2, 3] and that of N_4V_2 [4, 5, 6] using the linear combination of atomic orbitals (LCAO), triple-zeta polarized (TZP) basis and the PBE exchange-correlation functional [7], including D3 [8] dispersion correction and the FIRE optimization algorithm [9]. All forces were relaxed to be less than 0.01 eV/Å. The Monkhorst-Pack [10] k-point mesh was selected to be $(4, 4, 1) + \Gamma$ for optimizations (see Section 2.1). The relaxed structures are presented on Figures S1-S2.

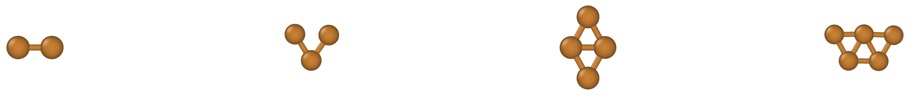

(a) Cu_2. (b) Cu_3. (c) Cu_4. (d) Cu_5.

Fig. S1: Ground state structure of copper clusters with $n = 2-5$ atoms.

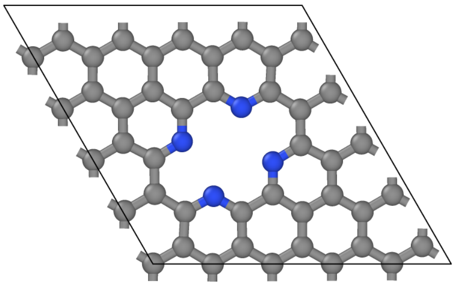

Fig. S2: Optimized geometry of N_4V_2. The simulation cell in the periodic directions is also shown.

We carried out initial optimizations on the N_4V_2-Cu_n ($n = 2-5$) models under vacuum conditions using the same method and settings as it was described above. The initial geometries and the result of the relaxations are given in Figures S3-S11.

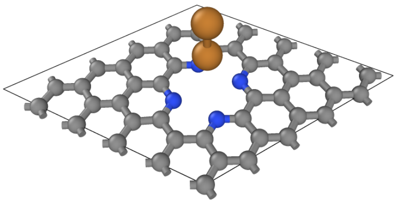

(a) Initial structure.

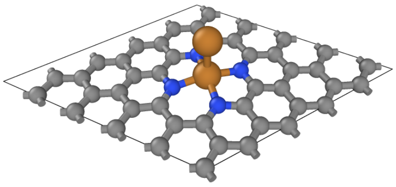

(b) Final structure (denoted as #1).

Fig. S3: Geometries showing the optimization of N_4V_2 - Cu_2. The simulation cell in the periodic directions is also shown.

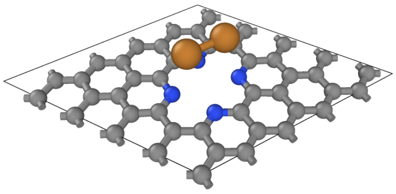

(a) Initial structure.

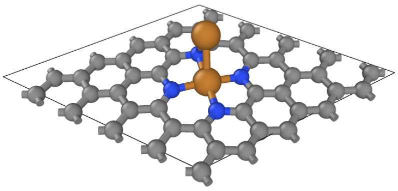

(b) Final structure (denoted as #2).

Fig. S4: Geometries showing the optimization of N_4V_2 - Cu_2. The simulation cell in the periodic directions is also shown.

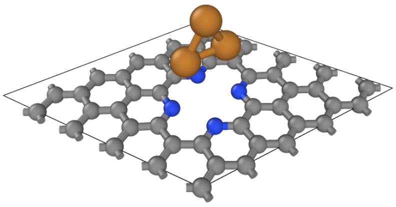

(a) Initial structure.

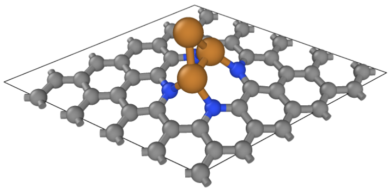

(b) Final structure (denoted as #1).

Fig. S5: Geometries showing the optimization of N_4V_2 - Cu_3. The simulation cell in the periodic directions is also shown.

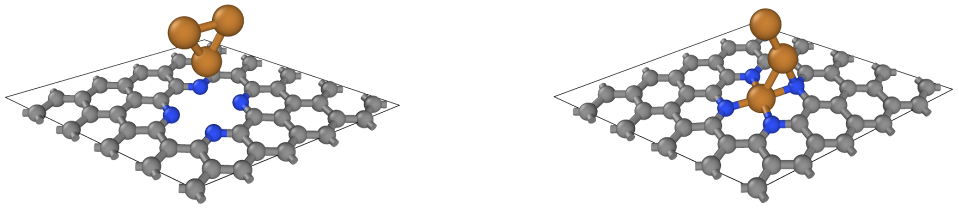

(a) Initial structure. (b) Final structure (denoted as #2).

Fig. S6: Geometries showing the optimization of N_4V_2 - Cu_3. The simulation cell in the periodic directions is also shown.

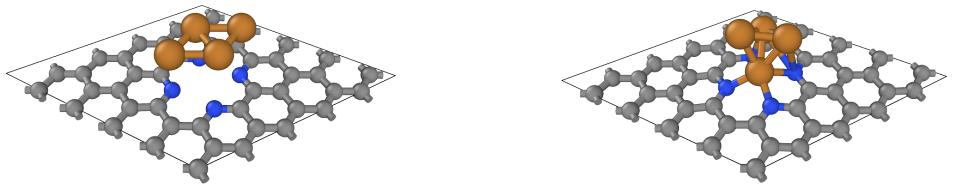

(a) Initial structure. (b) Final structure (denoted as #1).

Fig. S7: Geometries showing the optimization of N_4V_2 - Cu_4. The simulation cell in the periodic directions is also shown.

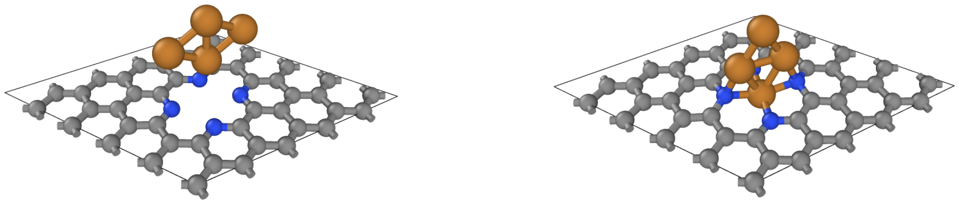

(a) Initial structure. (b) Final structure (denoted as #2).

Fig. S8: Geometries showing the optimization of N_4V_2 - Cu_4. The simulation cell in the periodic directions is also shown.

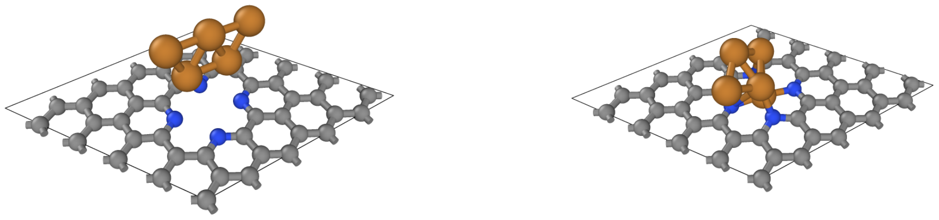

(a) Initial structure. (b) Final structure (denoted as #1).

Fig. S9: Geometries showing the optimization of N_4V_2 - Cu_5. The simulation cell in the periodic directions is also shown.

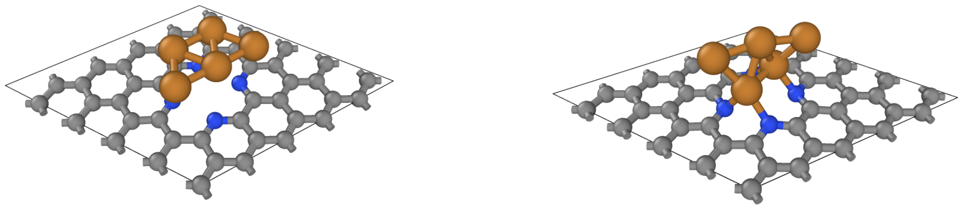

(a) Initial structure. (b) Final structure (denoted as #2).

Fig. S10: Geometries showing the optimization of N_4V_2 - Cu_5. The simulation cell in the periodic directions is also shown.

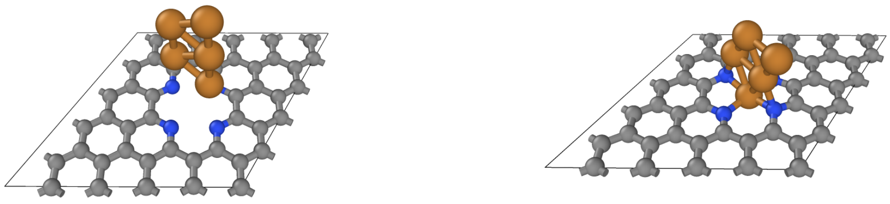

(a) Initial structure. (b) Final structure (denoted as #3).

Fig. S11: Geometries showing the optimization of N_4V_2 - Cu_5. The simulation cell in the periodic directions is also shown.

We found that the optimization of initial configuration of N_4V_2-Cu_3 with cluster in parallel orientation with the surface also leads to structure #2 (see Figure S6). Similarly,

in case of the model containing Cu_4, structure #2 (see Figure S8) can be obtained from another perpendicular initial geometry, where the cluster is rotated by $\pi/2$ around its centre. Finally, for the N_4V_2-Cu_5, the relaxation of the parallel initial configuration may result (using different optimization algorithms) in structure #1 (see Figures S9-S10). The total energy of the relaxed geometries is listed in Table S1.

Tab. S1: The total energies of the configurations after relaxation.

**Geometry**	**Total energy of structure #1 (eV)**	**Total energy of structure #2 (eV)**	**Total energy of structure #3 (eV)**
N_4V_2-Cu_2	-428.09	-428.19	-
N_4V_2-Cu_3	-430.08	-430.83	-
N_4V_2-Cu_4	-432.10	-433.12	-
N_4V_2-Cu_5	-433.82	-434.50	-434.83

The lowest energy configurations, i.e., #2 in case of N_4V_2-Cu_2, N_4V_2-Cu_3 and N_4V_2-Cu_4 were selected for final optimization. Structure #1 and #3 containing Cu_5 turned out to by quasi-degenerate (only 0.01 eV difference), both were investigated in final relaxation. This showed that geometry #3 has lower total energy with $\Delta E \approx 0.65$ eV. After choosing the suitable parameters (see Section 2), the most stable versions were further optimized. The ground state structures are visualized on Figure 1 of the Main Text, and all the coordinates are available at reference [11], in .xyz format.

1.2 Electrochemical environment

We were interested in, how the geometries of N_4V_2-Cu_n ($n = 2-5$) studied under vacuum conditions are affected by the presence of the electrolyte and the electrode potential. N_4V_2 and N_4V_2-Cu were turned to be outstandingly stable (no significant modification in geometry and energy), that we dispense from the discussion of these structures. N_4V_2-Cu_n ($n = 2-5$) were reoptimized (starting from the geometries, already optimized in vacuum) at potentials $U - U_{SHE} = -1.0$ V (reductive), PZC (neutral) and 1.0 V (oxidative). The relaxed geometries are depicted on Figures S12-S15.

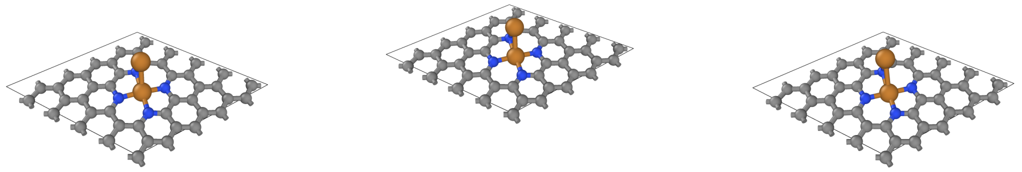

(a) $U - U_{SHE} = -1.0$ V. (b) $U - U_{SHE} = PZC = -0.86$ V. (c) $U - U_{SHE} = 1.0$ V.

Fig. S12: The geometry of N_4V_2-Cu_2 after relaxation at $U - U_{SHE} = \pm 1.0$ and $U - U_{SHE} = PZC = -0.86$ V electrode potentials. The simulation cell in the periodic directions is also shown.

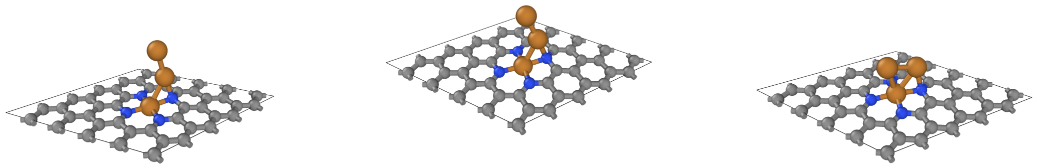

(a) $U - U_{SHE} = -1.0$ V. (b) $U - U_{SHE} = PZC = -0.69$ V. (c) $U - U_{SHE} = 1.0$ V.

Fig. S13: The geometry of N_4V_2-Cu_3 after relaxation at $U - U_{SHE} = \pm 1.0$ and $U - U_{SHE} = PZC = -0.69$ V electrode potentials. The simulation cell in the periodic directions is also shown.

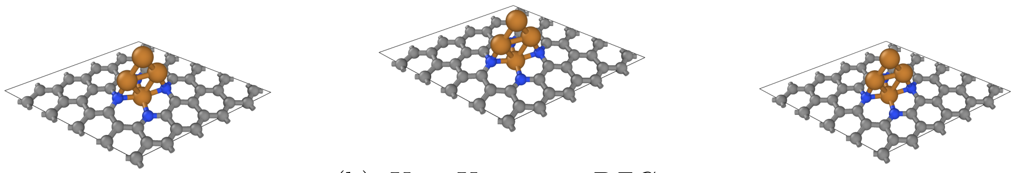

(a) $U - U_{SHE} = -1.0$ V. (b) $U - U_{SHE} = PZC = -0.97$ V. (c) $U - U_{SHE} = 1.0$ V.

Fig. S14: The geometry of N_4V_2-Cu_4 after relaxation at $U - U_{SHE} = \pm 1.0$ and $U - U_{SHE} = PZC = -0.97$ V electrode potentials. The simulation cell in the periodic directions is also shown.

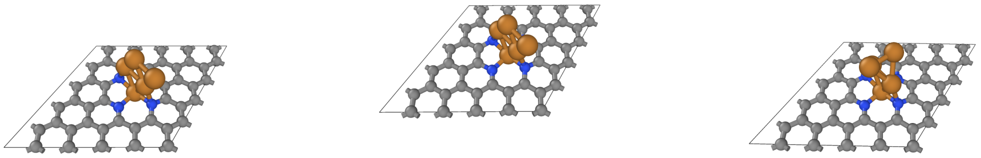

(a) $U - U_{SHE} = -1.0$ V.

(b) $U - U_{SHE} = PZC = -0.83$ V.

(c) $U - U_{SHE} = 1.0$ V.

Fig. S15: The geometry of N_4V_2-Cu_5 after relaxation at $U - U_{SHE} = \pm 1.0$ and $U - U_{SHE} = PZC = -0.83$ V electrode potentials. The simulation cell in the periodic directions is also shown.

The reoptimized geometries show similar trends for each structure: the reductive and PZC cases look unmodified, while oxidative potential causes noticeable change in geometry. All coordinates are listed in reference [11], in .xyz format. For the sake of quantified data, the deviances in chemical potential from the theoretical value (see Section 4.3), in free energy (between rigid and reoptimized structures) and deviations in spatial coordinates from the rigid structures (no reoptimization) were studied. Here, only potentials $U - U_{SHE} = \pm 1.0$ V were examined, since at PZC, the difference between reoptimized and rigid cases was negligible (1-2 orders of magnitude smaller) compared to the former two. The values are tabulated into Table S2.

Tab. S2: The absolute differences in chemical potential from the theoretical values, in free energy (between rigid and reoptimized structures) and deviations in spatial coordinates from the rigid structures (no reoptimization) of N_4V_2-Cu_n ($n = 2-5$), for electrode potentials $U - U_{SHE} = \pm 1.0$ V.

**Structure**	$U - U_{SHE}$ **(V)**	**Absolute difference in chemical potential (eV)**	**Absolute difference in free energy (eV)**	**Maximum deviation in spatial coordinates (Å)**
N_4V_2 - Cu_2	-1.0	0.140	0.006	0.070
N_4V_2 - Cu_2	1.0	1.045	0.136	0.412
N_4V_2 - Cu_3	-1.0	0.092	0.012	0.409
N_4V_2 - Cu_3	1.0	0.029	0.163	1.961
N_4V_2 - Cu_4	-1.0	0.144	0.016	0.072
N_4V_2 - Cu_4	1.0	0.400	0.114	0.127
N_4V_2 - Cu_5	-1.0	0.083	0.009	0.057
N_4V_2 - Cu_5	1.0	0.320	0.808	2.483

Considering the oxidative direction, the values listed in Table S2, are consistent with the results of the reoptimizations: significant modification can be observed for each model. It is not surprising, since oxidation means removing electrons from the system, leading to the destabilization of the given structure. In the Main Text we focused mainly on the reductive direction (see Sec. 3.3 of the Main Text) and found that the coordinates of N_4V_2-Cu_3 had been modified at most by 0.41 Å. For the sake of completeness, we studied the deviance in stability descriptors (for definition see Equations (2), (3) and (4) in the Main Text) caused by the reoptimization for model N_4V_2-Cu_3, at potentials $U - U_{SHE} = -1.0$-0.0 V. The detailed description of the calculation process is discussed in Section 4.4. In Table S3, we list only the results about the difference in cohesive, gained and second order energies between rigid (no reoptimization) and reoptimized cases.

Tab. S3: The absolute difference in cohesive, gained and second order energies of N_4V_2-Cu_3 between rigid (no reoptimization) and reoptimized cases for electrode potentials $U - U_{SHE} = -1.0$-0.0 V.

$U - U_{SHE}$ **(V)**	**Absolute difference in cohesive energy (eV)**	**Absolute difference in gained energy (eV)**	**Absolute difference in second order energy (eV)**
-1.0	0.000051	0.000046	0.000091
-0.9	0.000036	0.000109	0.000217
-0.8	0.000055	0.000165	0.000330
-0.7	0.000071	0.000214	0.000429
-0.6	0.000086	0.000257	0.000514
-0.5	0.000098	0.000293	0.000586
-0.4	0.000107	0.000322	0.000645
-0.3	0.000115	0.000345	0.000690
-0.2	0.000120	0.000361	0.000721
-0.1	0.000123	0.000370	0.000739
0.0	0.000124	0.000372	0.000744

The values in Table S3 clearly show that the difference in energy terms converges faster than the total energies. Based on these experiences, we studied the stability and electronic structure of the rigid models at reductive electrode potentials (see Sec. 3.3 of the Main Text).

2 Parameter tests

2.1 Computations of surfaces under vacuum conditions

The vacuum applied in the non-periodic directions, the density of the k-point mesh and the cut-off energy (frequency) of plane wave (PW) basis were tested. The obtained parameters were determined for representative model systems and used in the final geometry optimizations, stability calculations and electronic structure analysis. The representatives were Cu_5 (see Figure S1) for clusters, N_4V_2 (see Figure S2) and N_4V_2 - Cu_5 (see the relaxed version on Figure S11) in case of hybrid systems. The proper size of the vacuum (in the non-periodic direction) and the cut-off energy depend mainly on the real space extension of the simulated system, thus it makes sense to test them on the largest models.

The density of the k-point mesh is relevant only in periodic directions, where in general, the larger the system, the less k-points are needed for precise computations. The total energies with respect to the discussed quantities are depicted on Figures S16-S17 for cluster Cu_5, furthermore on Figures S18-S20 for N_4V_2 and on Figures S21-S23 for N_4V_2-Cu_5.

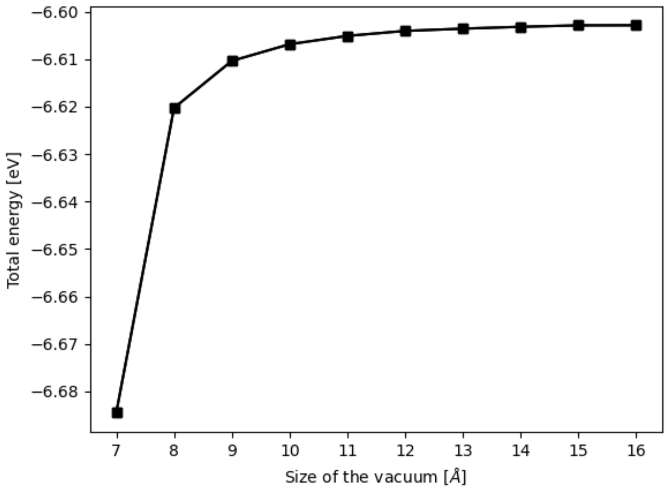

Fig. S16: Total energy of Cu_5 cluster for different size of the applied vacuum.

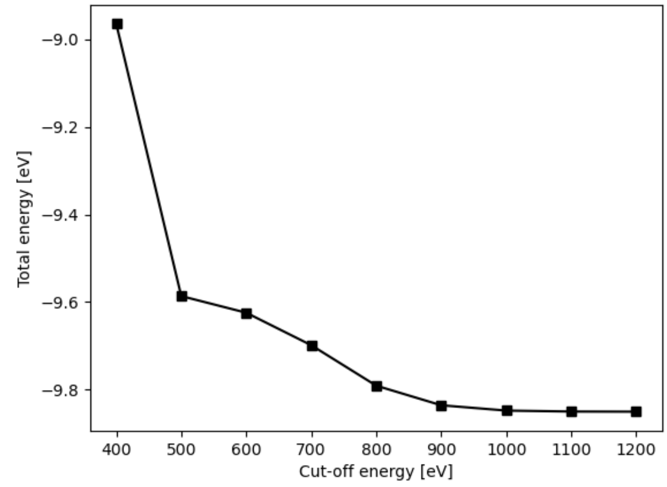

Fig. S17: Total energy of Cu_5 cluster as the function of PW cut-off energy.

The size of the k-point scheme was not tested for clusters, where the real-space extension of electronic states is supposed to be finite, hence the origin of the first Brillouin-zone (point Γ) should be sufficient to be sampled.

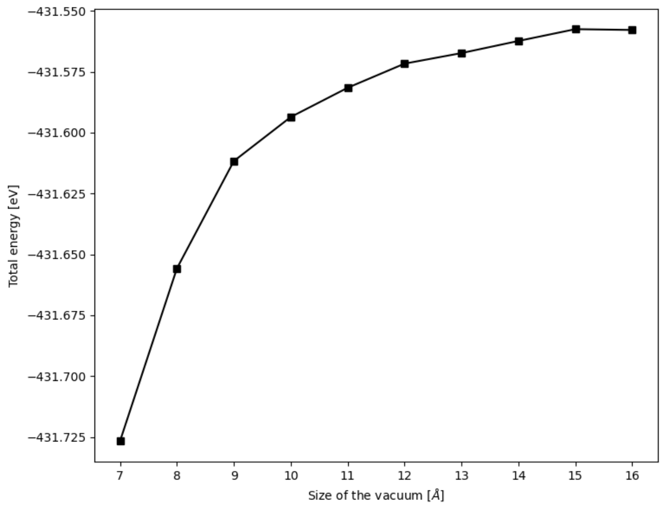

Fig. S18: Total energy of N_4V_2 for different size of the applied vacuum.

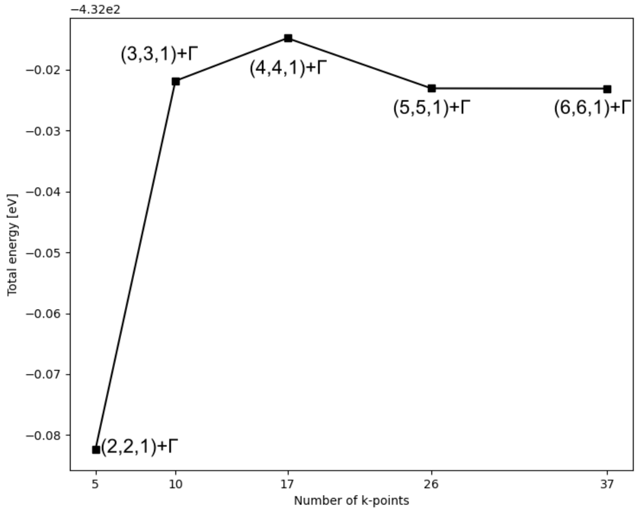

Fig. S19: Total energy of N_4V_2 in case of different number of k-point (for sampling the Brillouin-zone).

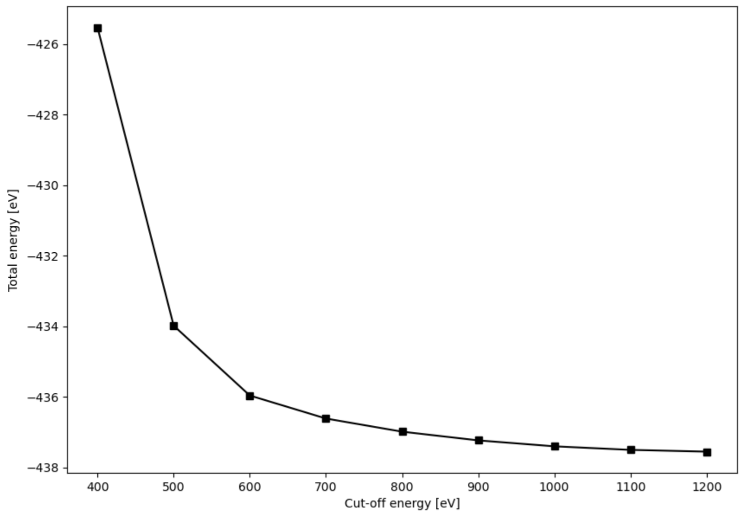

Fig. S20: Total energy of N_4V_2 as the function of PW cut-off energy.

The k-point scheme is usually abbreviated in the form of (n, m, l), where integers n, m, l denote the number of sampling k-points in the directions of momentum vectors k_x, k_y, k_z, respectively. The relevant figures present the total energy as the function of the total number of k-points, which is given by the product of the three numbers. In our case, only directions x and y were periodic, therefore we used the version of $(n, m, 1)$ and also included point Γ (which is not by default).

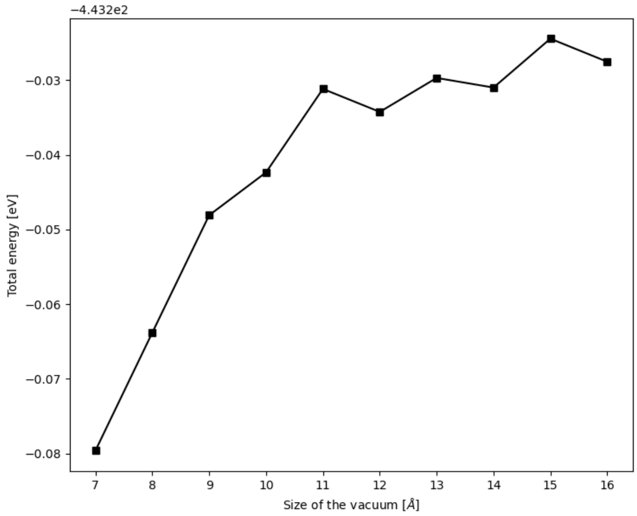

Fig. S21: Total energy of N_4V_2 - Cu_5 for different size of the applied vacuum.

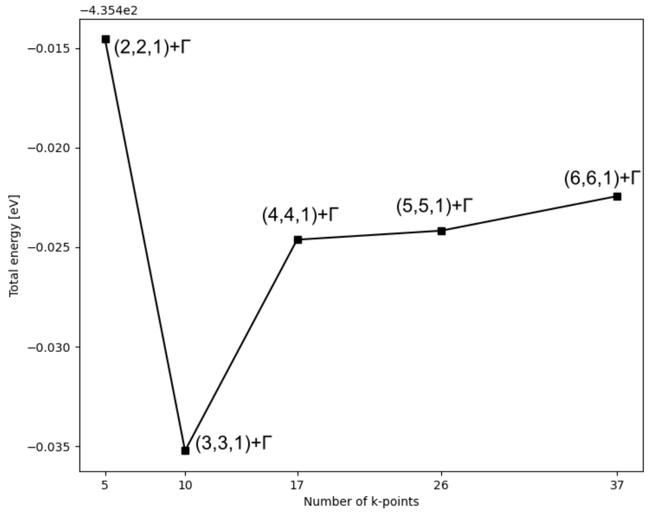

Fig. S22: Total energy of N_4V_2 - Cu_5 in case of different number of k-point (for sampling the Brillouin-zone).

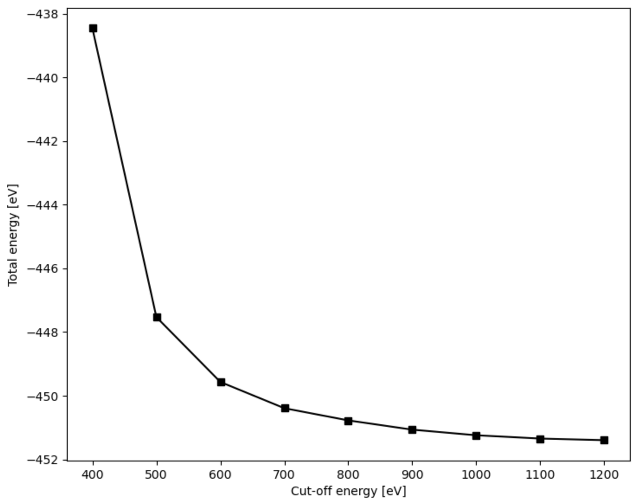

Fig. S23: Total energy of N_4V_2 - Cu_5 as the function of PW cut-off energy.

Figure S21 presents "zig-zag" like curve, which occurs probably due to "eggbox error" [12], i.e., because of the finite density of the real space integration grid. This can be improved by applying finer real-space grid resolution (see Section 4.1). Based on the results of convergence tests, deviance in total energy within 0.01 eV was found to be an appropriate criterion. The comparison of Figures S16, S18 and S21 showed that vacuum of 12 Å leads to sufficiently converged results in all cases. The proper number of k-

points was selected by analysing and confronting the total energies, plotted on Figures S19 and S22. Here, energies converged reasonably well, hence we decided to use the sampling scheme $(4, 4, 1)$ + point Γ. The correct value for PW cut-off turned out to be non-trivial to determine, as Figures S17, S20 and S23 show. While, for cluster Cu_5 total energy converges sufficiently quickly (after around 1000 eV), the larger systems (see Figures S20 and S23) seem to need larger value (at least for 0.01 eV tolerance value). Due to the highly increased computational resource demand, we decided to apply PW basis with cut-off energy 1000 eV for the simulation of each model, where the total energies converged within 0.1 eV.

2.2 Computations under electrochemical conditions

Similarly to the previous cases (Section 2.1), electrochemical simulations also required a proper parameter set. The size of the cell (i.e., size of the vacuum) and the number of k-points were estimated (we used the default value, 544 eV applied in the jDFTx program [13] for cut-off energy). Here, the N_4V_2 - Cu_5, presented on Figure S11 (configuration #3), was studied, since the parameters depend mainly on spatial extension, i.e. if the largest system converges, the smaller ones will do as well. The results of the convergence calculations are demonstrated on Figures S24 and S25.

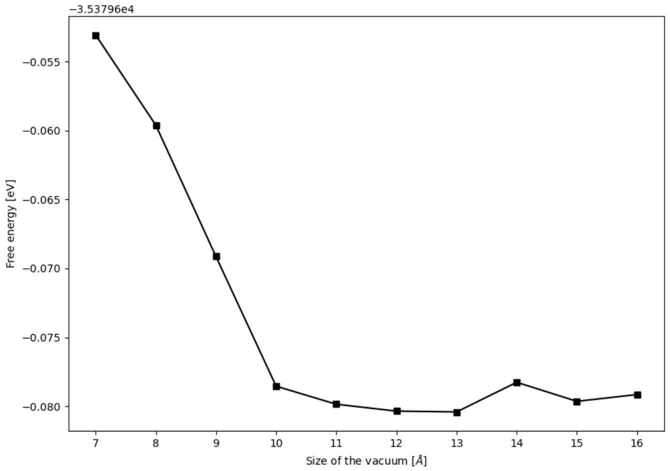

Fig. S24: Total energy of N_4V_2-Cu_5 surrounded by electrolyte with respect to the vacuum.

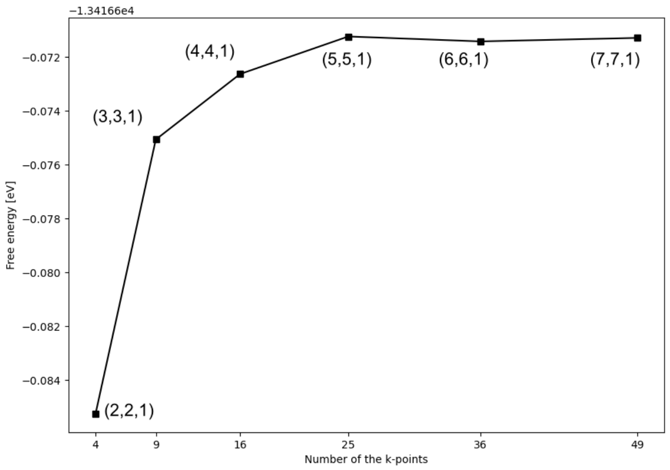

Fig. S25: Total energy of N_4V_2-Cu_5 with respect to the number of k-points in the first Brillouin zone.

By following the method applied in Section 2.1, we found that here 12 Å is also sufficient in the non-periodic directions, while k-point scheme $(5, 5, 1)$ was needed to satisfy the usual convergence criterion (deviance within 0.01 eV).

3 Bader charge computation in vacuum

More details about the interaction between the clusters and N_4V_2 can be obtained by analysing the charge transfer between them. Here, Bader atomic charges [14, 15, 16] were computed using the code described in reference [17]. Then, these were summed up for atoms belonging to the cluster or the N_4V_2 moiety. The cluster size-dependent charge transfer is presented in Figure S26, while the atomic charges are depicted in Figure S27.

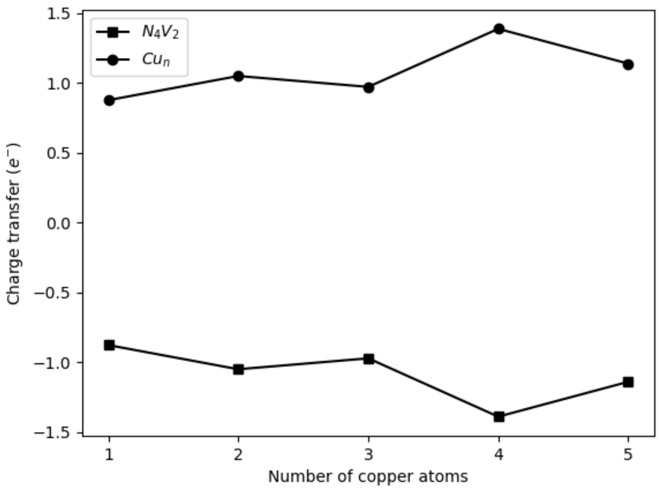

Fig. S26: Charge transfer in N_4V_2-Cu_n ($n = 1 - 5$) as the function of the size of the cluster.

The significant electron transfer from the clusters to N_4V_2 is well visible in each case. The largest charge transfer is observed for the surface bound Cu_4 cluster, consistently with its strongest binding to N_4V_2 (see Sec 3.1 of the Main Text).

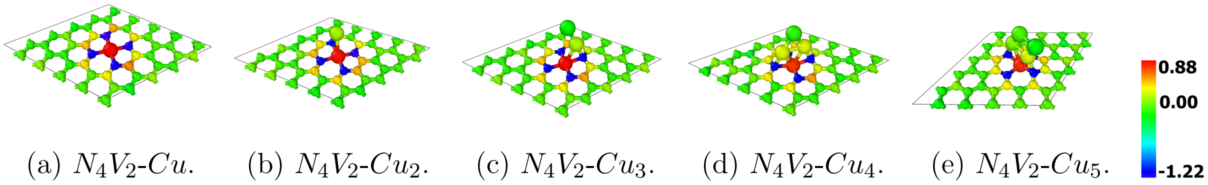

(a) N_4V_2-Cu. (b) N_4V_2-Cu_2. (c) N_4V_2-Cu_3. (d) N_4V_2-Cu_4. (e) N_4V_2-Cu_5.

Fig. S27: Bader atomic charges of N_4V_2-Cu_n ($n = 1 - 5$). The colour scale (in the unit of e^-) is shown on the figure. For atomic symbols, see Figure 2 in the Main Text.

As nitrogen has the highest electronegativity among the constituent atoms, these withdraw electrons not only from coppers but also from the adjacent carbons, which is clearly shown on Figure S27. On the other hand, the charge of the copper atom with the highest coordination to the nitrogens, is near to +1. The charges help to interpret the stability trends. We already showed maximally coordinated coppers to the nitrogen atoms in these cases in Sec. 3.1 of the Main Text, thus N_4V_2-Cu_4 can benefit both from the largest charge transfer and the high number of copper-nitrogen bonds. On the other hand, the further increase of the number of the copper atoms does not lead to an increased stability, as shown by the decreased average coordination of the coppers to nitrogens (Figure 2 in the Main Text), the weak cohesion between the copper atoms

(Figure 3 in the Main Text) and the lower charge transfer (Figure S26) for N_4V_2-Cu_5.

4 Method testing and validation

4.1 Stability tests of models simulated under vacuum conditions

There are several settings in GPAW computations (e.g. size of the basis, resolution of real space grid etc.), which may influence the accuracy of stability computations. Here we list the results of those calculations briefly, which were performed to investigate the effect of various parameters. The interaction, gained and cohesive energies (see Equations (1), (3) and (4) in Main Text) were computed and tabulated into Tables S4, S5 and S6, respectively.

Tab. S4: The interaction energy E_{int} as the function of the number of copper atoms n, calculated using different settings.

**Settings of the calculation**	$E_{int}(n = 2)$ **(eV)**	$E_{int}(n = 3)$ **(eV)**	$E_{int}(n = 4)$ **(eV)**	$E_{int}(n = 5)$ **(eV)**
**PW, h=0.1, PBE**	-3.18336	-4.05818	-3.74194	-3.51052
**PW, h=0.2, PBE**	-3.18438	-4.05803	-3.74192	-3.51024
**tzp, small, h=0.1 PBE**	-3.96533	-5.0323	-4.84059	-4.49035
**tzp, small, h=0.2, PBE**	-3.83705	-4.7713	-4.64421	-4.52323
**tzp, large, h=0.1, PBE**	-3.8209	-4.56207	-4.37647	-4.12422
**tzp, large, h=0.2, PBE**	-3.82209	-4.33724	-4.18278	-4.17271
**tzp, small, h=0.1, bssecomp, PBE**	-4.91676	-6.11258	-5.76609	-5.64995
**tzp, small, h=0.2, bssecomp, PBE**	-2.83253	-3.81123	-3.32228	-3.32538
**tzp, large, h=0.1, bssecomp, PBE**	-2.86922	-3.72009	-3.48572	-3.24578
**tzp, large, h=0.2, bssecomp, PBE**	-2.87023	-3.49741	-3.29229	-3.29367
**PW, h=0.1, PBED3**	-3.6236	-4.55166	-4.47968	-4.31025
**PW, h=0.2, PBED3**	-3.62463	-4.5515	-4.47966	-4.30997
**tzp, small, h=0.1, PBED3**	-4.29757	-5.30606	-5.42708	-5.18857
**tzp, small, h=0.2, PBED3**	-4.27606	-5.26356	-5.38034	-5.32087
**tzp, large, h=0.1, PBED3**	-4.25992	-5.05433	-5.1126	-4.92186
**tzp, large, h=0.2, PBED3**	-4.2611	-4.82949	-4.91891	-4.97034
**tzp, small, h=0.1, bssecomp, PBED3**	-5.35577	-6.60483	-6.50222	-6.44759
**tzp, small, h=0.2, bssecomp, PBED3**	-3.27155	-4.30349	-4.05841	-4.12302
**tzp, large, h=0.1, bssecomp, PBED3**	-3.30824	-4.21235	-4.22185	-4.04341
**tzp, large, h=0.2, bssecomp, PBED3**	-3.30924	-3.98967	-4.02842	-4.0913

Tab. S5: The gained energy E_{gained} as the function of the number of copper atoms n, calculated using different settings.

**Settings of the calculation**	$E_{gained}(n=1)$ **(eV)**	$E_{gained}(n=2)$ **(eV)**	$E_{gained}(n=3)$ **(eV)**	$E_{gained}(n=4)$ **(eV)**	$E_{gained}(n=5)$ **(eV)**
**PW, h=0.1, PBE**	-4.81269	-0.61452	-2.30233	-2.30937	-2.02999
**PW, h=0.2, PBE**	-4.81245	-0.61582	-2.30114	-2.30925	-2.02999
**tzp, small, h=0.1 PBE**	-5.0726	-0.82211	-2.40958	-2.62627	-2.1738
**tzp, small, h=0.2, PBE**	-5.07236	-0.82341	-2.40839	-2.62614	-2.1738
**tzp, large, h=0.1, PBE**	-5.58529	-0.78363	-2.69177	-2.79813	-2.22545
**tzp, large, h=0.2, PBE**	-5.30312	-1.18705	-2.7106	-2.96138	-2.57897
**tzp, small, h=0.1, bssecomp, PBE**	-5.70781	-1.19368	-3.05705	-3.31544	-2.69589
**tzp, small, h=0.2, bssecomp, PBE**	-5.27761	-1.62212	-2.83008	-3.34547	-2.93585
**tzp, large, h=0.1, bssecomp, PBE**	-5.84428	-0.99153	-2.79933	-3.11534	-2.36958
**tzp, large, h=0.2, bssecomp, PBE**	-5.56211	-1.39495	-2.81817	-3.27859	-2.72309
**PW, h=0.1, PBED3**	-5.9668	-1.40159	-3.16462	-3.63265	-2.84002
**PW, h=0.2, PBED3**	-5.5366	-1.83003	-2.93764	-3.66268	-3.07997
**tzp, small, h=0.1, PBED3**	-6.69271	-0.62768	-2.82062	-2.64336	-2.45955
**tzp, small, h=0.2, PBED3**	-4.72797	-0.75769	-2.75505	-2.59952	-2.70305
**tzp, large, h=0.1, PBED3**	-4.93776	-1.01206	-3.16675	-3.26667	-2.70819
**tzp, large, h=0.2, PBED3**	-4.47671	-1.47116	-2.94212	-3.29481	-2.94729
**tzp, small, h=0.1, bssecomp, PBED3**	-6.9517	-0.83558	-2.92819	-2.96057	-2.60368
**tzp, small, h=0.2, bssecomp, PBED3**	-4.98696	-0.96559	-2.86261	-2.91673	-2.84717
**tzp, large, h=0.1, bssecomp, PBED3**	-5.19675	-1.21996	-3.27432	-3.58388	-2.85232
**tzp, large, h=0.2, bssecomp, PBED3**	-4.7357	-1.67906	-3.04968	-3.61202	-3.09142

Tab. S6: The cohesive energy $E_{cohesive}$ as the function of the number of copper atoms n, calculated using different settings.

**Settings of the calculation**	$E_{cohesive}(n=1)$ **(eV)**	$E_{cohesive}(n=2)$ **(eV)**	$E_{cohesive}(n=3)$ **(eV)**	$E_{cohesive}(n=4)$ **(eV)**	$E_{cohesive}(n=5)$ **(eV)**
**PW, h=0.1, PBE**	-4.81269	-2.71361	-2.57651	-2.50973	-2.41378
**PW, h=0.2, PBE**	-4.81245	-2.71414	-2.57647	-2.50967	-2.41373
**tzp, small, h=0.1 PBE**	-5.0726	-2.94735	-2.7681	-2.73264	-2.62087
**tzp, small, h=0.2, PBE**	-5.07236	-2.94789	-2.76805	-2.73258	-2.62082
**tzp, large, h=0.1, PBE**	-5.58529	-3.18446	-3.02023	-2.9647	-2.81685
**tzp, large, h=0.2, PBE**	-5.30312	-3.24508	-3.06692	-3.04054	-2.94822
**tzp, small, h=0.1, bssecomp, PBE**	-5.70781	-3.45075	-3.31952	-3.3185	-3.19397
**tzp, small, h=0.2, bssecomp, PBE**	-5.27761	-3.44986	-3.24327	-3.26882	-3.20222
**tzp, large, h=0.1, bssecomp, PBE**	-5.84428	-3.41791	-3.21172	-3.18762	-3.02401
**tzp, large, h=0.2, bssecomp, PBE**	-5.56211	-3.47853	-3.25841	-3.26345	-3.15538
**PW, h=0.1, PBED3**	-5.9668	-3.68419	-3.511	-3.54141	-3.40113
**PW, h=0.2, PBED3**	-5.5366	-3.68331	-3.43475	-3.49174	-3.40938
**tzp, small, h=0.1, PBED3**	-6.69271	-3.66019	-3.38034	-3.19609	-3.04878
**tzp, small, h=0.2, PBED3**	-4.72797	-2.74283	-2.7469	-2.71005	-2.70865
**tzp, large, h=0.1, PBED3**	-4.93776	-2.97491	-3.03886	-3.09581	-3.01829
**tzp, large, h=0.2, PBED3**	-4.47671	-2.97393	-2.96333	-3.0462	-3.02642
**tzp, small, h=0.1, bssecomp, PBED3**	-6.9517	-3.89364	-3.57182	-3.41901	-3.25594
**tzp, small, h=0.2, bssecomp, PBED3**	-4.98696	-2.97627	-2.93839	-2.93297	-2.91581
**tzp, large, h=0.1, bssecomp, PBED3**	-5.19675	-3.20836	-3.23034	-3.31873	-3.22545
**tzp, large, h=0.2, bssecomp, PBED3**	-4.7357	-3.20738	-3.15481	-3.26911	-3.23357

Computations were performed with high accuracy, using PW basis with 1000 eV cut-off and PBE-D3 functional for reference values. The k-point grid was $(4, 4, 1)+\ \Gamma)$, while the resolution of the real space grid was set to be $h = 0.1$ ($125x125x100$ partition of the simulation cell). As Figure S28 shows, firstly the effect of the real-space grid was studied, where $h = 0.1$ and $h = 0.2$ ($90x90x75$ partition) cases were compared for computations on PW basis.

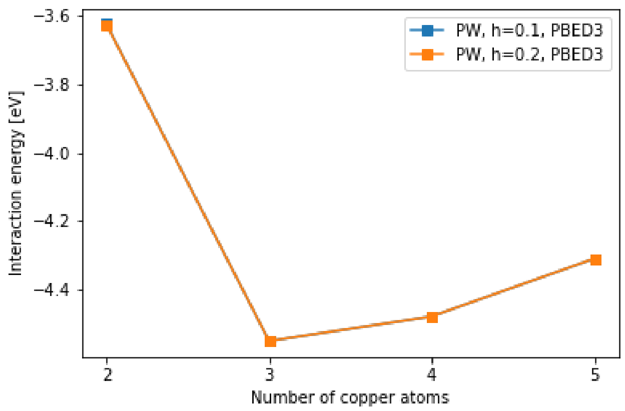

(a) Interaction energy.

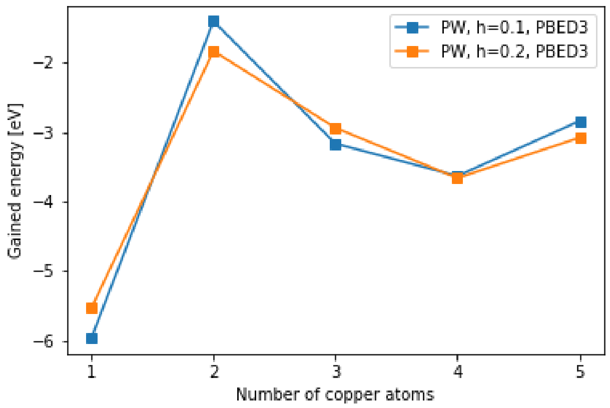

(b) Gained energy.

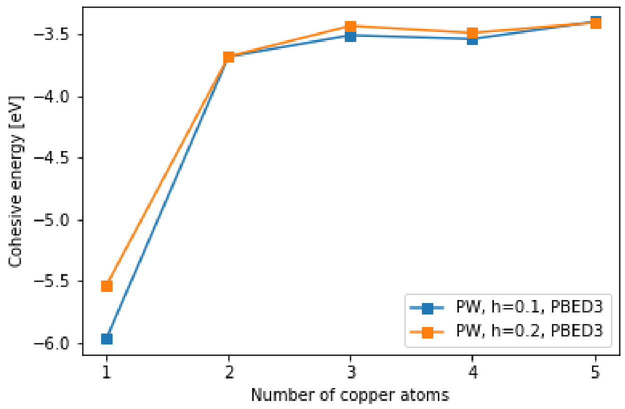

(c) Cohesive energy.

Fig. S28: Stability descriptors of the models computed on PW basis for different resolution of the real space grid.

Although, there is no significant difference (compared to other cases, max. 0.5 eV)

between values with settings $h = 0.1$ and $h = 0.2$, for the sake of accuracy (e.g., reducing eggbox error), we decided to use resolution $h = 0.1$. In the next step we examined what size of LCAO basis is needed to get results with precision close to the ones provided by PW basis. Triple-zeta polarized (TZP) basis set was used to describe the orbitals of nitrogen and carbon atoms, with three series of functions for the occupied $2s$ and $2p$ orbitals and a polarization function for the unoccupied d-orbital. The size of the total basis differed only in case of copper. Here, basis 'small' means TZP basis set with three series of functions for the $4s$ and $3d$ orbitals, furthermore p-type polarization function. While basis 'large' means three series of functions for the $4s$, $3d$ and $4p$ orbitals with f-type polarization function. The results are depicted on Figure S29.

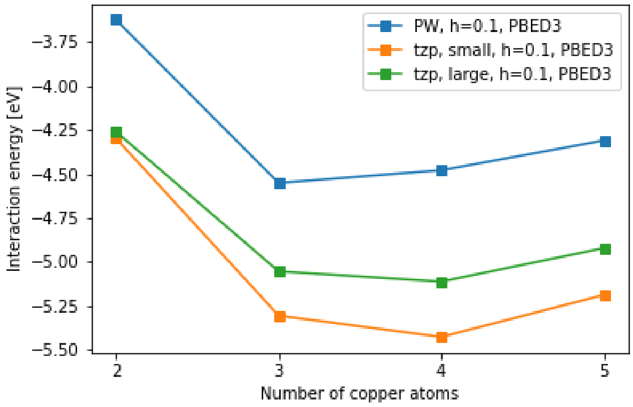

(a) Interaction energy.

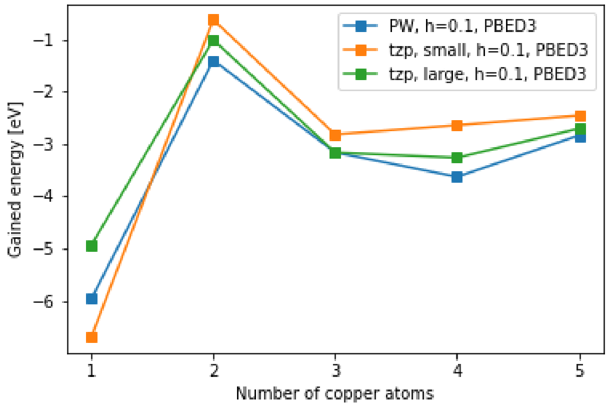

(b) Gained energy.

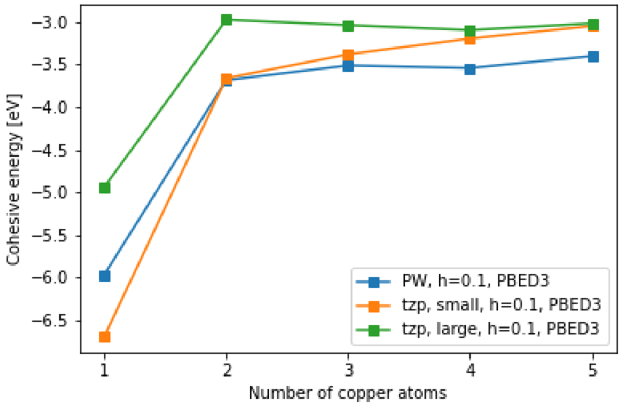

(c) Cohesive energy.

Fig. S29: Stability descriptors of the models calculated on LCAO basis with different size.

The benefit of using the larger basis seems to be clear for interaction and gained energy, while the case of cohesive energy is the opposite. As we mentioned in Sec. 2 of

the Main Text, the electronic structure analysis was performed using LCAO basis, which have several benefits, e.g. the computational time and memory usage can be drastically reduced. However, for the LCAO basis sets, the basis set superposition error (BSSE) [18] can be large. This occurs due to the incompleteness of the bases, where one of the fragment's basis functions improve the description of the other's electronic structure. The error can be computed using the counterpoise correction by Boys and Bernardi [19]:

$$E'_{tot} = E_{tot} - \left(E_B^{ghost} - E_B + E_A^{ghost} - E_A\right), \quad (1)$$

where E'_{tot} is the corrected total energy, E_A and E_B are the total energy of the fragments, while E_B^{ghost} or E_A^{ghost} also belong to the separated systems, where the complementary parts (i.e. N_4V_2 in case of cluster and vice versa) are replaced with 'ghost' atoms, meaning that only basis functions are presented without nuclei or electrons. The compensated energy terms ('bssecomp' abbreviation in Tables S4-S6) with the same settings, used before, are demonstrated on Figure S30.

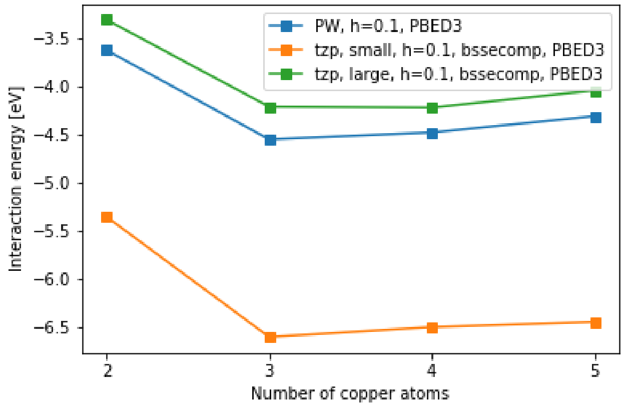

(a) Interaction energy.

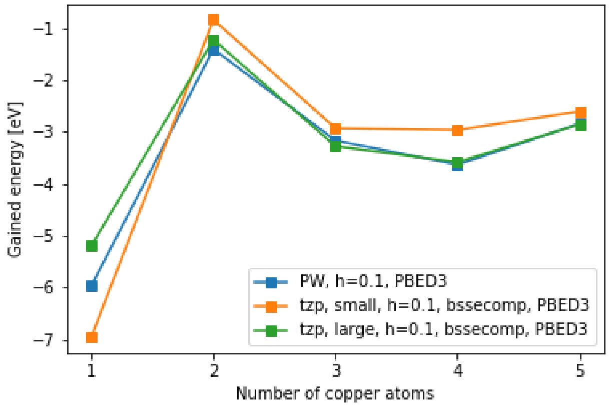

(b) Gained energy.

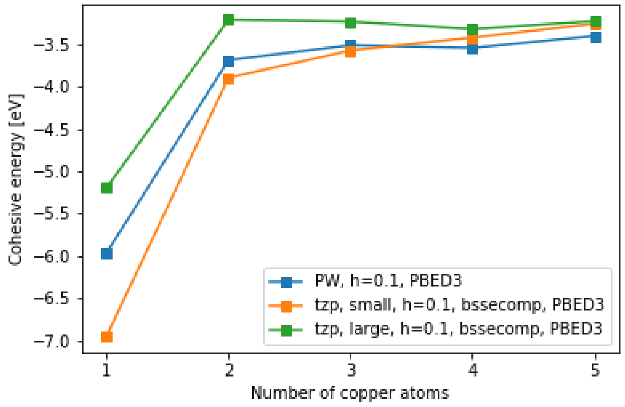

(c) Cohesive energy.

Fig. S30: Stability descriptors of the models calculated on LCAO basis with BSSE correction.

The BSSE correction improved considerably the deviance from the reference values

calculated using PW basis. Based on the results, presented and discussed so far, the stability of the structures were analysed using the parameters from reference computations (for the sake of accurate, quantitative results), while the geometry optimizations and the electronic structure computations were performed using the smaller TZP basis, but with high density k-point mesh of $(10, 10, 1) + \Gamma$, since the electronic states can be influenced strongly by the precision of sampling scheme.

4.2 Band structure analysis of systems studied under vacuum conditions

According to the best of our knowledge, the electronic structure of N_4V_2-Cu_n $(n = 2 - 5)$ has not been explored so far (neither in vacuum nor under electrochemical conditions), therefore we intended to perform a systematic mapping of the electronic states in momentum space. The computations were spin-polarized but for the sake of transparency, we did not distinguish the states with different polarization in notation. We used pristine graphene as a reference, where the band structure along the high-symmetry $\Gamma - M - K - \Gamma$ path in the first Brillouin-zone, additionally the Density of States (DOS) and the orbital projected DOS (PDOS) are illustrated on Figure S31.

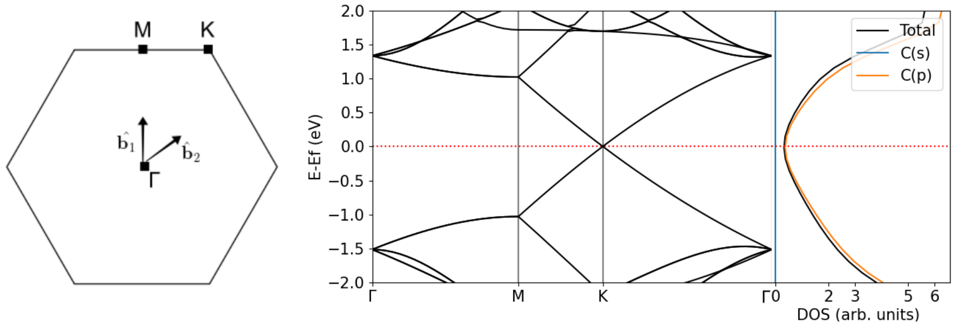

Fig. S31: The first Brillouin-zone, band structure, total and orbital projected DOS of pristine graphene.

It is well know, that graphene has two non-equivalent Dirac-points, where the valence and the conduction bands are knotted and in ideal case (without defects, deformations and dopants) and in ground state (no excitations), the Fermi-level is located at these points [20]. As expected, one of the Dirac-points is located at point K and the linear part of the dispersion relation, the *Dirac-cone* is formed by the p-type orbitals of carbon atoms (these also form the π-bonds). The presence of the pyridinic defect reduces the symmetry of the surface. Considering the first Brillouin-zone, it means, that the number

of equivalent points reduces. As Figure S32 shows, we named the vertices and midpoints of the hexagon in k-space and gave their coordinates in terms of the reciprocal vectors in Table S7.

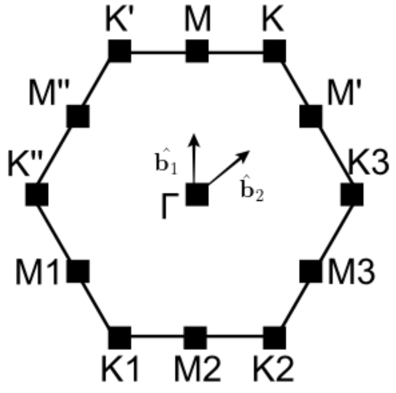

Fig. S32: Hexagonal Brillouin-zone with the potential, high-symmetry points.

Tab. S7: The coordinates of the selected points in the two-dimensional, hexagonal Brillouin-zone, in terms of the reciprocal vectors.

**Name of the k-point**	**Coordinates of the k-point** $(\hat{b}_1, \hat{b}_2, \hat{b}_3)$
Γ	(0,0,0)
**M**	(1/2,0,0)
**K**	(1/3,1/3,0)
**M'**	(0,1/2,0)
**K3**	(-1/3,2/3,0)
**M3**	(-1/2,1/2,0)
**K2**	(-2/3,1/3,0)
**M2**	(-1/2,0,0)
**K1**	(-1/3,-1/3,0)
**M1**	(0,-1/2,0)
**K"**	(1/3,-2/3,0)
**M"**	(1/2,-1/2,0)
**K'**	(2/3,-1/3,0)

The definition of the reciprocal vectors is the following [20]:

$$\hat{b}_1 = \frac{4\pi}{3}\frac{1}{a}\left(0,1,0\right), \hat{b}_2 = \frac{4\pi}{3}\frac{1}{a}\left(\sqrt{3},1,0\right), \hat{b}_3 = \left(0,0,0\right), \tag{2}$$

where $a = 1.42$ Å is the lattice constant. The identification of the non-equivalent points was accomplished by a procedure, where the band structure was calculated and depicted along a triangle (sampling it in 80 equidistant points), appointed by three of the previously discussed points (one of them was always the origin Γ, the other two were a midpoint and a vertex). Our aim was to identify the equivalent points based on the real-space symmetries of the geometry. The band structure of N_4V_2 has been already studied [4, 21], though the precise definition of high-symmetry points may differ, i.e. $\pi/3$ angle is closed by the unit cell vectors and $2\pi/3$ between k-vectors or vice versa (we used the latter convention). Along the classified, equivalent paths, the band structure of N_4V_2, in the vicinity of the Fermi-level (low excitation part is relevant here) is illustrated on Figures S33-S35.

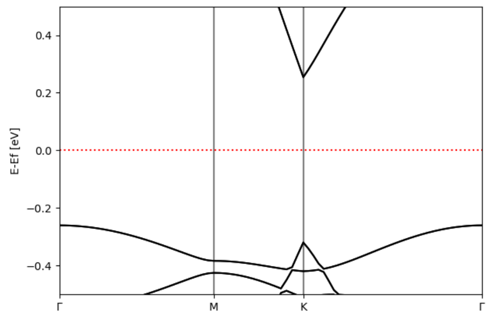

(a) Path: $\Gamma M K \Gamma$.

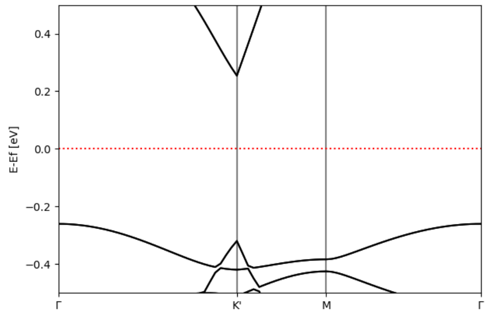

(b) Path: $\Gamma K^{'} M \Gamma$.

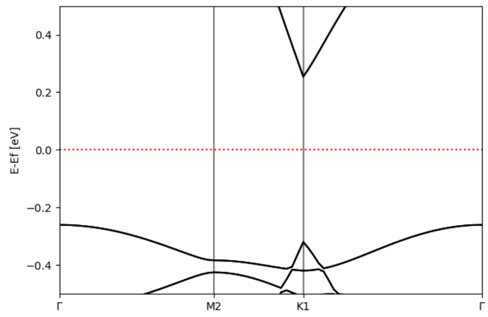

(c) Path: $\Gamma M2K1\Gamma$.

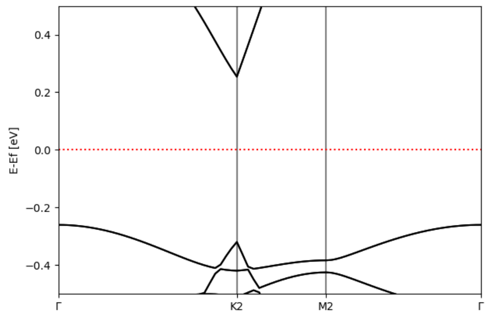

(d) Path: $\Gamma K2M2\Gamma$.

Fig. S33: Band structure of N_4V_2 along equivalent paths.

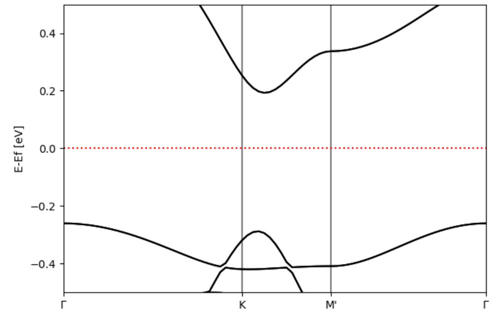

(a) Path: $\Gamma K M^{'} \Gamma$.

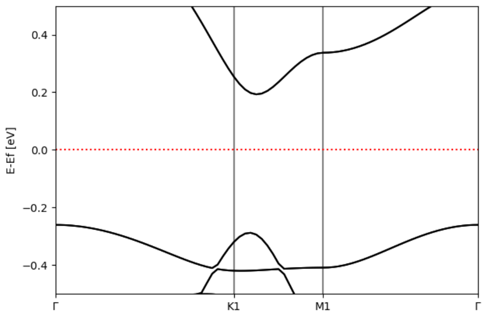

(b) Path: $\Gamma K1 M1 \Gamma$.

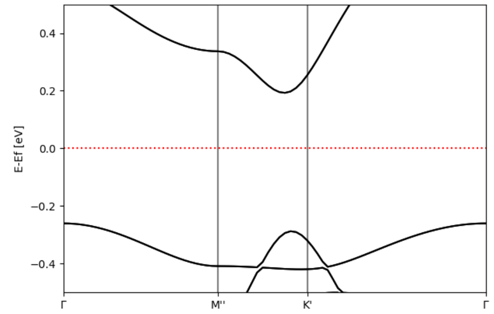

(c) Path: $\Gamma M^{''} K^{'} \Gamma$.

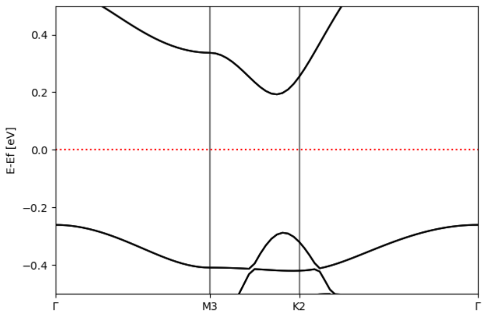

(d) Path: $\Gamma M3 K2 \Gamma$.

Fig. S34: Band structure of N_4V_2 along equivalent paths.

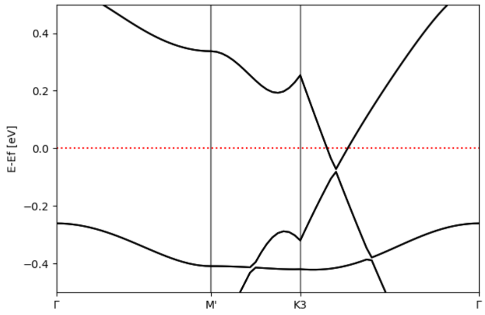

(a) Path: $\Gamma M' K3 \Gamma$.

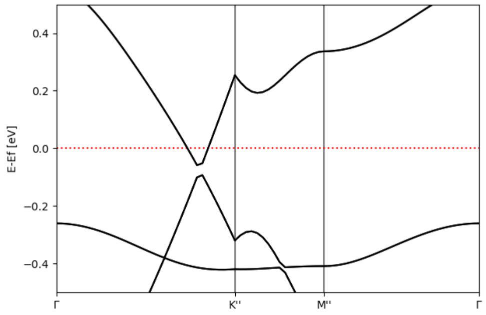

(b) Path: $\Gamma K'' M'' \Gamma$.

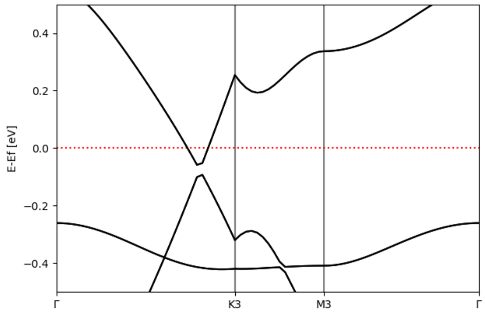

(c) Path: $\Gamma K3 M3 \Gamma$.

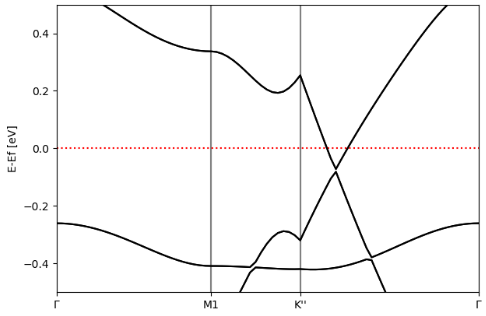

(d) Path: $\Gamma M1 K'' \Gamma$.

Fig. S35: Band structure of N_4V_2 along equivalent paths.

We recognized that structures N_4V_2 and N_4V_2-Cu have common equivalent paths in their first Brillouin-zone. This is demonstrated on Figures S36-S38.

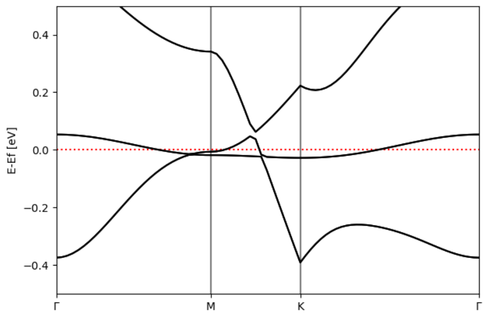

(a) Path: $\Gamma MK\Gamma$.

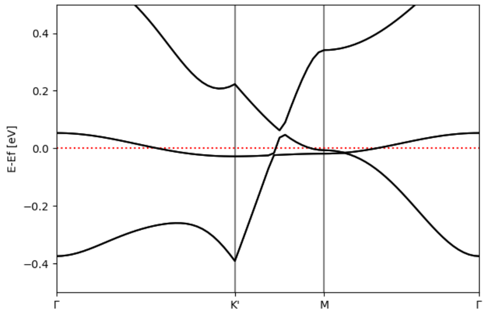

(b) Path: $\Gamma K^{'}M\Gamma$.

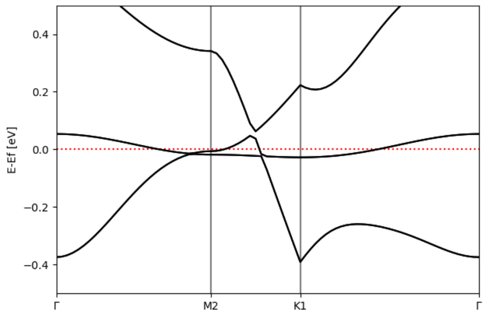

(c) Path: $\Gamma M2K1\Gamma$.

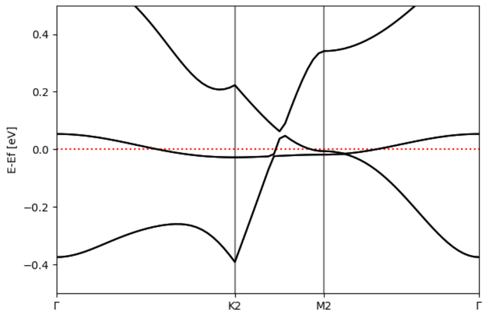

(d) Path: $\Gamma K2M2\Gamma$.

Fig. S36: Band structure of N_4V_2-Cu along equivalent paths.

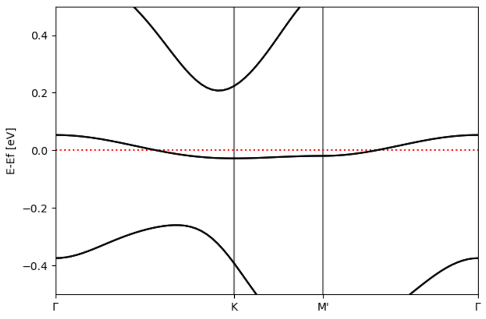

(a) Path: $\Gamma K M^{'} \Gamma$.

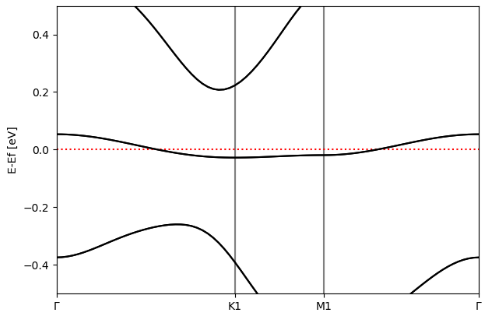

(b) Path: $\Gamma K1M1\Gamma$.

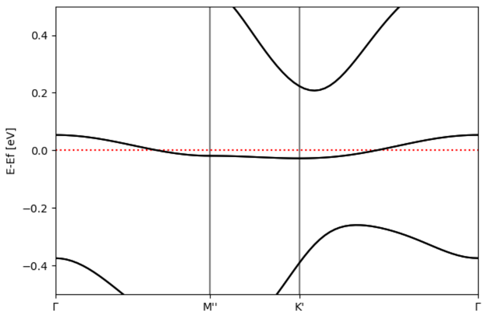

(c) Path: $\Gamma M^{''} K^{'} \Gamma$.

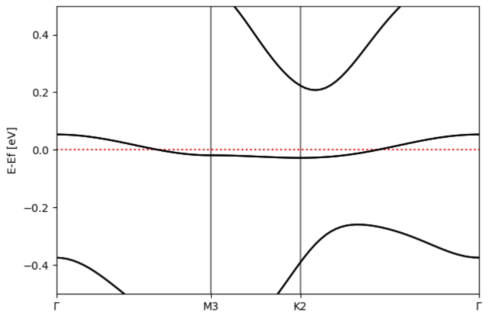

(d) Path: $\Gamma M3K2\Gamma$.

Fig. S37: Band structure of N_4V_2-Cu along equivalent paths.

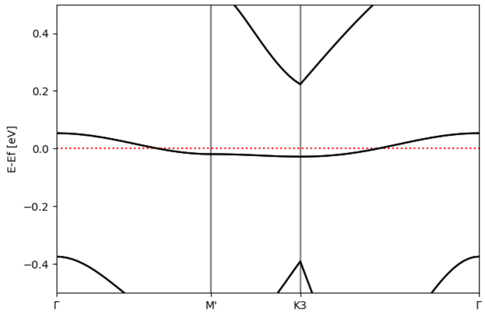

(a) Path: $\Gamma M' K3\Gamma$.

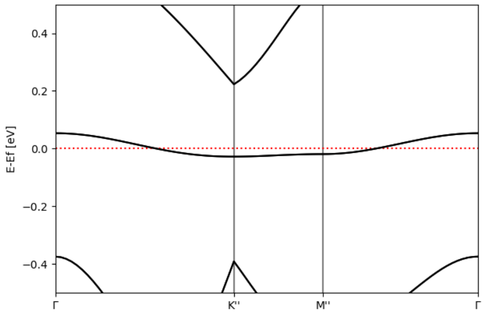

(b) Path: $\Gamma K'' M''\Gamma$.

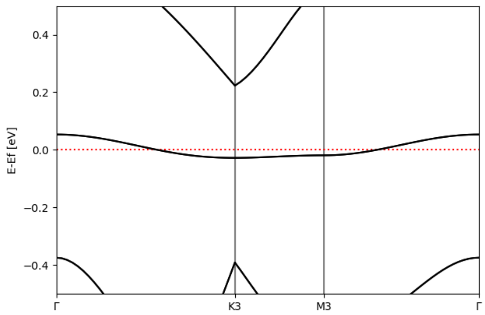

(c) Path: $\Gamma K3M3\Gamma$.

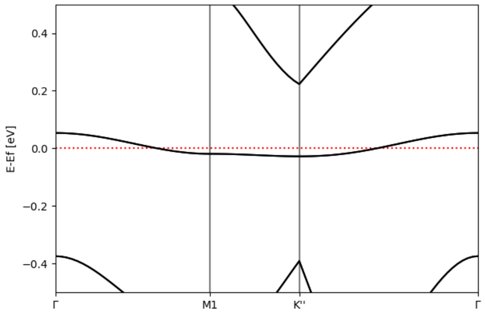

(d) Path: $\Gamma M1K''\Gamma$.

Fig. S38: Band structure of N_4V_2-Cu along equivalent paths.

In both cases, the equivalent points are easy to identify, especially, if a closer look is taken at the real space structures, given by Figures S2 and S39.

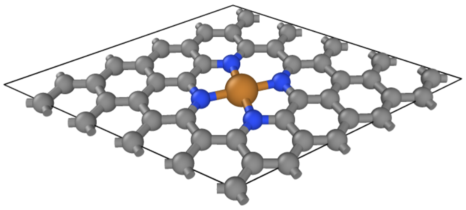

Fig. S39: Optimized geometry of N_4V_2-Cu. The simulation cell in the periodic directions is also shown.

From the original D_{6h} point group of graphene, D_{2h} is obtained, where the two main mirror axes are illustrated on Figure S40, by dashed lines.

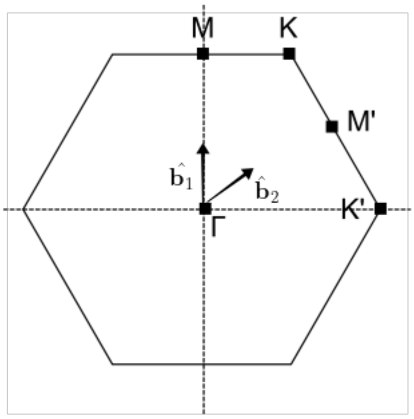

Fig. S40: Brillouin-zone of N_4V_2 and N_4V_2-Cu with non-equivalent, high-symmetry k-points.

As Figure S40 shows, we found four non-equivalent, high-symmetry points in addition to Γ: M, K, M', and K' ($K3$ on Figure S32 and in Table S7). For the sake of comparability with the results of reference [4], the $K'\Gamma M'K\Gamma MK$ path was selected for the analysis of the band structures. The band structure, DOS and orbital projected DOS, furthermore one of the Dirac-points of N_4V_2 is presented in Figure S41.

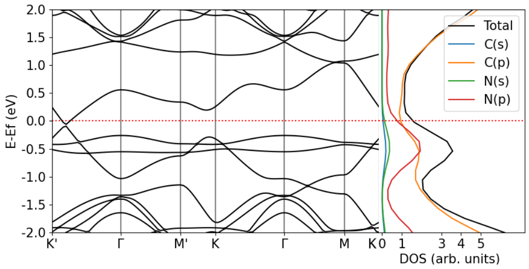

(a) Band structure, total and orbital projected DOS of N_4V_2.

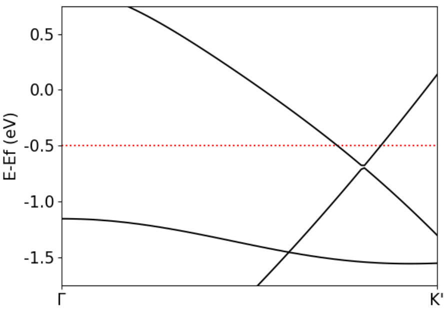

(b) The Dirac-point of N_4V_2.

Fig. S41: The band structure, total, orbital projected DOS and the highlighted Dirac-point of N_4V_2.

Although, on Figure S41a, the Fermi-level does not cross the Dirac-point precisely, it is due to the finite number of k-points and the relatively large smearing (0.1 eV) used in the mapping of the path. Computations using a denser grid (having 120 instead of 80 equidistant points) showed that the Dirac-point remains closed (Figure S41b). Two flat bands can be observed close to the Fermi-level. The PDOS shows that these electronic states are formed by defect atoms, i.e., relatively narrow bands appeared after the hybridization of p-type orbitals of carbon and nitrogen atoms. In the next step, we followed

the same procedure as before, when the band structure of N_4V_2-Cu_n ($n = 2 - 5$) was studied. Although, we did not perform the same electronic structure analysis as we did in case of graphene (Figure S31) and N_4V_2 (Figure S41), since these mean novelty for N_4V_2-Cu_n ($n = 1 - 5$) and are discussed in Sec. 3.2 of the Main Text and also in Section 5.1. The mappings of band structures are depicted on Figures S42-S45, for N_4V_2-Cu_2, N_4V_2-Cu_3, N_4V_2-Cu_4 and N_4V_2-Cu_5, respectively.

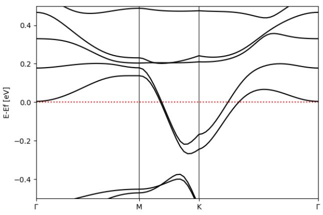

(a) Path: $\Gamma MK\Gamma$.

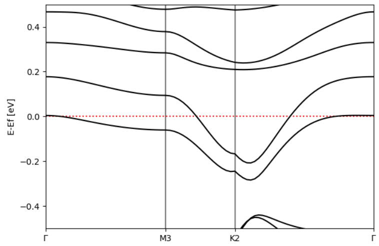

(b) Path: $\Gamma M3K2\Gamma$.

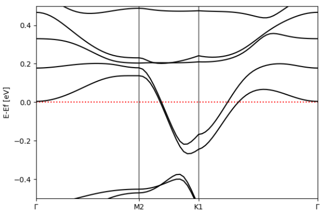

(c) Path: $\Gamma M2K1\Gamma$.

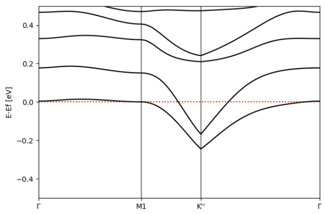

(d) Path: $\Gamma M1K''\Gamma$.

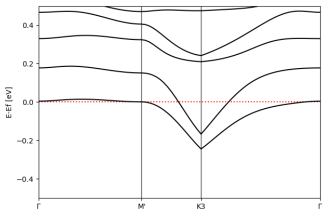

(e) Path: $\Gamma M'K3\Gamma$.

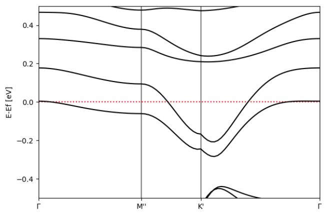

(f) Path: $\Gamma M''K'\Gamma$.

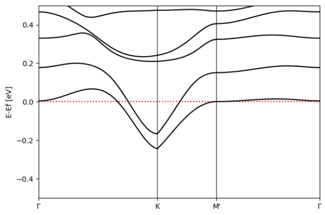

(g) Path: $\Gamma KM'\Gamma$.

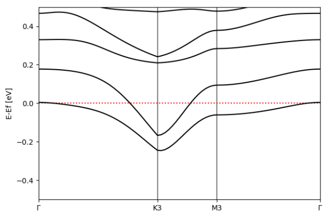

(h) Path: $\Gamma K3M3\Gamma$.

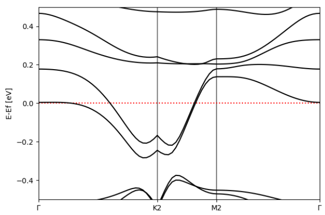

(i) Path: $\Gamma K2M2\Gamma$.

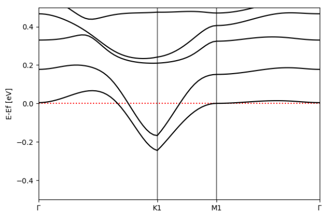

(j) Path: $\Gamma K1M1\Gamma$.

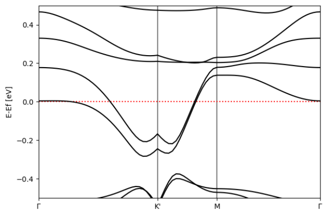

(k) Path: $\Gamma K'M\Gamma$.

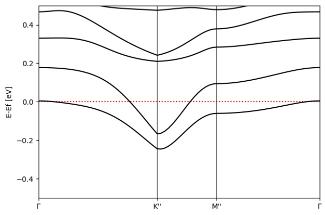

(l) Path: $\Gamma K''M''\Gamma$.

Fig. S42: Band structure of N_4V_2-Cu_2 along different paths.

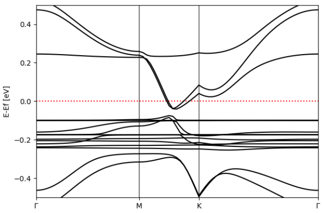

(a) Path: $\Gamma MK\Gamma$.

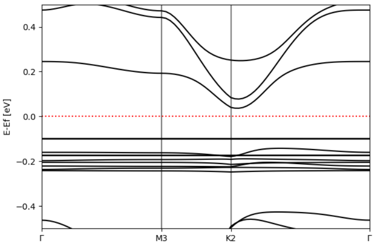

(b) Path: $\Gamma M3K2\Gamma$.

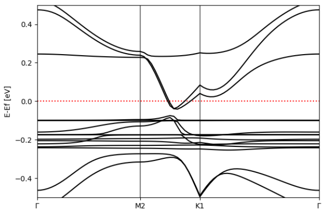

(c) Path: $\Gamma M2K1\Gamma$.

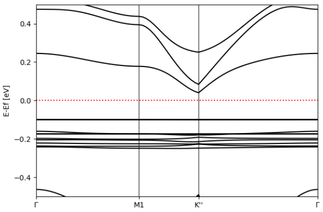

(d) Path: $\Gamma M1K''\Gamma$.

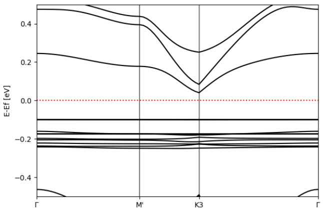

(e) Path: $\Gamma M'K3\Gamma$.

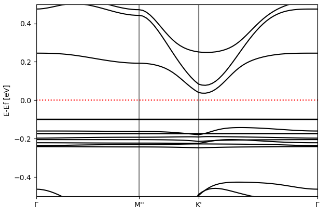

(f) Path: $\Gamma M''K'\Gamma$.

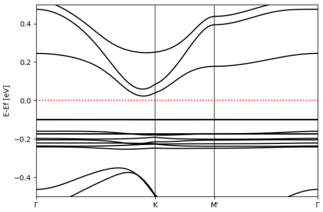

(g) Path: $\Gamma KM'\Gamma$.

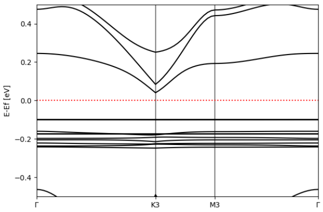

(h) Path: $\Gamma K3M3\Gamma$.

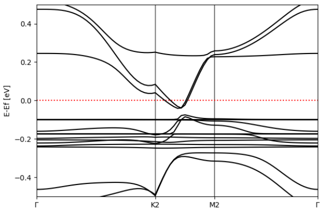

(i) Path: $\Gamma K2M2\Gamma$.

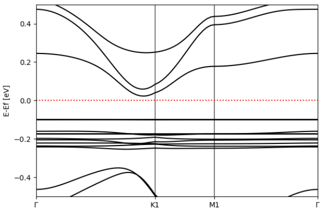

(j) Path: $\Gamma K1M1\Gamma$.

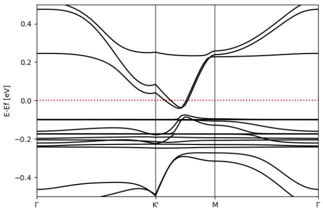

(k) Path: $\Gamma K'M\Gamma$.

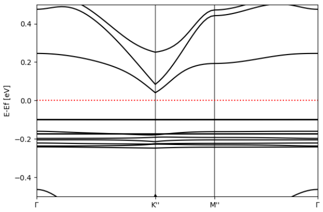

(l) Path: $\Gamma K''M''\Gamma$.

Fig. S43: Band structure of N_4V_2-Cu_3 along different paths.

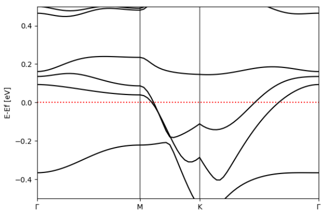

(a) Path: $\Gamma M K \Gamma$.

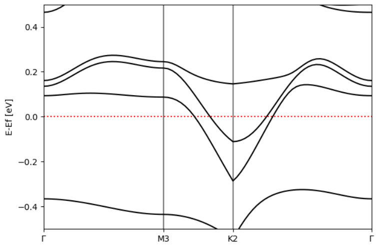

(b) Path: $\Gamma M3K2\Gamma$.

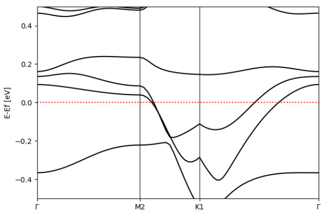

(c) Path: $\Gamma M2K1\Gamma$.

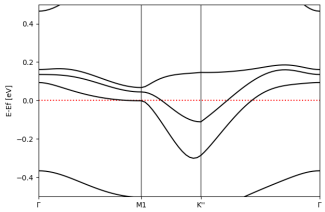

(d) Path: $\Gamma M1K''\Gamma$.

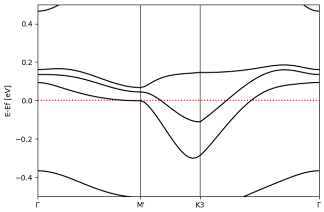

(e) Path: $\Gamma M'K3\Gamma$.

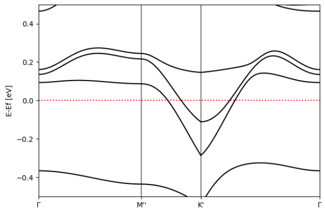

(f) Path: $\Gamma M''K'\Gamma$.

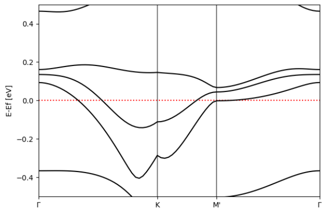

(g) Path: $\Gamma K M'\Gamma$.

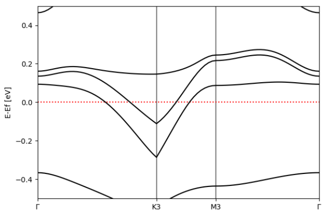

(h) Path: $\Gamma K3M3\Gamma$.

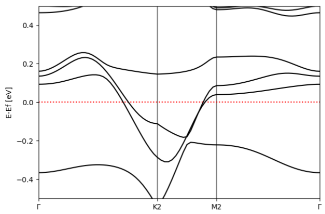

(i) Path: $\Gamma K2M2\Gamma$.

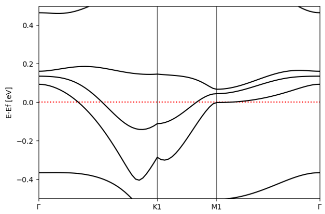

(j) Path: $\Gamma K1M1\Gamma$.

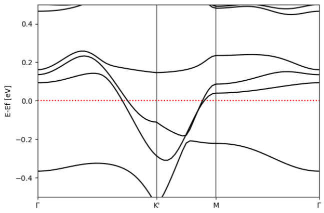

(k) Path: $\Gamma K'M\Gamma$.

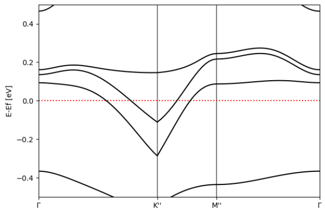

(l) Path: $\Gamma K''M''\Gamma$.

Fig. S44: Band structure of N_4V_2-Cu_4 along different paths.

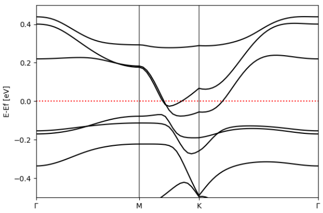

(a) Path: $\Gamma M K \Gamma$.

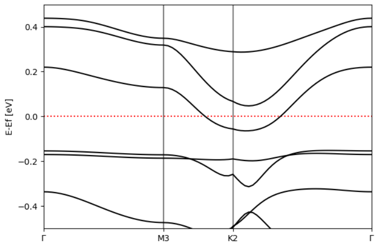

(b) Path: $\Gamma M3K2\Gamma$.

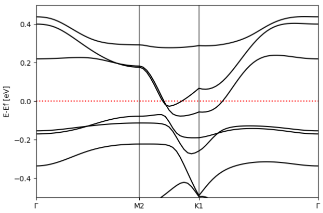

(c) Path: $\Gamma M2K1\Gamma$.

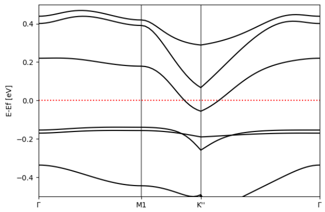

(d) Path: $\Gamma M1K^{''}\Gamma$.

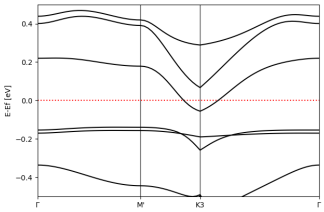

(e) Path: $\Gamma M^{'}K3\Gamma$.

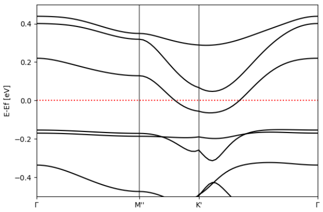

(f) Path: $\Gamma M^{''}K^{'}\Gamma$.

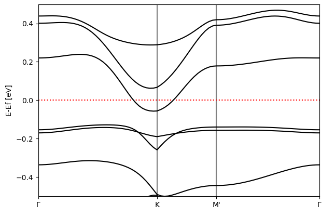

(g) Path: $\Gamma K M^{'}\Gamma$.

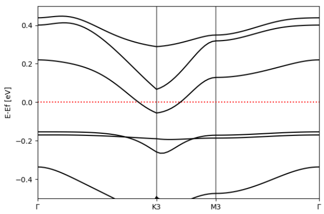

(h) Path: $\Gamma K3M3\Gamma$.

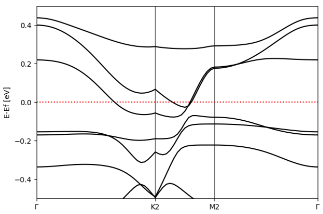

(i) Path: $\Gamma K2M2\Gamma$.

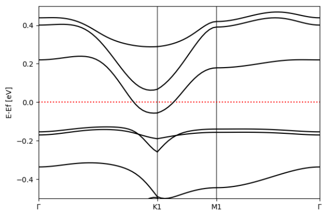

(j) Path: $\Gamma K1M1\Gamma$.

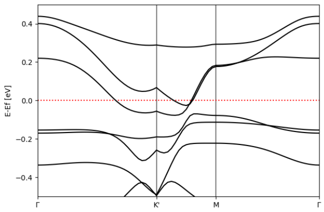

(k) Path: $\Gamma K^{'}M\Gamma$.

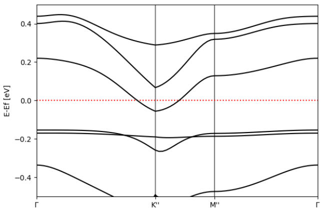

(l) Path: $\Gamma K^{''}M^{''}\Gamma$.

Fig. S45: Band structure of N_4V_2-Cu_5 along different paths.

Although, the analysis of the band structure along the different paths for each of N_4V_2-Cu_n ($n = 2 - 5$) suggests, that the geometries should be invariant under inversion (the origin is the midpoint of the vacancy), the presence of the clusters by itself breaks the inversion symmetry. Moreover, the results of the geometry optimization, discussed in Section 3.1 of the Main Text, demonstrate that the interaction between clusters and the N_4V_2 bends the substrate itself. Strictly speaking, this deformation also leads to the loss of the inversion symmetry. However, it is important to note that GPAW (like most atomic simulation codes) recognizes symmetry within a pre-defined tolerance level (10^{-7}

Å) [22]. While the distortion of N_4V_2 (and also because of the lower symmetry of the bound copper cluster in several cases) is certainly above this threshold, the symmetry breaking has only a small effect on the energy levels. Without inversion symmetry, the N_4V_2-Cu_n ($n = 2 - 5$) have C_1 point group symmetry and Brillouin-zone, presented on Figure S46.

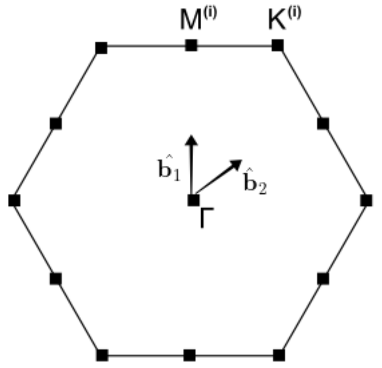

Fig. S46: Brillouin-zone of N_4V_2-Cu_n ($n = 2 - 5$).

In order to include all of the non-equivalent paths, the final version was $\Gamma M'' K'' \Gamma M K \Gamma M' K' \Gamma$ for each structure containing cluster, where the notation of k-points is consistent with Figure S32 and Table S7.

4.3 Applicability of GCP-K theory

Firstly, based on references [23, 24, 25], we briefly review the theory of *Grand Canonical Potential Kinetics* (GCP-K). Starting from basic thermodynamic considerations, the fixed-charge *free energy* $F(N)$ can be converted into the *grand canonical potential* $G(N, U)$ using Legendre-transformation:

$$G(N, U) = F(N) + Ne\,(U_{SHE} - U)\,, \tag{3}$$

where e is equal to the charge of the electrons. The potential of the *standard hydrogen electrode* (SHE) U_{SHE} is a reference parameter (see the Section 2 of the Main Text). The sign conventions applied here are consistent with the electrochemical experiments, i.e., we use the total charge of the systems, not the number of the excess electrons. In equilibrium, $G(N, U)$ is minimal as the function of the number of the charge carriers. This leads to an optimization problem, what can be solved by varying N:

$$\frac{dG(N, U)}{dN} = 0 \Leftrightarrow \mu = \frac{dF(N)}{dN} = e\,(U_{SHE} - U)\,. \tag{4}$$

The relation between $F(N)$ and N is assumed to be quadratic:

$$F(N) = a\,(N - N_0)^2 + b\,(N - N_0) + c, \tag{5}$$

where N_0 denotes the number of the charge carriers, when the potential is equal to the so-called *potential of zero charge* (PZC) $U = U_{PZC}$. $F(N)$ can be extracted from the $jDFTx$ calculations, while the parameters a, b and c are determined by quadratic function fitting. Using Equations (4) and (5), the analytic form of $G(U)$ in equilibrium is given by the equation below:

$$G(U) = -\frac{1}{4a}\,(b - \mu_{SHE} - eU)^2 + c - N_0\mu_{SHE} + N_0 eU. \tag{6}$$

Here, $\mu_{SHE} = eU_{SHE}$ is the chemical potential of the SHE. The parameters a, b and c obtain physical meaning using Equations (3), (4) and (5):

$$\frac{1}{2a} = C_{diff} = -\frac{\partial N}{\partial U} \tag{7}$$

is the so-called *differential capacitance*, which can be originated from broad, partially occupied electronic bands, where the number of the electrons can vary continuously, leading to capacitive behaviour.

$$b = \mu_{SHE} - eU_{PZC} \tag{8}$$

$$c = F(U_{PZC)}), \tag{9}$$

which gives simply the free energy of the system, where the potential is equal to U_{PZC}. We have tested the validity of the theory on the models containing copper atoms by fitting a second order polynomial function (see Equation (5)) on the computed free energy values $F(N)$ with respect to the deviance from neutral electron population, $N - N_0$. Firstly, $N_0 - N$ was computed and plotted on Figure S47 as the function of the electrode potential $U - U_{SHE}$. The red, horizontal line shows the PZC of the models, which goes from 0.01 V (for N_4V_2) to -0.97 V (for N_4V_2-Cu_4).

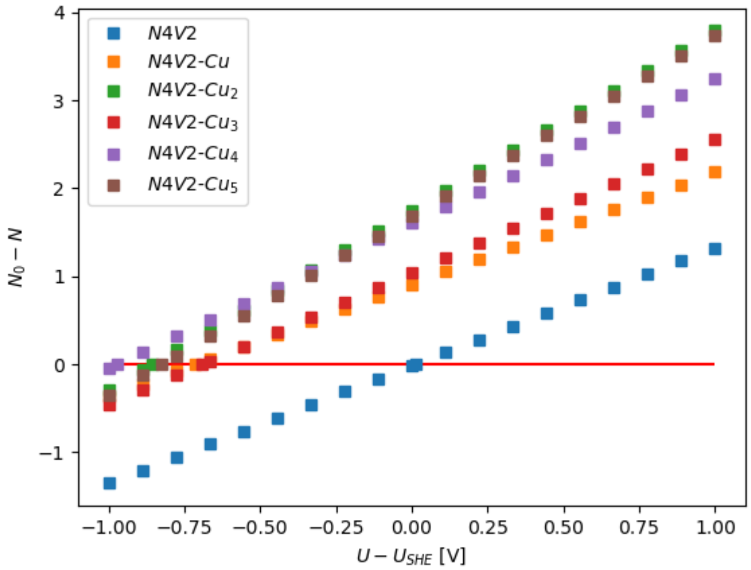

Fig. S47: $N_0 - N$ of the models with respect to $U - U_{SHE}$.

Meanwhile, the chemical potential $\mu(N)$ was determined in two ways: firstly, we performed self-consistent $jDFTx$ simulations. On the other hand, we substituted the parameters of the mentioned fitting into Equation (3). For the sake of reproducibility, we tabulated the computed free energies and chemical potentials into Tables S8-S9, respectively.

Tab. S8: The free energy of N_4V_2-Cu_n ($n = 1 - 5$) with respect to $N - N_0$.

$N - N_0$	**Free energy of** $N4V2$-Cu **(eV)**	**Free energy of** $N4V2$-Cu_2 **(eV)**	**Free energy of** $N4V2$-Cu_3 **(eV)**	**Free energy of** $N4V2$-Cu_4 **(eV)**	**Free energy of** $N4V2$-Cu_5 **(eV)**
-2.00	-13407.581	-18898.203	-24389.028	-29881.133	-35372.238
-1.50	-13410.328	-18900.700	-24391.642	-29883.609	-35374.633
-1.00	-13412.837	-18902.969	-24394.072	-29885.881	-35376.882
-0.50	-13415.105	-18905.020	-24396.308	-29887.947	-35378.984
0.00	-13417.130	-18906.890	-24398.346	-29889.814	-35380.936
0.50	-13418.932	-18908.656	-24400.196	-29891.515	-35382.748
1.00	-13420.573	-18910.355	-24401.904	-29893.097	-35384.458
1.50	-13422.092	-18912.021	-24403.499	-29894.600	-35386.092
2.00	-13423.525	-18913.629	-24405.008	-29896.064	-35387.668

Tab. S9: The chemical potential of N_4V_2-Cu_n ($n = 1-5$) with respect to $N - N_0$.

$N - N_0$	**Chemical potential of** $N4V2$-Cu **(eV)**	**Chemical potential of** $N4V2$-Cu_2 **(eV)**	**Chemical potential of** $N4V2$-Cu_3 **(eV)**	**Chemical potential of** $N4V2$-Cu_4 **(eV)**	**Chemical potential of** $N4V2$-Cu_5 **(eV)**
-2.00	-5.729	-5.221	-5.403	-5.152	-4.938
-1.50	-5.257	-4.766	-5.047	-4.749	-4.644
-1.00	-4.777	-4.314	-4.670	-4.338	-4.352
-0.50	-4.292	-3.899	-4.275	-3.928	-4.056
0.00	-3.808	-3.616	-3.874	-3.549	-3.753
0.50	-3.429	-3.458	-3.547	-3.270	-3.511
1.00	-3.145	-3.346	-3.292	-3.072	-3.336
1.50	-2.946	-3.292	-3.098	-2.956	-3.206
2.00	-2.785	-3.136	-2.940	-2.911	-3.096

The results are also presented on Figures S48-S52.

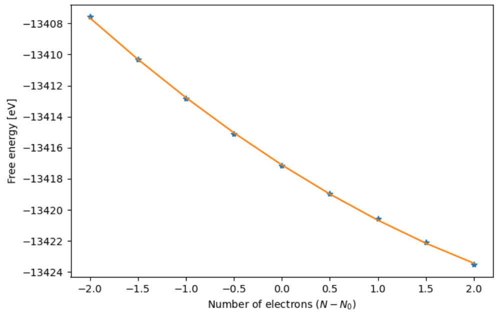

(a) Free energy. Orange curve shows the result of fitting.

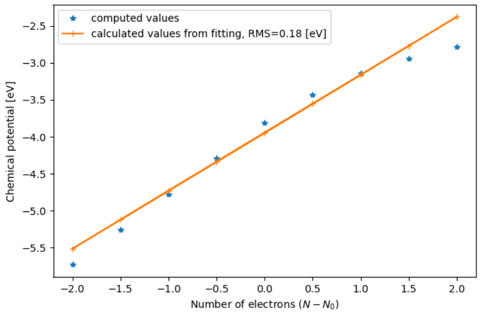

(b) Chemical potential. Orange line shows the theoretical shape of $\mu(N)$ determined by using the parameters of quadratic function fitting.

Fig. S48: Free energy and chemical potential of N_4V_2-Cu with respect to $N - N_0$.

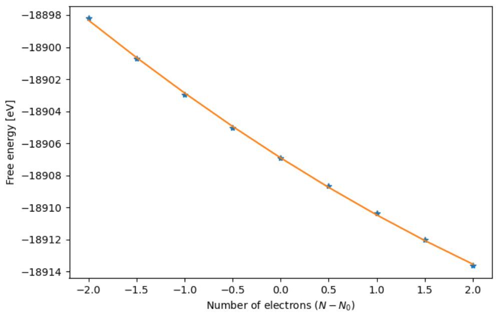

(a) Free energy. Orange curve shows the result of fitting.

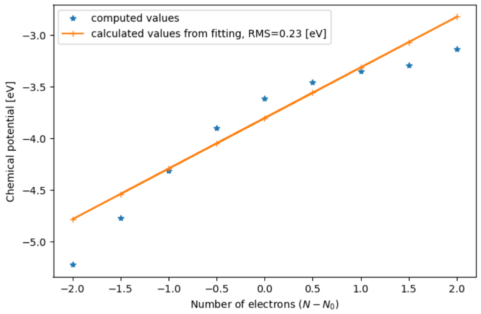

(b) Chemical potential. Orange line shows the theoretical shape of $\mu(N)$ determined by using the parameters of quadratic function fitting.

Fig. S49: Free energy and chemical potential of N_4V_2-Cu_2 with respect to $N-N_0$.

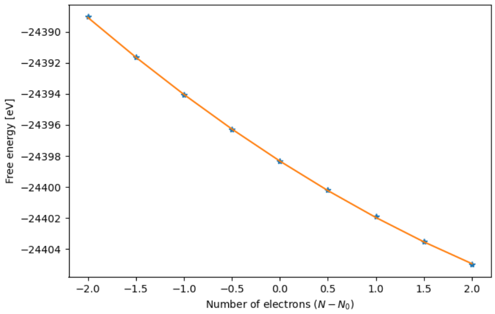

(a) Free energy. Orange curve shows the result of fitting.

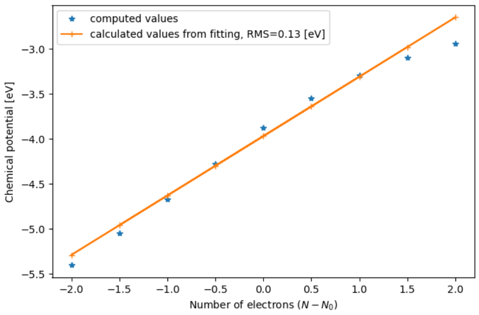

(b) Chemical potential. Orange line shows the theoretical shape of $\mu(N)$ determined by using the parameters of quadratic function fitting.

Fig. S50: Free energy and chemical potential of N_4V_2-Cu_3 with respect to $N-N_0$.

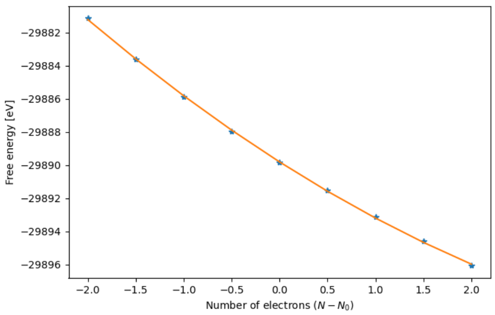

(a) Free energy. Orange curve shows the result of fitting.

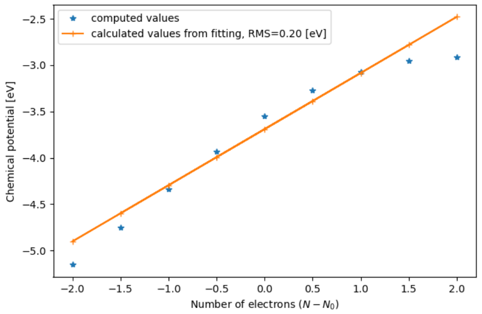

(b) Chemical potential. Orange line shows the theoretical shape of $\mu(N)$ determined by using the parameters of quadratic function fitting.

Fig. S51: Free energy and chemical potential of N_4V_2-Cu_4 with respect to $N - N_0$.

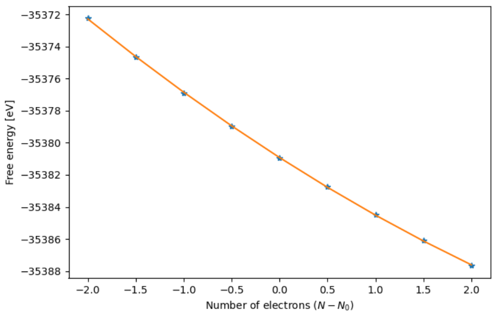

(a) Free energy. Orange curve shows the result of fitting.

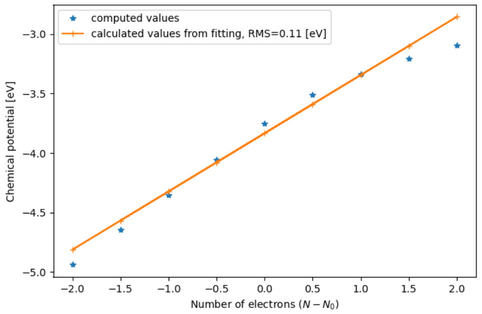

(b) Chemical potential. Orange line shows the theoretical shape of $\mu(N)$ determined by using the parameters of quadratic function fitting.

Fig. S52: Free energy and chemical potential of N_4V_2-Cu_5 with respect to $N - N_0$.

The parameters of the quadratic function fittings are tabulated into Table S10.

Tab. S10: Parameters of quadratic function fitting on $F(N - N_0)$ for structures N_4V_2-Cu_n ($n = 1 - 5$). For definitions, see Equations (7), (8) and (9).

**Structure**	a (eV/e^2)	b (eV)	c (V)
N_4V_2 - Cu	0.392	-3.946	-13417.099
N_4V_2 - Cu_2	0.245	-3.803	-18906.887
N_4V_2 - Cu_3	0.330	-3.968	-24398.309
N_4V_2 - Cu_4	0.303	-3.690	-29889.776
N_4V_2 - Cu_5	0.245	-3.834	-35380.894

The quadratic function fittings demonstrate a good agreement between the values given by calculations and theoretical considerations. The chemical potentials determined in two different ways can be compared by analysing Figures S48b-S52b, where the orange lines show the theoretical shape of $\mu(N)$. In some cases (e.g. N_4V_2-Cu_2) prominent deviation from the ideal values can be observed. As Figures S48b-S52b show, we determined the root mean square values [26] for each line-data pairs. The maximum value, $RMS = 0.23$ eV appeared in case of N_4V_2-Cu_2. These deviations occur probably due to the mixed nature of the electronic states, discussed in Sec. 3.2 of Main Text. GCP-K theory expects the availability of continuously excitable electronic states (see the explanation about C_{diff} below Equation (7)), indicating the linear form of $\mu(N)$. It is non-trivial here, since clusters have originally discrete energy levels [27], i.e., their contribution to the electronic bands may cause the appearance of some plateaus in μ with the change of the electron number occupation. Thereby the applicability of GCP-K theory for the hybrid states, formed by the continuous bands of N_4V_2 and the discrete levels of Cu_n $(n = 1-5)$ is a non-trivial question. The detailed discussion is out of the scope of this paper, we investigated situations, where $N - N_0$ was relatively close to 0 and supposed, that the possible deviations from the theoretical values do not modify significantly the qualitative trends.

4.4 Computation process of the grand canonical potential

We discussed the theory and the main terms of the GCP-K method in Section 4.3, but avoided the detailed analysis of the calculation's steps. Here, we show how the stability descriptors, cohesive, gained and second order energies (for definitions see Equations (2), (3) and (4) in the Main Text) were computed based on $jDFTx$ simulations and post processing.

- 1. Firstly, as presented in Section 4.3, the free energy of the structures was determined with respect to the deviance in the number of electrons from the neutral value N_0, using $jDFTx$ simulations.
- 2. The next step was the Legendre-transformation, discussed in Section 4.3, where the grand canonical framework (effect of electrode potential and charge equalization) was established.
- 3. Finally, we used, that the grand canonical potential, $GCP(N, U)$ has a minimum in N under electrode potential U, where the minimalization gave $GCP(U)$.

These steps are depicted on Figures S53-S57 for models N_4V_2-Cu_n $(n = 1 - 5)$.

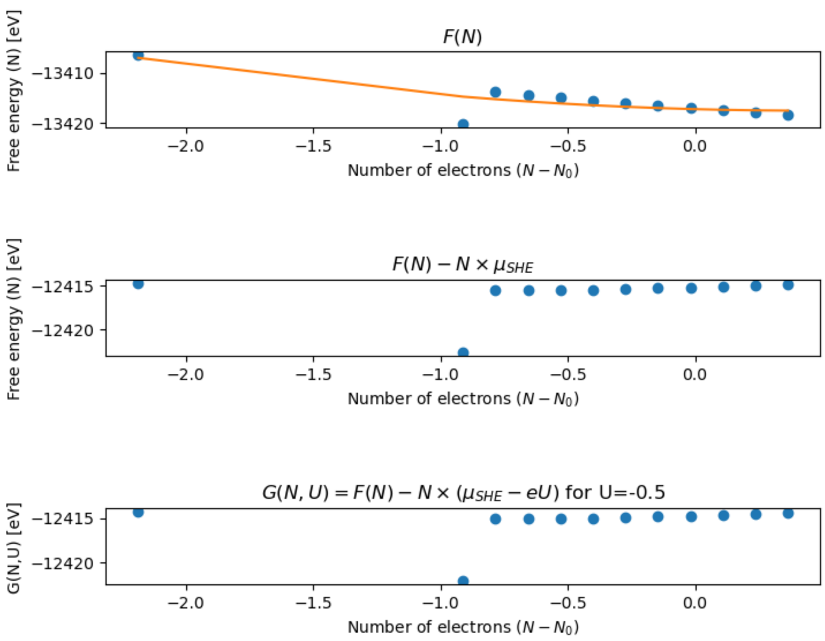

Fig. S53: The main steps of the derivation of $GCP(N, U = -0.5V)$ belonging to N_4V_2-Cu.

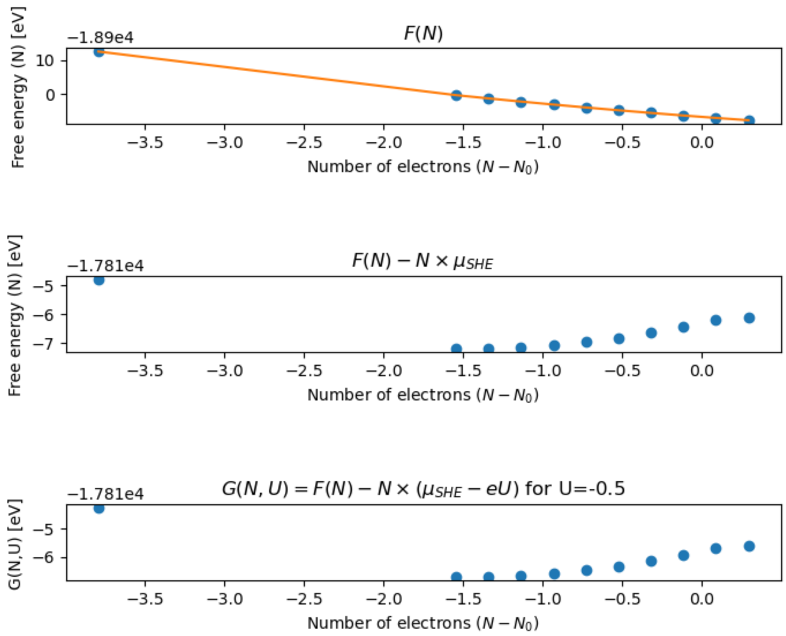

Fig. S54: The main steps of the derivation of $GCP(N, U = -0.5V)$ belonging to N_4V_2-Cu_2.

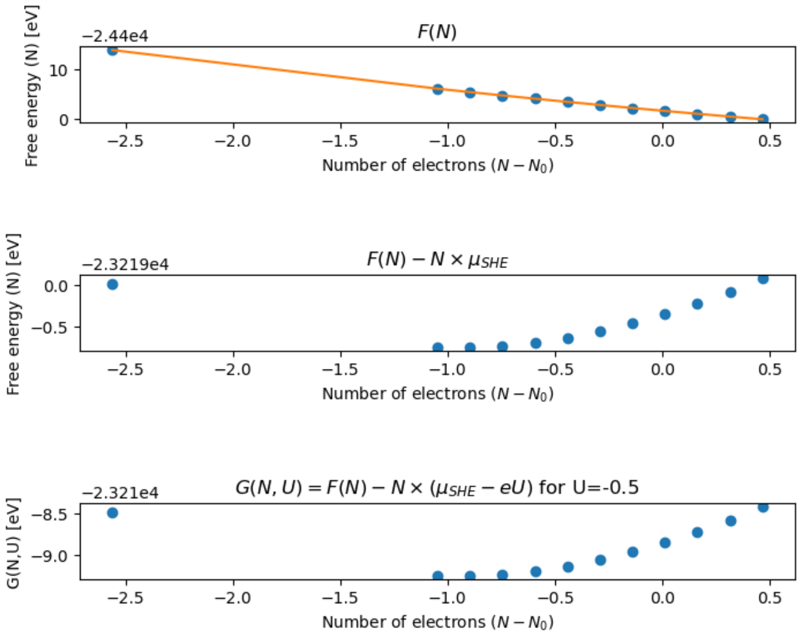

Fig. S55: The main steps of the derivation of $GCP(N, U = -0.5V)$ belonging to N_4V_2-Cu_3.

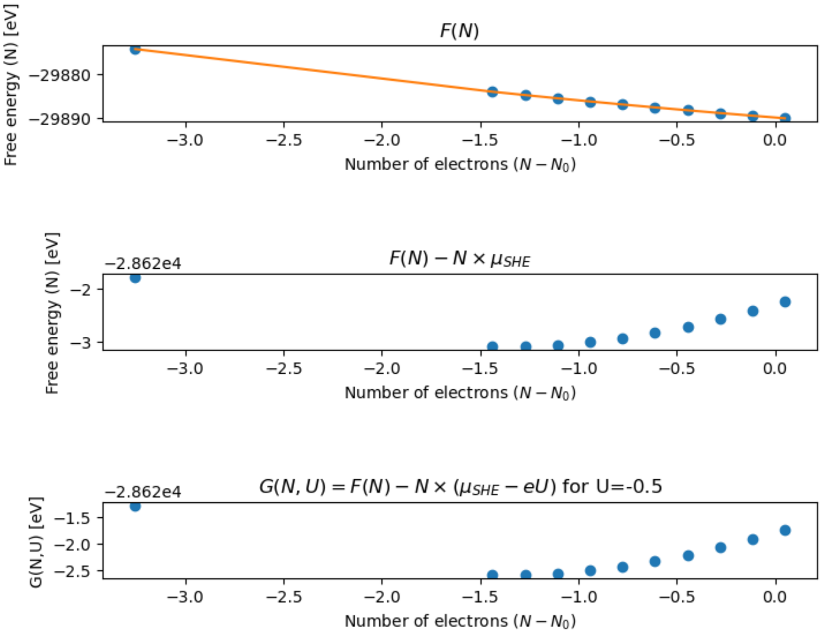

Fig. S56: The main steps of the derivation of $GCP(N, U = -0.5V)$ belonging to N_4V_2-Cu_4.

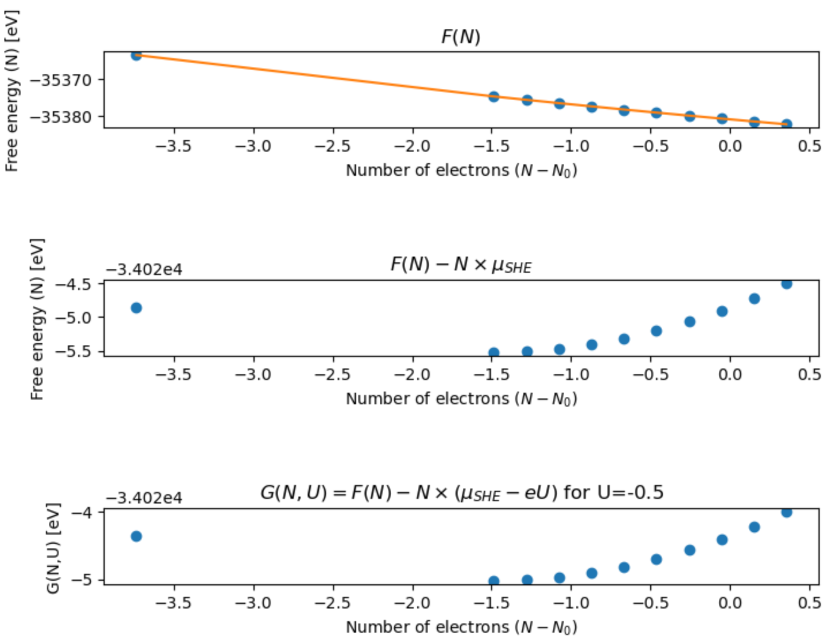

Fig. S57: The main steps of the derivation of $GCP(N, U = -0.5V)$ belonging to N_4V_2-Cu_5.

There is a less complicated way to compute $GCP(U)$, namely, the fitting parameters, listed in Table S10 of Section 4.3 are substituted into the analytical form of $GCP(U)$, presented in Equation (6). We rather followed this procedure and listed the calculated cohesive, gained and second order energies at electrode potentials $U - U_{SHE} = -1.0 - 0.0$ V in Tables S11-S13, respectively.

Tab. S11: The cohesive energy of the models N_4V_2-Cu_n ($n = 1-5$) at electrode potentials $U - U_{SHE} = -1.0 - 0.0$ V

$U - U_{SHE}$ (V)	**Cohesive energy of** $N4V2$-Cu **(eV)**	**Cohesive energy of** $N4V2$-Cu_2 **(eV)**	**Cohesive energy of** $N4V2$-Cu_3 **(eV)**	**Cohesive energy of** $N4V2$-Cu_4 **(eV)**	**Cohesive energy of** $N4V2$-Cu_5 **(eV)**
-1.00	-5.026	-2.060	-2.037	-1.997	-1.912
-0.90	-5.273	-2.174	-2.107	-2.071	-1.967
-0.80	-5.501	-2.281	-2.170	-2.137	-2.017
-0.70	-5.712	-2.379	-2.227	-2.196	-2.061
-0.60	-5.905	-2.469	-2.276	-2.247	-2.101
-0.50	-6.080	-2.551	-2.319	-2.291	-2.135
-0.40	-6.237	-2.625	-2.354	-2.328	-2.164
-0.30	-6.376	-2.692	-2.383	-2.358	-2.188
-0.20	-6.497	-2.750	-2.404	-2.380	-2.207
-0.10	-6.600	-2.800	-2.419	-2.395	-2.220
0.00	-6.685	-2.842	-2.426	-2.403	-2.229

Tab. S12: The gained energy of the models N_4V_2-Cu_n ($n = 1 - 5$) at electrode potentials $U - U_{SHE} = -1.0 - 0.0$ V

$U - U_{SHE}$ (V)	**Gained energy of** $N4V2$-Cu **(eV)**	**Gained energy of** $N4V2$-Cu_2 **(eV)**	**Gained energy of** $N4V2$-Cu_3 **(eV)**	**Gained energy of** $N4V2$-Cu_4 **(eV)**	**Gained energy of** $N4V2$-Cu_5 **(eV)**
-1.00	-5.026	0.906	-1.990	-1.880	-1.570
-0.90	-5.273	0.924	-1.973	-1.962	-1.552
-0.80	-5.501	0.940	-1.950	-2.036	-1.536
-0.70	-5.712	0.955	-1.923	-2.102	-1.524
-0.60	-5.905	0.967	-1.891	-2.160	-1.516
-0.50	-6.080	0.977	-1.854	-2.209	-1.510
-0.40	-6.237	0.986	-1.812	-2.250	-1.508
-0.30	-6.376	0.993	-1.765	-2.283	-1.509
-0.20	-6.497	0.998	-1.714	-2.308	-1.513
-0.10	-6.600	1.001	-1.657	-2.325	-1.520
0.00	-6.685	1.002	-1.596	-2.333	-1.531

Tab. S13: The second order energy of the models N_4V_2-Cu_n ($n = 1-5$) at electrode potentials $U - U_{SHE} = -1.0 - 0.0$ V

$U - U_{SHE}$ **(V)**	**Second order energy of** $N4V2$-Cu **(eV)**	**Second order energy of** $N4V2$-Cu_2 **(eV)**	**Second order energy of** $N4V2$-Cu_3 **(eV)**	**Second order energy of** $N4V2$-Cu_4 **(eV)**	**Second order energy of** $N4V2$-Cu_5 **(eV)**
-1.00	5.932	-2.897	0.111	0.309	-0.406
-0.90	6.197	-2.897	0.011	0.410	-0.498
-0.80	6.442	-2.891	-0.086	0.500	-0.575
-0.70	6.667	-2.877	-0.179	0.578	-0.636
-0.60	6.872	-2.858	-0.269	0.644	-0.683
-0.50	7.057	-2.831	-0.355	0.699	-0.715
-0.40	7.223	-2.798	-0.439	0.743	-0.731
-0.30	7.369	-2.758	-0.518	0.775	-0.733
-0.20	7.495	-2.711	-0.595	0.795	-0.719
-0.10	7.601	-2.658	-0.668	0.805	-0.691
0.00	7.687	-2.598	-0.737	0.802	-0.647

The python script, used to perform the post process calculations is available at reference [11].

5 Notes on spin-polarization

5.1 Systems in vacuum

We also studied the effect of spin-polarization. Graphene is proven to have singlet ground state [20], and the magnetic properties of the different pyridine-type defects have been also examined theoretically [28]. Katten et al. [6] predicted the N_4V_2 model to have ground state with zero magnetic moment under vacuum conditions, confirmed (see below) by our computations as well. Furthermore, we were interested in, whether the models N_4V_2-Cu_n ($n = 1-5$) have finite magnetization in their ground state. The discrete electronic levels of clusters, appearing due to quantum confinement, can be described by shell models [27], where similarly to molecules, and copper clusters show low-spin ground state. However, here the clusters interact with the substrate and the copper atoms form bonds with nitrogens. We performed computations starting with μ_B initial magnetization per atom

for both N_4V_2 and N_4V_2-Cu_n ($n = 1 - 5$) in vacuum, in the latter case, only non-zero initial magnetization was set for the copper atoms. The total energies with the final, total magnetizations are tabulated in Table S14.

Tab. S14: Total energies of the models with μ_B initial magnetization per atom and their final, total magnetization. Here the initial magnetization per atom is regarded to only nitrogen atoms for N_4V_2 and only copper atoms for N_4V_2-Cu_n ($n = 1 - 5$).

**Structure**	**Total energy with zero initial magnetic moment per atom (eV)**	**Total energy with μ_B initial magnetic moment per atom (eV)**	**Final total magnetization (μ_B)**
N_4V_2	-430.370952	-430.370891	0.000
N_4V_2 - Cu	-435.151550	-435.347908	1.001
N_4V_2 - Cu_2	-435.402864	-435.530191	1.222
N_4V_2 - Cu_3	-437.487078	-437.617696	1.034
N_4V_2 - Cu_4	-439.772126	-439.812503	0.661
N_4V_2 - Cu_5	-441.363018	-441.431659	0.801

As expected, N_4V_2 has zero total magnetization in its ground state (the spin-polarized computation also lead to a non-spin-polarized final state). What is more surprising, that independently from the number of copper atoms (even or odd number of electrons) the states with finite magnetic moment are energetically more favourable for N_4V_2-Cu_n ($n = 1 - 5$), than the unpolarized ones. Here, we discuss briefly, how unconstrained magnetic moments are determined in DFT calculations [29]. In self-consistent field (SCF) cycles, the electron density is varied and then the magnitude of the total magnetic moment M is defined and calculated as:

$$M = \mu_B \int (\rho_{up}(\mathbf{r}) - \rho_{down}(\mathbf{r}))\, dr^3, \tag{10}$$

where μ_B is the Bohr-constant, $\rho_{up}(\mathbf{r})$ and $\rho_{down}(\mathbf{r})$ are the polarized electron densities. As the electron density is modified, the magnetic moment varies as well, and depending on where the computation converges (strongly parameter-dependent), it can end up at different values. GPAW also provides local magnetic moments [22] by partitioning the polarized, total electron density on the real space grid. These values are rather qualitative, since they depend strongly on how the real space separation of atoms is defined, which is a non-trivial question. Nevertheless, we present these local moments on Figure S58 for nitrogen and copper atoms (carbon atoms have 2 or even 3 orders of magnitude smaller

moments).

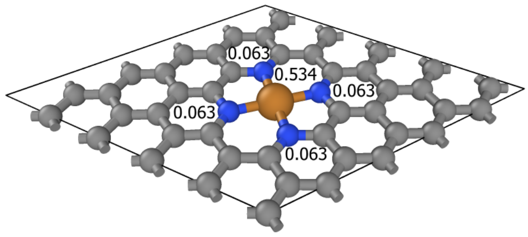

(a) N_4V_2-Cu.

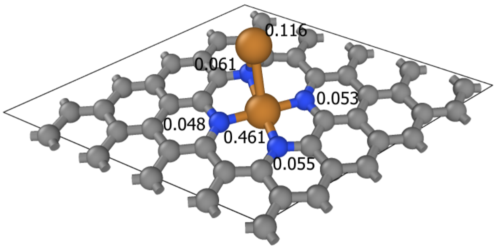

(b) N_4V_2-Cu_2.

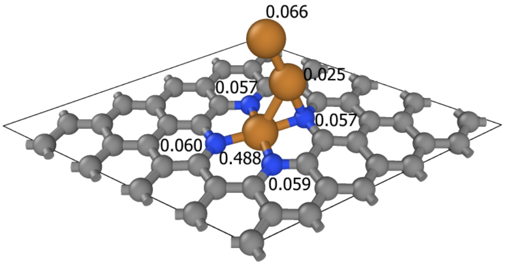

(c) N_4V_2-Cu_3.

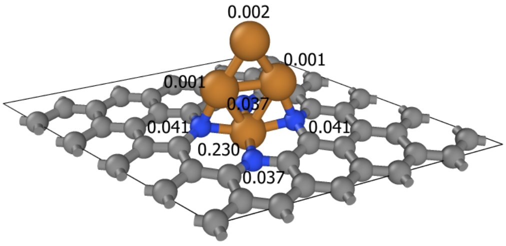

(d) N_4V_2-Cu_4.

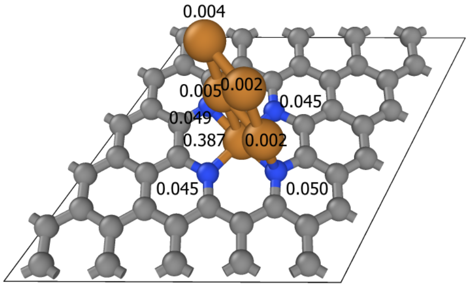

(e) N_4V_2-Cu_5.

Fig. S58: Local magnetic moments (in unit of μ_B) of nitrogen and copper atoms. The simulation cell in the periodic directions is also shown.

The distribution of the local moments symmetric on the nitrogen defect, and in each case, the copper atom, located at the centre of the vacancy has the largest magnetization. These figures demonstrate, that due to the hybridization of the states of nitrogen and cop-

per atoms, it is difficult to assign a definite magnetic moment to them, the polarized electron density is rather diffused. This type of spin-polarization can be *spin-contamination* [30].

For the sake of comparability with the computations performed under electrochemical conditions, we studied the main discrepancies between polarized and unpolarized electronic structures of the models studied under vacuum conditions, on Figures S59-S64.

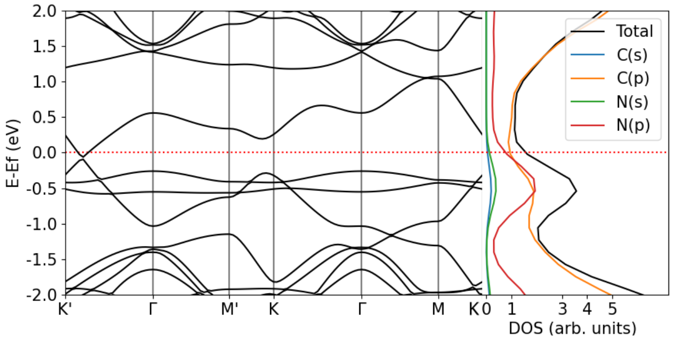

(a) Unpolarized case.

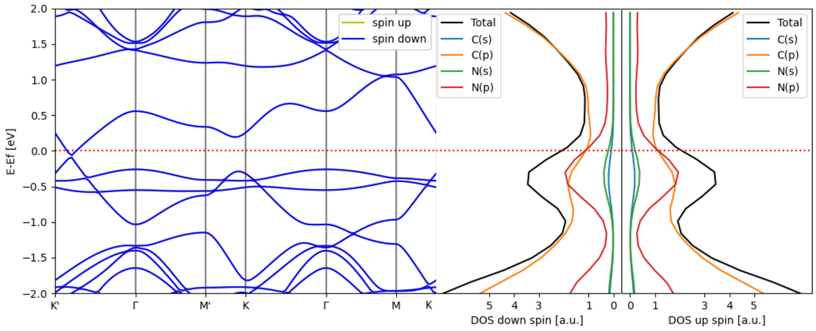

(b) Polarized case.

Fig. S59: Band structure, total and orbital projected DOS of N_4V_2 in its unpolarized and polarized state, respectively.

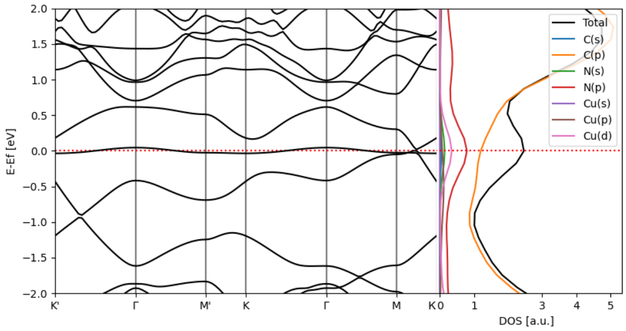

(a) Unpolarized case.

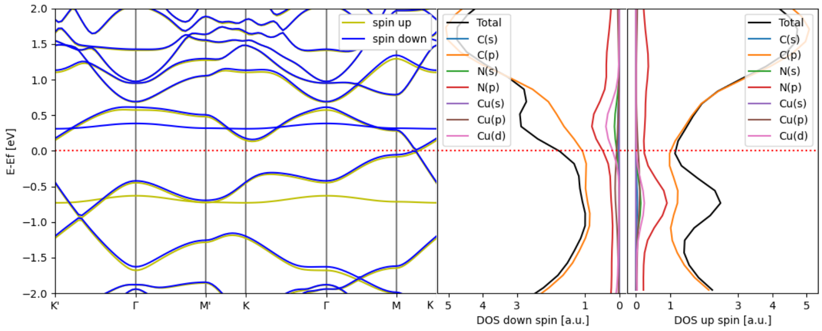

(b) Polarized case.

Fig. S60: Band structure, total and orbital projected DOS of N_4V_2-Cu in its unpolarized and polarized state, respectively.

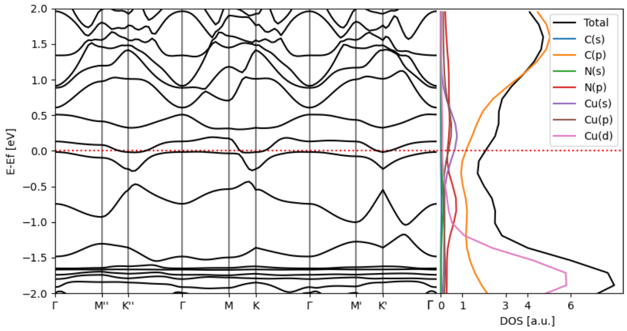

(a) Unpolarized case.

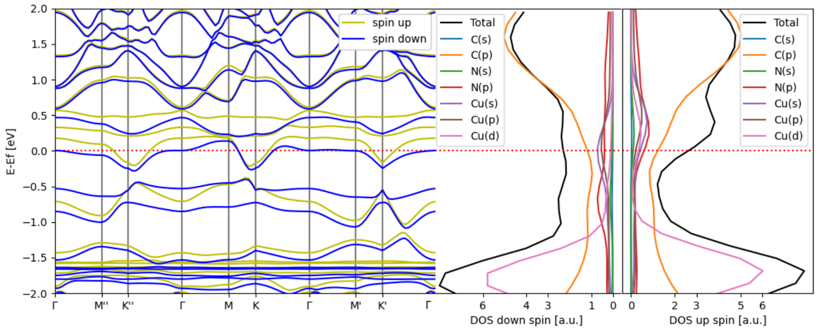

(b) Polarized case.

Fig. S61: Band structure, total and orbital projected DOS of N_4V_2-Cu_2 in its unpolarized and polarized state, respectively.

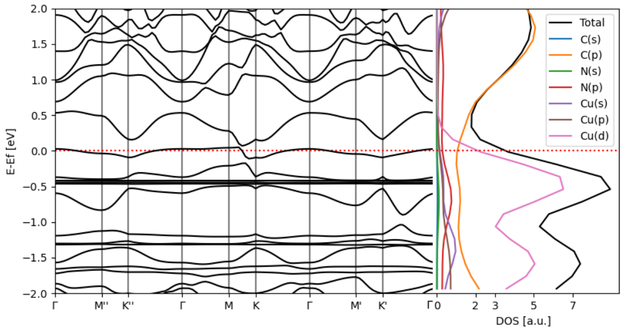

(a) Unpolarized case.

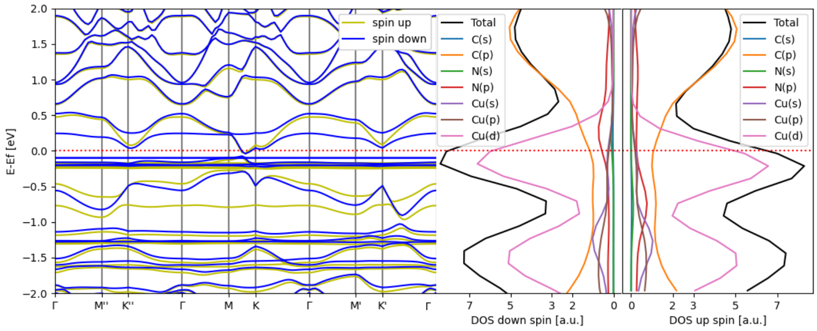

(b) Polarized case.

Fig. S62: Band structure, total and orbital projected DOS of N_4V_2-Cu_3 in its unpolarized and polarized state, respectively.

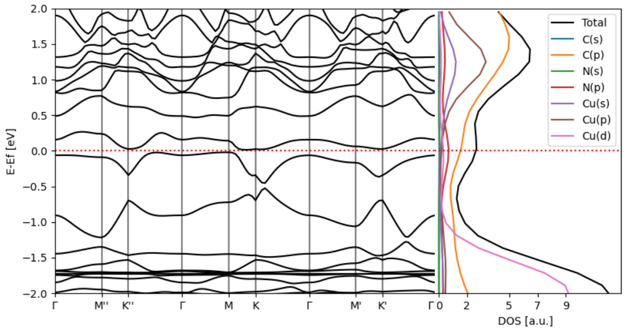

(a) Unpolarized case.

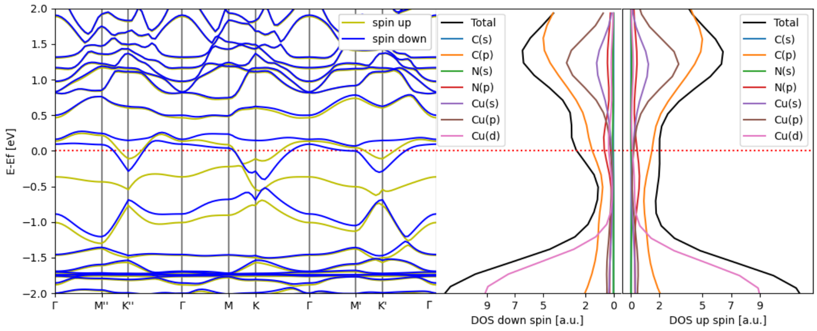

(b) Polarized case.

Fig. S63: Band structure, total and orbital projected DOS of N_4V_2-Cu_4 in its unpolarized and polarized state, respectively.

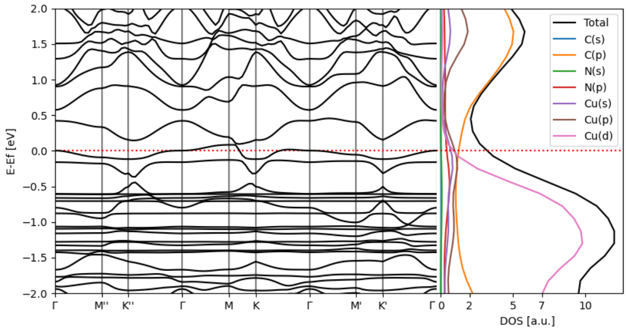

(a) Unpolarized case.

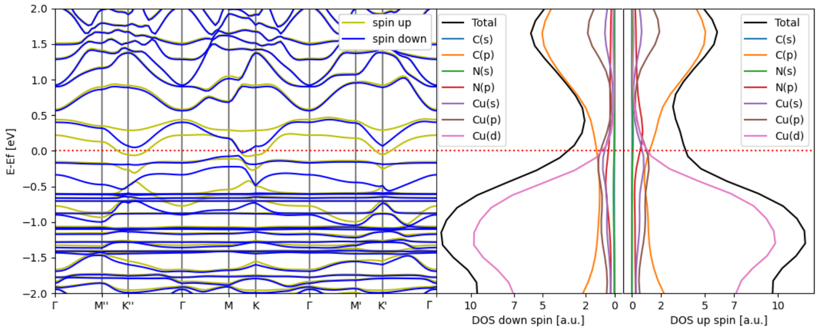

(b) Polarized case.

Fig. S64: Band structure, total and orbital projected DOS of N_4V_2-Cu_5 in its unpolarized and polarized state, respectively.

Figure S59 shows clearly, that there is no appreciable difference between polarized and unpolarized cases for N_4V_2, as the first line of Table S14 also witnesses. In the remaining cases a consistent trend can be observed, namely, the shape of the bands close to the Fermi-level are very similar comparing polarized and unpolarized states, only their position differs (the example of N_4V_2-Cu may present this mostly). This means, that our conclusions about the shape of the bands, the symmetries and phase transitions remain valid, i.e. we hope that we do not loose important information with the simplification of neglecting spin-polarization for computations in an electrochemical environment.

5.2 Electrochemical environment

The question of spin-polarization is non-trivial in the framework of the GCP-K theory, at finite electrode potential, since the assumed continuously excitable electronic states usually mean fractional number of electrons. As we showed in Section 5.1, the spin-contamination makes the spin-state of the certain atom indefinite under vacuum condition, then the fractional number of electrons complicates the situation further. Instead of

going deeply into theoretical reasoning, we analysed the deviance in free energy due to spin-polarization using the model N_4V_2-Cu in order to validate our results regarding to stability computations. As we discussed in Section 3.2 of the Main Text, N_4V_2-Cu showed a significant, 1.0 eV splitting in the defect state for different spins. We computed the absolute value of the difference between its polarized and unpolarized free energies with respect to electrode potential, the result is depicted on Figure S65.

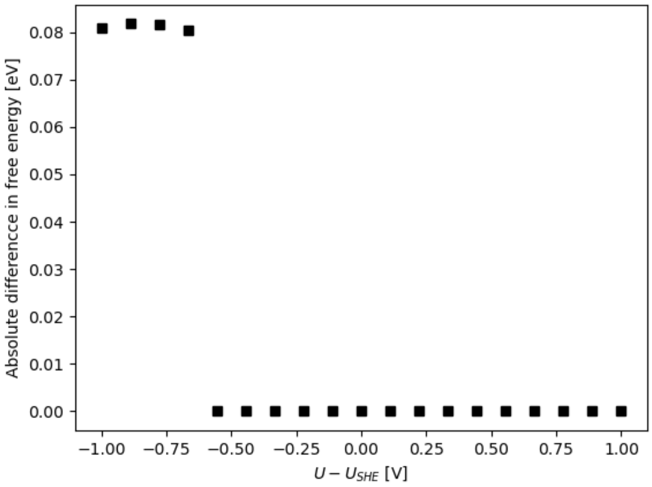

Fig. S65: The absolute difference between the polarized and unpolarized free energies of N_4V_2-Cu with respect to the electrode potential.

A spectacular drop can be observed at $U - U_{SHE} \approx -0.72$ V not by accident, as it is the PZC of N_4V_2-Cu. The reductive case means extra number of electrons, where the originally polarized ground state evolves to another, while the oxidative direction ends up most probably in unpolarized state (as the ground state of Cu^+ is singlet [31]). This is presumably the reason, why the difference in free energy reduces drastically in the latter case. Nevertheless, the maximum discrepancy is below 0.1 eV, even in case of $U - U_{SHE} = PZC$, where there is a well-defined difference between magnetizations in polarized ($M = \mu_B$) and unpolarized ($M = 0$) cases. It means, that the error occurring due to the lack of spin-polarization is negligible.

6 Comparison of electronic structure properties in vacuum and in an electrochemical environment

Here we present the comparison of the band structure and DOS of N_4V_2, furthermore N_4V_2-Cu_n ($n = 1 - 5$) to demonstrate the similarities and differences between these properties in vacuum and under electrochemical conditions. The simulations did not contain spin-polarization (not the true ground state in vacuum, still the main features of the band structures are well noticeable, see Sec. 5.1) and were performed using *GPAW* for models simulated under vacuum conditions and *jDFTx*, at $U - U_{SHE} = PZC$ for describing electrochemistry. The results are depicted on Figures S66-S71.

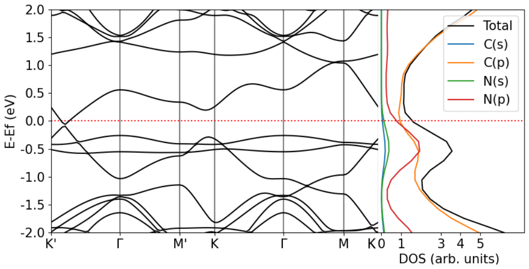

(a) Unpolarized state in vacuum.

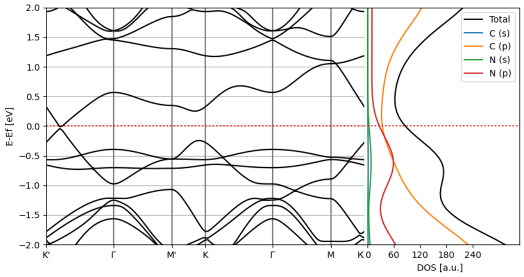

(b) $U - U_{SHE} = PZC$.

Fig. S66: Band structure, total and orbital projected DOS of N_4V_2 in its unpolarized ground and PZC states.

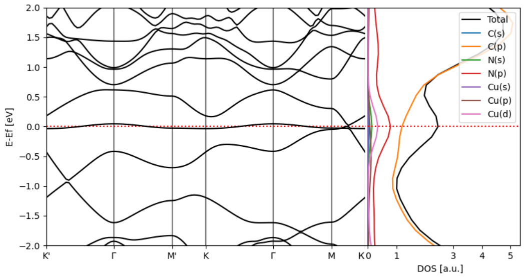

(a) Unpolarized state in vacuum.

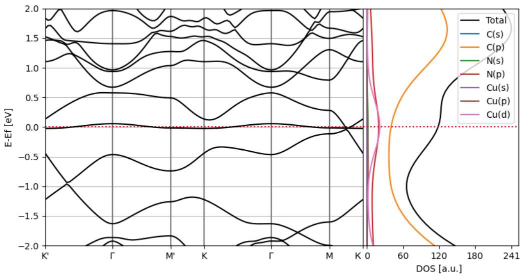

(b) $U - U_{SHE} = PZC$.

Fig. S67: Band structure, total and orbital projected DOS of N_4V_2-Cu in its unpolarized ground and PZC states.

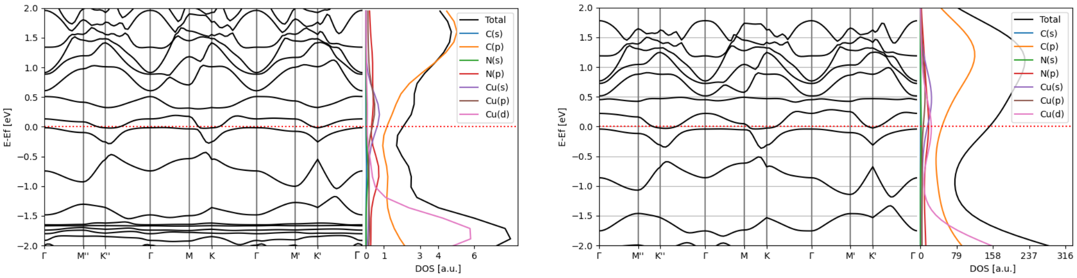

(a) Unpolarized state in vacuum. (b) $U - U_{SHE} = PZC$.

Fig. S68: Band structure, total and orbital projected DOS of N_4V_2-Cu_2 in its unpolarized ground and PZC states.

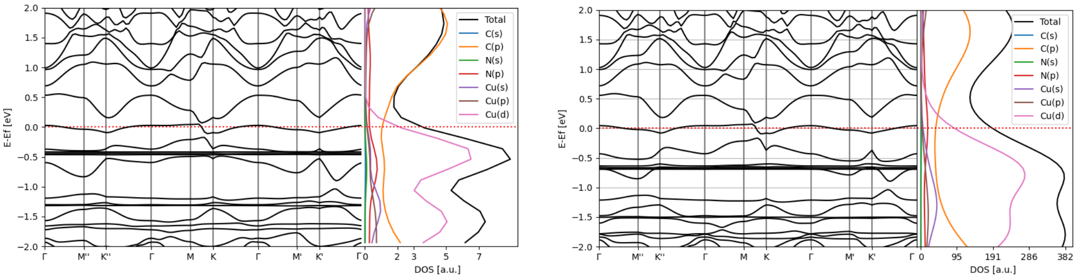

(a) Unpolarized state in vacuum. (b) $U - U_{SHE} = PZC$.

Fig. S69: Band structure, total and orbital projected DOS of N_4V_2-Cu_3 in its unpolarized ground and PZC states.

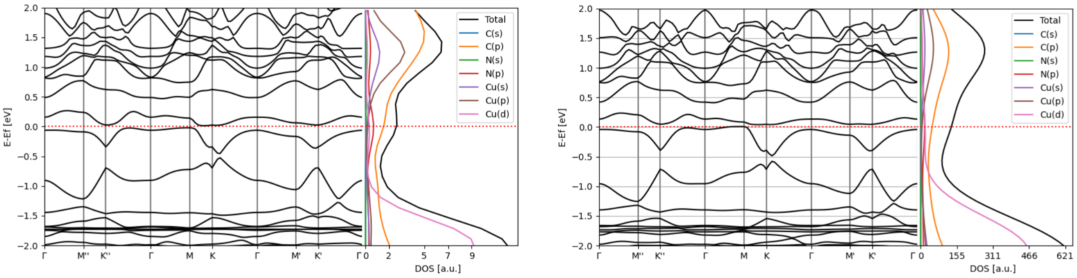

(a) Unpolarized state in vacuum. (b) $U - U_{SHE} = PZC$.

Fig. S70: Band structure, total and orbital projected DOS of N_4V_2-Cu_4 in its unpolarized ground and PZC states.

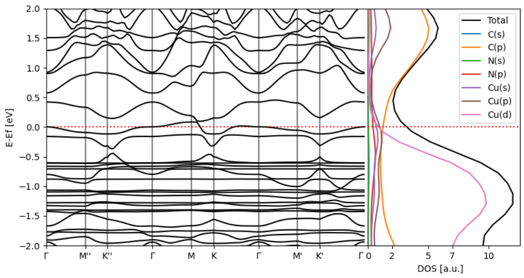

(a) Unpolarized state in vacuum.

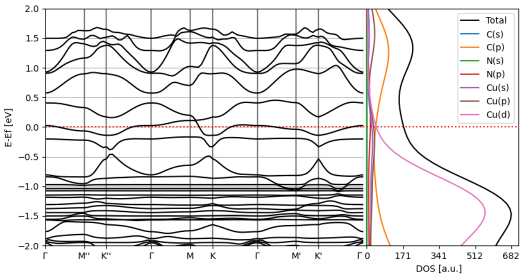

(b) $U - U_{SHE} = PZC$.

Fig. S71: Band structure, total and orbital projected DOS of N_4V_2-Cu_5 in its unpolarized ground and PZC states.

Figures S67-S71 point out two main differences appearing in the shape and position of the valence bands. The well identifiable flat bands, formed mostly by the d-orbitals of copper in the unpolarized ground state case, are shifted towards the conduction band compared to PZC case. Moreover, the PDOS of N_4V_2-Cu_3 shows more separated d-bands for models, studied under vacuum conditions, while the $jDFTx$ computations result in a more overlapped one despite, that same amount of broadening was employed (see Section 2 of Main Text). The first discrepancy occurs most probably due to the different type of basis sets. As we already mentioned in Sec. 2 of Main Text, LCAO-TZP basis set was applied for computations on models investigated under vacuum conditions, while $jDFTx$ uses only PW basis functions. Based on our experiences, the trend showing a positive shift in band energies for LCAO basis respect to computations on PW basis dominates over the effect of other conditions (e.g. electrolyte etc.). This seems to be confirmed by the comparison of LCAO and PW basis-based band structures, computed using GPAW and presented on Figure S72 for N_4V_2-Cu_2.

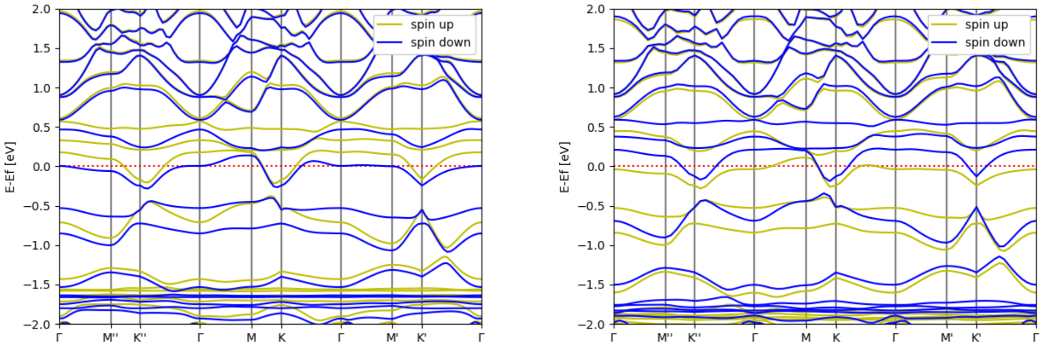

(a) Ground state band structure, computed on LCAO basis.

(b) Ground state band structure, computed on PW basis.

Fig. S72: The band structure of N_4V_2-Cu_2 computed on different bases.

The band energies, especially with spin up, demonstrate that there is an energy shift towards the conduction band. This is independent from the software and thus the pseudopotentials which have been applied (see below). Moreover, those states seem to be affected mostly, to which copper atoms have noticeable contribution, as Figure S69 demonstrates (for further discussion, see Section 3.4 of Main Text). The difference in the shape of PDOS occurs most likely as an effect of the various pseudopoptentials, i.e. GPAW uses PAWs [32], while for $jDFTx$ computations we selected ultrasoft ones [33]. The exact form of PDOS depends on the pseudo-atomic bases/orbitals - including semi-core and valence states - where to the many-electron state is projected.

7 Electronic structure properties of the studied models at different electrode potentials

As the example of N_4V_2-Cu showed in Section 3.4 of the Main Text, we computed the band structure, total and orbital projected DOS of N_4V_2-Cu_n $(n = 1-5)$ at electrode potentials $U - U_{SHE} = -1.0$, -0.5, 0.0 and PZC V, to examine how the band structures and DOS evolve. The results are depicted on Figure S73-S79. For the sake of completeness, we also included the cases of graphene and N_4V_2.

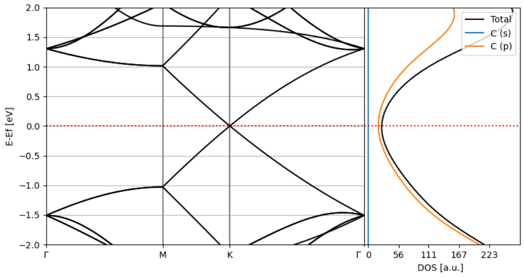

(a) $U - U_{SHE} = PZC = -0.72$ V.

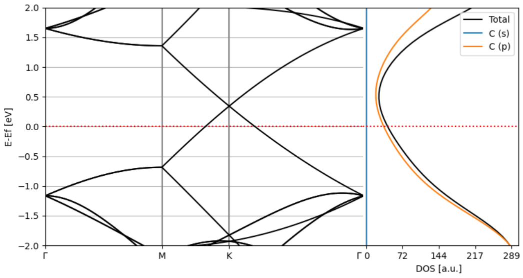

(b) $U - U_{SHE} = 0.0$ V.

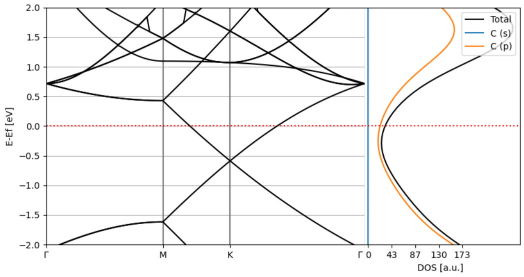

(c) $U - U_{SHE} = -0.5$ V.

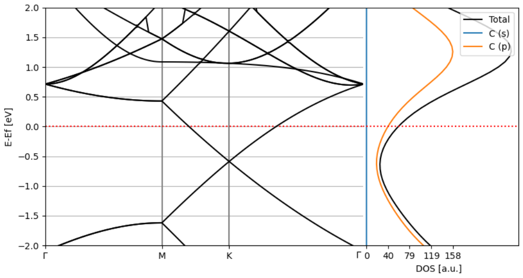

(d) $U - U_{SHE} = -1.0$ V.

Fig. S73: Band structure, total and orbital projected DOS of graphene at potentials $U - U_{SHE} = PZC = -0.72$, 0.0, -0.5 and -1.0 V.

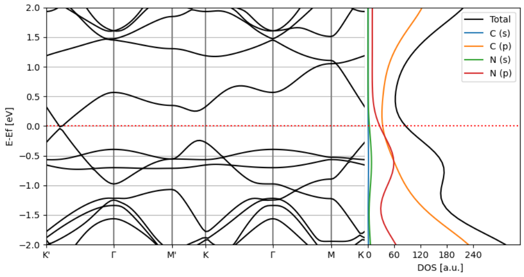

(a) $U - U_{SHE} = PZC = -0.72$ V.

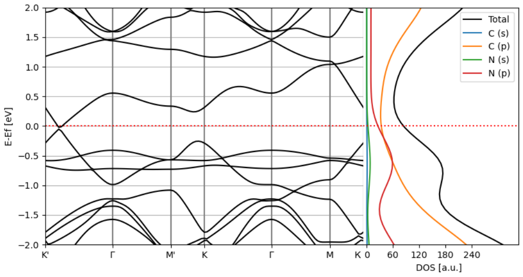

(b) $U - U_{SHE} = 0.0$ V.

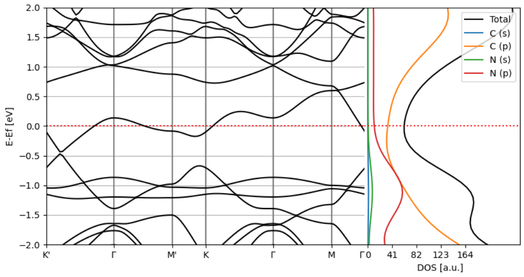

(c) $U - U_{SHE} = -0.5$ V.

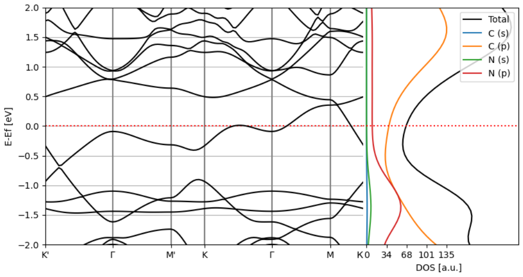

(d) $U - U_{SHE} = -1.0$ V.

Fig. S74: Band structure, total and orbital projected DOS of N_4V_2 at potentials $U - U_{SHE} = PZC = -0.72$, 0.0, -0.5 and -1.0 V.

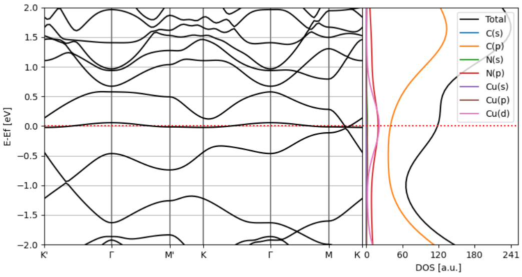

(a) $U - U_{SHE} = PZC = -0.72$ V.

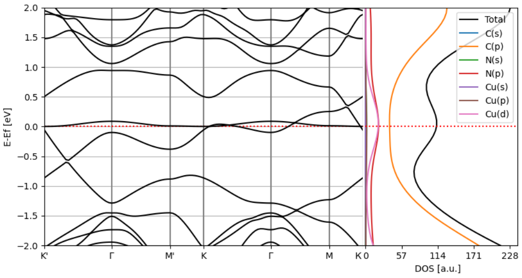

(b) $U - U_{SHE} = 0.0$ V.

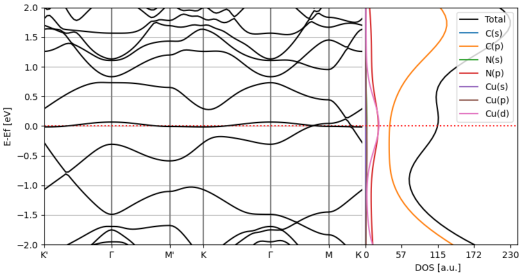

(c) $U - U_{SHE} = -0.5$ V.

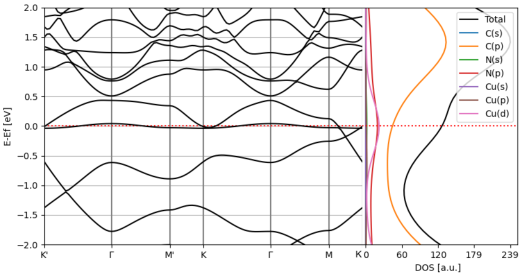

(d) $U - U_{SHE} = -1.0$ V.

Fig. S75: Band structure, total and orbital projected DOS of N_4V_2-Cu at potentials $U - U_{SHE} = PZC = -0.72$, 0.0, -0.5 and -1.0 V.

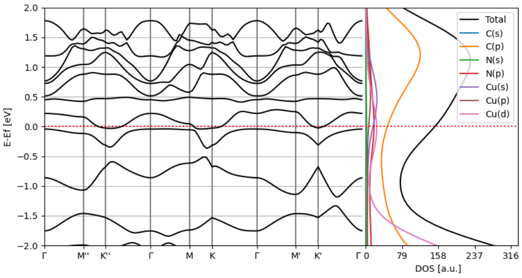

(a) $U - U_{SHE} = PZC = -0.86$ V.

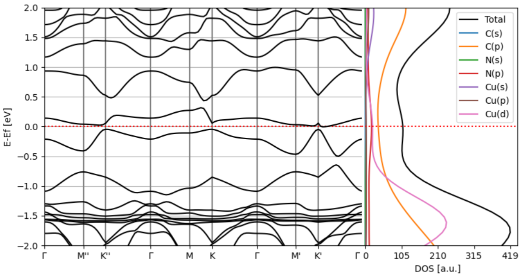

(b) $U - U_{SHE} = 0.0$ V.

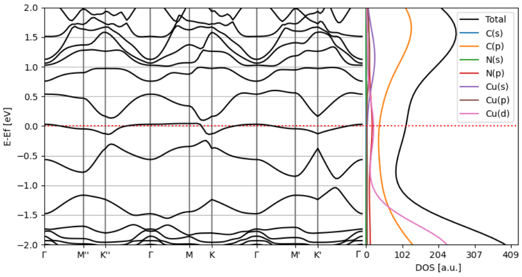

(c) $U - U_{SHE} = -0.5$ V.

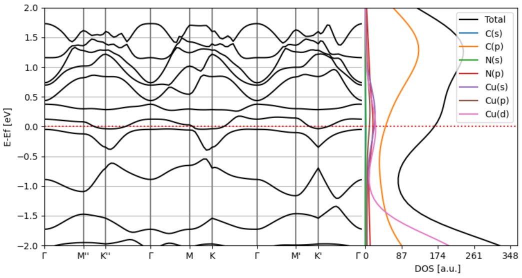

(d) $U - U_{SHE} = -1.0$ V.

Fig. S76: Band structure, total and orbital projected DOS of N_4V_2-Cu_2 at potentials $U - U_{SHE} = PZC = -0.86$, 0.0, -0.5 and -1.0 V.

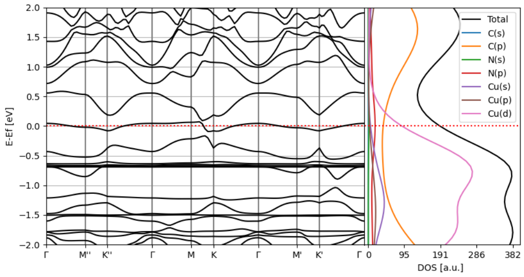

(a) $U - U_{SHE} = PZC = -0.69$ V.

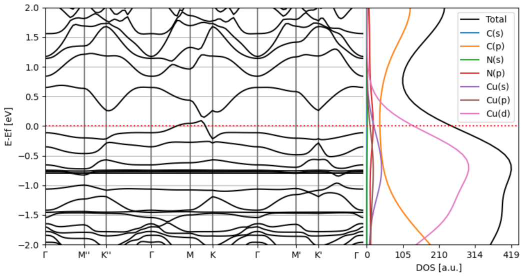

(b) $U - U_{SHE} = 0.0$ V.

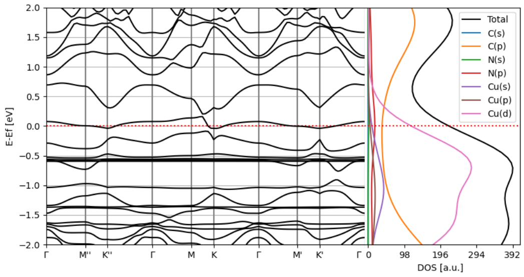

(c) $U - U_{SHE} = -0.5$ V.

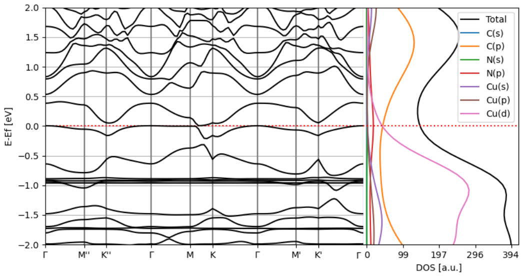

(d) $U - U_{SHE} = -1.0$ V.

Fig. S77: Band structure, total and orbital projected DOS of N_4V_2-Cu_3 at potentials $U - U_{SHE} = PZC = -0.69$, 0.0, -0.5 and -1.0 V.

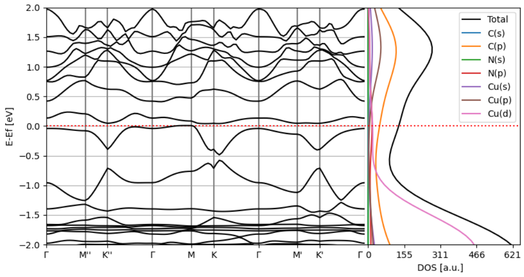

(a) $U - U_{SHE} = PZC = -0.97$ V.

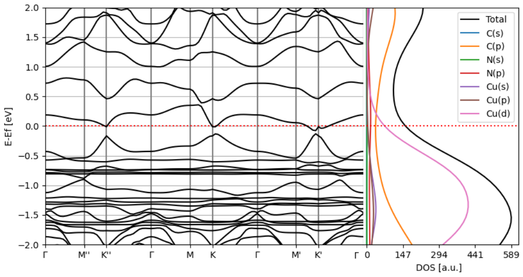

(b) $U - U_{SHE} = 0.0$ V.

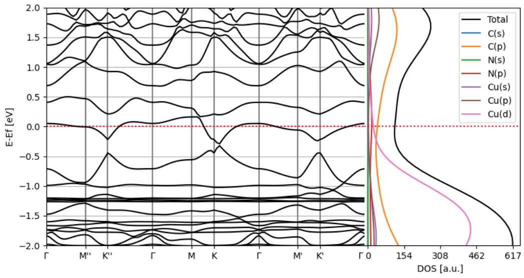

(c) $U - U_{SHE} = -0.5$ V.

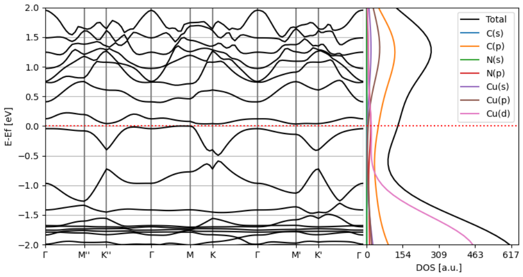

(d) $U - U_{SHE} = -1.0$ V.

Fig. S78: Band structure, total and orbital projected DOS of N_4V_2-Cu_4 at potentials $U - U_{SHE} = PZC = -0.97$, 0.0, -0.5 and -1.0 V.

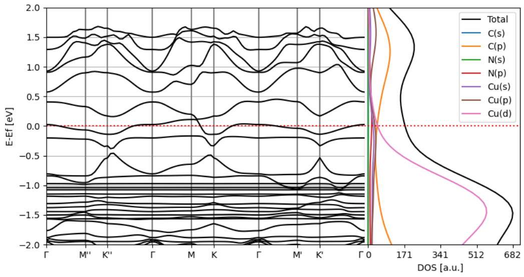

(a) $U - U_{SHE} = PZC = -0.83$ V.

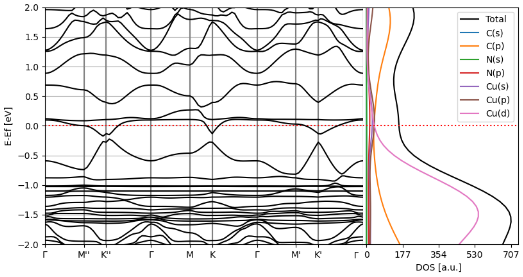

(b) $U - U_{SHE} = 0.0$ V.

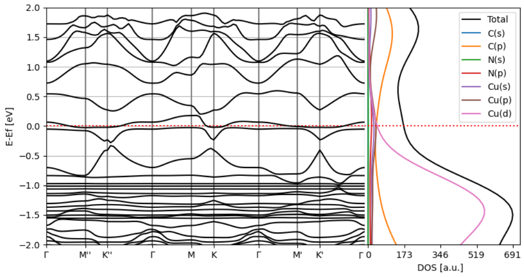

(c) $U - U_{SHE} = -0.5$ V.

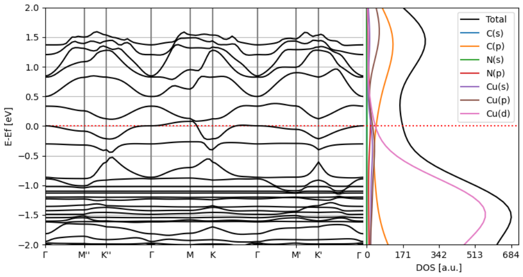

(d) $U - U_{SHE} = -1.0$ V.

Fig. S79: Band structure, total and orbital projected DOS of N_4V_2-Cu_5 at potentials $U - U_{SHE} = PZC = -0.83$, 0.0, -0.5 and -1.0 V.

Figures S73 and S74 clearly reflect the concept of rigid electronic structure, discussed in Section 3.4 of Main Text, meaning that the shape of band structure and DOS is not modified by the potentials, only the Fermi-level is shifted according to the relation between the U_{SHE} and the PZC of the model. The bands, partially formed by the orbitals (mainly d-type) of copper atoms, slightly deform, especially those located near the Fermi-level. Additionally, a shift in energy can be observed for the spatially localized (flat bands) states. Therefore, using the electrode potential, not only the type and density of the charge carriers (curvature of the partially occupied band and the value of the DOS), but the shape of the band, i.e. the conductivity (Fermi-velocity) can be adjusted as well.

References

[1] Pablo Jaque and Alejandro Toro-Labbé. "Characterization of copper clusters through the use of density functional theory reactivity descriptors". In: *The Journal of Chemical Physics* 117.7 (2002), pp. 3208–3218. DOI: 10.1063/1.1493178. eprint: https://doi.org/10.1063/1.1493178. URL: https://doi.org/10.1063/1.1493178.

[2] Pablo Jaque and Alejandro Toro-Labbé. "Polarizability of neutral copper clusters". In: *Journal of Molecular Modeling* 20.9 (2014), p. 2410. ISSN: 0948-5023. DOI: 10.1007/s00894-014-2410-6. URL: https://doi.org/10.1007/s00894-014-2410-6.

[3] Huilin Tao et al. "What Is the Best Size of Subnanometer Copper Clusters for CO2 Conversion to Methanol at Cu/TiO2 Interfaces? A Density Functional Theory Study". In: *J. Phys. Chem. C* 123.39 (Oct. 2019), pp. 24118–24132. ISSN: 1932-7447. DOI: 10.1021/acs.jpcc.9b06947. URL: https://doi.org/10.1021/acs.jpcc.9b06947.

[4] Francesco Buonocore, Nicola Lisi, and Olivia Pulci. "Electronic and optical properties of metal decorated nitrogen-doped vacancy defects in graphene". In: *Journal of Physics: Condensed Matter* 31.23 (Apr. 2019), p. 235302. DOI: 10.1088/1361-648X/ab0bf9. URL: https://dx.doi.org/10.1088/1361-648X/ab0bf9.

[5] Yoshitaka Fujimoto and Susumu Saito. "Formation, stabilities, and electronic properties of nitrogen defects in graphene". In: *Phys. Rev. B* 84 (24 Dec. 2011), p. 245446. DOI: 10.1103/PhysRevB.84.245446. URL: https://link.aps.org/doi/10.1103/PhysRevB.84.245446.

[6] Shyam Kattel, Plamen Atanassov, and Boris Kiefer. "Stability, Electronic and Magnetic Properties of In-Plane Defects in Graphene: A First-Principles Study". In: *The Journal of Physical Chemistry C* 116.14 (2012), pp. 8161–8166. DOI: 10.1021/jp2121609. eprint: https://doi.org/10.1021/jp2121609. URL: https://doi.org/10.1021/jp2121609.

[7] John P. Perdew, Kieron Burke, and Matthias Ernzerhof. "Generalized Gradient Approximation Made Simple". In: *Phys. Rev. Lett.* 77 (18 Oct. 1996), pp. 3865–3868. DOI: 10.1103/PhysRevLett.77.3865. URL: https://link.aps.org/doi/10.1103/PhysRevLett.77.3865.

[8] Stefan Grimme et al. "A consistent and accurate ab initio parametrization of density functional dispersion correction (DFT-D) for the 94 elements H-Pu". In: *The Journal of Chemical Physics* 132.15 (2010), p. 154104. DOI: 10.1063/1.3382344. eprint: https://doi.org/10.1063/1.3382344. URL: https://doi.org/10.1063/1.3382344.

[9] Erik Bitzek et al. "Structural Relaxation Made Simple". In: *Phys. Rev. Lett.* 97 (17 Oct. 2006), p. 170201. DOI: 10.1103/PhysRevLett.97.170201. URL: https://link.aps.org/doi/10.1103/PhysRevLett.97.170201.

[10] Hendrik J. Monkhorst and James D. Pack. "Special points for Brillouin-zone integrations". In: *Phys. Rev. B* 13 (12 June 1976), pp. 5188–5192. DOI: 10.1103/PhysRevB.13.5188. URL: https://link.aps.org/doi/10.1103/PhysRevB.13.5188.

[11] Márton Guba and Tibor Höltzl. *Stability and Electronic Structure of nitrogen-doped graphene-supported Cu_n ($n = 1 - 5$) clusters in Vacuum and under Electrochemical Conditions: towards sensor and catalyst design.* Accessed on August, 31th, 2023. DOI: 10.5281/zenodo.8272188. URL: https://doi.org/10.5281/zenodo.8272188.

[12] GPAW. *Introduction.* Accessed on December, 6th, 2023. URL: https://wiki.fysik.dtu.dk/gpaw/documentation/basic.html.

[13] Ravishankar Sundararaman et al. "JDFTx: Software for joint density-functional theory". In: *SoftwareX* 6 (2017), pp. 278–284. ISSN: 2352-7110. DOI: https://doi.org/10.1016/j.softx.2017.10.006. URL: https://www.sciencedirect.com/science/article/pii/S2352711017300559.

[14] Graeme Henkelman, Andri Arnaldsson, and Hannes Jónsson. "A fast and robust algorithm for Bader decomposition of charge density". In: *Computational Materials Science* 36.3 (2006), pp. 354–360. ISSN: 0927-0256. DOI: https://doi.org/10.1016/j.commatsci.2005.04.010. URL: https://www.sciencedirect.com/science/article/pii/S0927025605001849.

[15] Edward Sanville et al. "Improved grid-based algorithm for Bader charge allocation". In: *Journal of Computational Chemistry* 28.5 (2007), pp. 899–908. DOI: https://doi.org/10.1002/jcc.20575.

[16] W Tang, E Sanville, and G Henkelman. "A grid-based Bader analysis algorithm without lattice bias". In: *Journal of Physics: Condensed Matter* 21.8 (Jan. 2009), p. 084204. DOI: 10.1088/0953-8984/21/8/084204. URL: https://dx.doi.org/10.1088/0953-8984/21/8/084204.

[17] Henkelmann-group. *Bader charge analysis*. Accessed on December, 6th, 2023. URL: http://theory.cm.utexas.edu/henkelman/code/bader/.

[18] Pavel Hobza and Klaus Müller-Dethlefs. "Foreword". In: *Non-covalent Interactions: Theory and Experiment*. The Royal Society of Chemistry, Nov. 2009. ISBN: 978-1-84755-853-4. DOI: 10.1039/9781847559906-FP005. URL: https://doi.org/10.1039/9781847559906-FP005.

[19] S.F. Boys and F. Bernardi. "The calculation of small molecular interactions by the differences of separate total energies. Some procedures with reduced errors". In: *Molecular Physics* 19.4 (1970), pp. 553–566. DOI: 10.1080/00268977000101561. eprint: https://doi.org/10.1080/00268977000101561. URL: https://doi.org/10.1080/00268977000101561.

[20] A. H. Castro Neto et al. "The electronic properties of graphene". In: *Rev. Mod. Phys.* 81 (1 Jan. 2009), pp. 109–162. DOI: 10.1103/RevModPhys.81.109. URL: https://link.aps.org/doi/10.1103/RevModPhys.81.109.

[21] Zhufeng Hou et al. "Electronic structure of N-doped graphene with native point defects". In: *Phys. Rev. B* 87 (16 Apr. 2013), p. 165401. DOI: 10.1103/PhysRevB.87.165401. URL: https://link.aps.org/doi/10.1103/PhysRevB.87.165401.

[22] GPAW. *Doucumentation of GPAW: Introduction*. Accessed on December, 6th, 2023. URL: https://wiki.fysik.dtu.dk/gpaw/documentation/basic.html.

[23] Yufeng Huang, Robert J. Nielsen, and William A. III Goddard. "Reaction Mechanism for the Hydrogen Evolution Reaction on the Basal Plane Sulfur Vacancy Site of MoS2 Using Grand Canonical Potential Kinetics". In: *Journal of the American Chemical Society* 140.48 (2018). PMID: 30406657, pp. 16773–16782. DOI: 10.1021/jacs.8b10016. eprint: https://doi.org/10.1021/jacs.8b10016. URL: https://doi.org/10.1021/jacs.8b10016.

[24] Md Delowar Hossain, Zhengtang Luo, and W. a. Goddard. "Grand Canonical Potential Kinetics of CO2 Reduction Reaction over Graphene-Supported Single-Atom Catalysts". In: *ECS Meeting Abstracts* MA2019-02.22 (Sept. 2019), p. 1071. DOI: 10.1149/MA2019-02/22/1071. URL: https://dx.doi.org/10.1149/MA2019-02/22/1071.

[25] William A. Goddard and Jie Song. "Grand Canonical Quantum Mechanics with Applications to Mechanisms and Rates for Electrocatalysis". In: *Topics in Catalysis* (2023). ISSN: 1572-9028. DOI: 10.1007/s11244-023-01794-8. URL: https://doi.org/10.1007/s11244-023-01794-8.

[26] Roger Fletcher. *Practical methods of optimization.* Ed. by John Wiley and Sons. Chichester ; New York : Wiley, 1987. ISBN: 978-0-471-91547-8.

[27] Pratim Kumar Chattaraj, ed. *Aromaticity and Metal Clusters.* 1st ed. CRC Press., Oct. 2010. ISBN: 9780429130991. DOI: https://doi.org/10.1201/EBK1439813348.

[28] Erik Bhekti Yutomo, Fatimah Arofiati Noor, and Toto Winata. "Effect of the number of nitrogen dopants on the electronic and magnetic properties of graphitic and pyridinic N-doped graphene – a density-functional study". In: *RSC Adv.* 11 (30 2021), pp. 18371–18380. DOI: 10.1039/D1RA01095F. URL: http://dx.doi.org/10.1039/D1RA01095F.

[29] Jon Baker, Andrew Scheiner, and Jan Andzelm. "Spin contamination in density functional theory". In: *Chemical Physics Letters* 216.3 (1993), pp. 380–388. ISSN: 0009-2614. DOI: https://doi.org/10.1016/0009-2614(93)90113-F. URL: https://www.sciencedirect.com/science/article/pii/000926149390113F.

[30] John A. Pople, Peter M. W. Gill, and Nicholas C. Handy. "Spin-unrestricted character of Kohn-Sham orbitals for open-shell systems". In: *International Journal of Quantum Chemistry* 56.4 (1995), pp. 303–305. DOI: https://doi.org/10.1002/qua.560560414. eprint: https://onlinelibrary.wiley.com/doi/pdf/10.1002/qua.560560414. URL: https://onlinelibrary.wiley.com/doi/abs/10.1002/qua.560560414.

[31] NIST. *Atomic Spectra Database Levels Data of Cu.* Accessed on December, 6th, 2023. URL: https://physics.nist.gov/cgi-bin/ASD/energy1.pl?de=0&spectrum=Cu+II&submit=Retrieve+Data&units=0&format=0&output=0&page_size=15&multiplet_ordered=0&conf_out=on&term_out=on&level_out=on&biblio=on&temp=.

[32] GPAW. *PAW datasets.* Accessed on December, 6th, 2023. URL: https://wiki.fysik.dtu.dk/gpaw/setups/setups.html#installation-of-paw-datasets.

[33] Kevin F. Garrity et al. "Pseudopotentials for high-throughput DFT calculations". In: *Computational Materials Science* 81 (2014), pp. 446–452. ISSN: 0927-0256. DOI: https://doi.org/10.1016/j.commatsci.2013.08.053. URL: https://www.sciencedirect.com/science/article/pii/S0927025613005077.
